# Evaluation of the Therapeutic Potential of Sulfonyl Urea Derivatives as Soluble Epoxide Hydrolase (sEH) Inhibitors

**DOI:** 10.3390/molecules29133036

**Published:** 2024-06-26

**Authors:** Biswajit Kundu, Szabolcs Dvorácskó, Abhishek Basu, Lenny Pommerolle, Kyu Ah Kim, Casey M. Wood, Eve Gibbs, Madeline Behee, Nadya I. Tarasova, Resat Cinar, Malliga R. Iyer

**Affiliations:** 1 Section on Medicinal Chemistry, National Institute on Alcohol Abuse and Alcoholism (NIAAA), National Institutes of Health (NIH), 5625 Fishers Lane, Rockville, MD 20852, USA; 2 Section on Fibrotic Disorders, National Institute on Alcohol Abuse and Alcoholism (NIAAA), National Institutes of Health (NIH), 5625 Fishers Lane, Rockville, MD 20852, USA; 3 Cancer Innovation Laboratory, Center for Cancer Research, National Cancer Institute (NCI), National Institutes of Health (NIH), P.O. Box B, Frederick, MD 21702, USA

**Keywords:** soluble epoxide hydrolase (sEH), epoxyeicosatrienoic acids (EETs), structure-activity relationship (SAR), in vitro ADME, molecular docking, acute lung injury (ALI)

## Abstract

The inhibition of soluble epoxide hydrolase (sEH) can reduce the level of dihydroxyeicosatrienoic acids (DHETs) effectively maintaining endogenous epoxyeicosatrienoic acids (EETs) levels, resulting in the amelioration of inflammation and pain. Consequently, the development of sEH inhibitors has been a prominent research area for over two decades. In the present study, we synthesized and evaluated sulfonyl urea derivatives for their potential to inhibit sEH. These compounds underwent extensive in vitro investigation, revealing their potency against human and mouse sEH, with **4f** showing the most promising sEH inhibitory potential. When subjected to lipopolysaccharide (LPS)-induced acute lung injury (ALI) in studies in mice, compound **4f** manifested promising anti-inflammatory efficacy. We investigated the analgesic efficacy of sEH inhibitor **4f** in a murine pain model of tail-flick reflex. These results validate the role of sEH inhibition in inflammatory diseases and pave the way for the rational design and optimization of sEH inhibitors based on a sulfonyl urea template.

## 1. Introduction

The pathophysiological process of inflammation is intricate and involves a wide range of mediators and signaling pathways [1,2]. Inflammation frequently manifests physiologically as redness, swelling, fever, pain, and even loss of organ function [3]. Research conducted over the past few decades has revealed that the arachidonic acid (AA) pathway produces bioactive lipid mediators that control a variety of inflammatory processes [4,5,6]. Mammals metabolize polyunsaturated fatty acids like arachidonic acid through the action of cytochrome P450s (CYPs), lipoxygenases (LOXs), and cyclooxygenases (COXs) [4]. These are the three principal enzymatic pathways that control the formation of eicosanoids from arachidonic acid. The COX and LOX pathways, which primarily produce pro-inflammatory lipid mediators such as prostaglandins and leukotrienes, have been extensively studied and pharmaceutically targeted [7]. On the other hand, the CYP route generates lipid mediators that are both pro- and anti-inflammatory, like the potent anti-inflammatory epoxyeicosatrienoic acids (EETs) and the pro-inflammatory 20-hydroxyeicosatetraenoic acid [8,9,10]. The enzyme soluble epoxide hydrolase (sEH) plays a pivotal role in the metabolism process of bioactive lipid signaling molecules [11,12]. The substrate-specific hydrolase activity of sEH transforms EETs to the corresponding dihydroxyeicosatrienoic acids (DHETs) purported to have inflammatory effects [13,14].

The cytochrome P450 pathway has received a lot of interest lately. EETs are cytochrome P450-derived eicosanoids that can cause vasodilation and inhibit the cytokine-induced inflammatory response in the vasculature, heart, and kidney [15,16,17]. EETs are recognized as crucial signaling elements in an organism and have biological effects including blood pressure, vasodilation, and anti-inflammation. It has been demonstrated that sEH inhibition leads to elevated levels of EETs, subsequently manifesting anti-inflammatory, analgesic, and antihypertensive effects [11,12,18]. Given the opioid crisis, there is an urgent need for novel medications to address both neuropathic pain and chronic pain without having the same addictive qualities as opioids. Soluble epoxide hydrolase inhibitors stabilize epoxides of polyunsaturated fatty acids (EpFAs), which are endogenous mediators that alleviate inflammation and lessen pain, by acting on the cytochrome P450 branch of the arachidonate cascade. Pharmacological inhibition of sEH in animal models exhibited beneficial effects on the treatment of hypertension, inflammation, and neuropathic pain [19,20,21]. For improving the positive effects of EpFAs in a variety of disorders, including neuropathic pain, hypertension, inflammatory bowel disease, and cardiovascular disease, sEH is thought to be an important therapeutic target [22,23,24,25,26,27].

**Figure 1:**
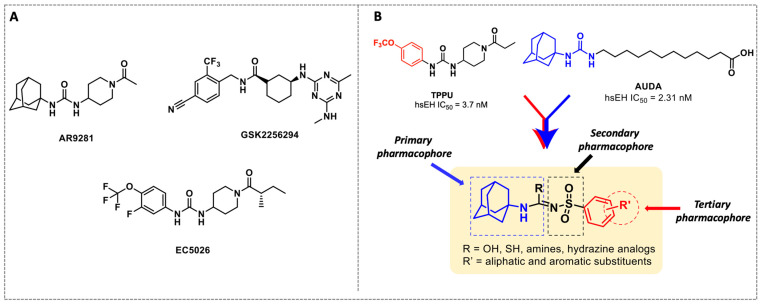
(**A**) Chemical structures of sEH inhibitors in clinical trials. (**B**) Our rational design of sulfonyl urea scaffold containing requisite features exhibiting sEH inhibition.

X-ray crystallographic studies demonstrated that sEH has an L-shaped active pocket with the catalytic residues situated at the corner, and the entire pocket is essentially hydrophobic [28,29]. Hence, a large number of very potent sEH inhibitors (sEHis) possess lipophilic moieties. Arete Therapeutics developed AR9281 to treat hypertension in diabetic individuals; however, due in significant part to its poor pharmacokinetic features, the drug was not progressed beyond a phase II human clinical trial [30]. GlaxoSmithKline’s GSK2256294, which was created for chronic obstructive pulmonary disease, is currently undergoing clinical studies for use in obese smokers as well as additional indications such as subarachnoidal hemorrhage and people with diabetes who are insulin resistant (Figure 1A) [31]. EicOsis has more recently substituted an aromatic ring for the adamantyl moiety of AR9281 in their drug candidate EC5026, which has completed phase I-a clinical trials in humans for the treatment of neuropathic pain (Figure 1A) [32]. Using urea-based motifs with lipophilic units of varying sizes, we recently found that urea derivatives bearing aryl sulfonyl groups manifested subnanomolar inhibition of the human sEH (*h*sEH) for certain compounds. Therefore, herein, we report a detailed investigation into sulfonyl urea derivatives and their suitability as new hits for sEH inhibition. 

## 2. Chemistry

**Scheme 1:**
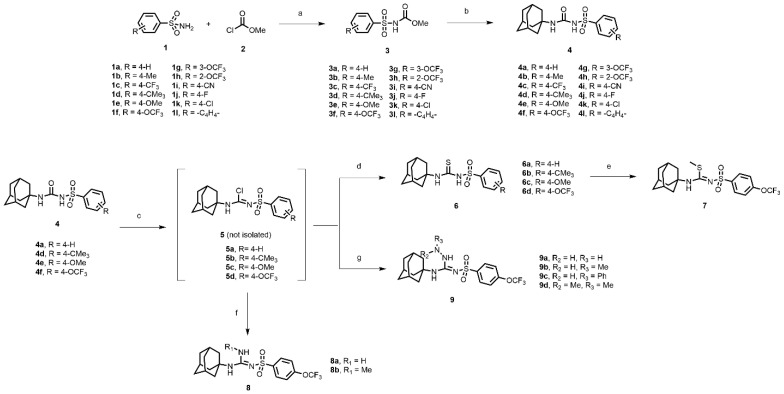
Reagents and conditions: (a) triethylamine, acetonitrile, rt, 12 h; (b) 1-adamantylamine, toluene, reflux, 4 h; (c) phosphorus oxychloride, N, N-Diisopropylethylamine, toluene, reflux, 2 h; (d) Na_2_S_2_O_3_, dioxane: H_2_O (8:1), 85–90 °C, 1–2 h; (e) iodomethane, rt, 15 min; (f) ammonia in methanol, dichloromethane, rt, 2 h (for **8a**); methylamine hydrochloride, triethylamine, dichloromethane: methanol (8:2), rt, 2 h (for **8b**); (g) hydrazine monohydrate, dichloromethane, rt, 2 h (for **9a**); methylhydrazine, dichloromethane, rt, 2 h (for **9b**); phenylhydrazine, dichloromethane, rt, 2 h (for **9c**); dimethylhydrazine, dichloromethane, rt, 2 h (for **9d**).

In this study, we report in detail the design, synthesis, and biological characterization of sEH inhibitors by utilizing an alkyl amino sulfonyl urea attachment. As seen in Figure 1B, the pharmacophoric features of AUDA and TPPU can be merged to generate sulfonamide-based compounds for their potential to inhibit sEH. Recently, glimepiride, a sulfonyl urea compound, was reported to have sEH inhibitory properties, although previously, sulfonyl ureas were shown to not have sEH inhibition [33,34]. Sulfonyl carbamates could be generated from various sulfonamides (**1a–l**) by reaction with methyl chloroformate, as previously reported [35]. Sulfonyl carbamates (**3a–l**), upon treatment with 1-adamantylamine in refluxing toluene followed by evaporation and trituration, resulted in white solid sulfonylurea products (**4a–l**). Following our previously published protocol, compound sulfonyl ureas of type **4** could be converted into sulfonyl thioureas in a one-pot manner using N, N-diisopropylethylamine (DIPEA) and phosphoryl chloride (POCl_3_) followed by treatment with sodium thiosulfate dissolved in a mixture of dioxane and water (8:1) to afford **6a–d** as white solids [36]. Compound **6d** was processed to **7** by the treatment of iodomethane in the same pot (Scheme 1). While maintaining the 4-OCF_3_ group, compound **4f** was converted to **8a** and **8b** using 7M ammonia solution in methanol and methyl amine, respectively, by treatment of the imidoyl chloride intermediate obtained from treatment of DIPEA/POCl_3_ in toluene. In a parallel set of reactions, the imidoyl chloride intermediate was dissolved in DCM and was treated with various hydrazine moieties. The reaction mixtures were stirred at room temperature for 2 h to afford several hydrazine derivatives, **9a–d** (Scheme 1).

**Scheme 2:**
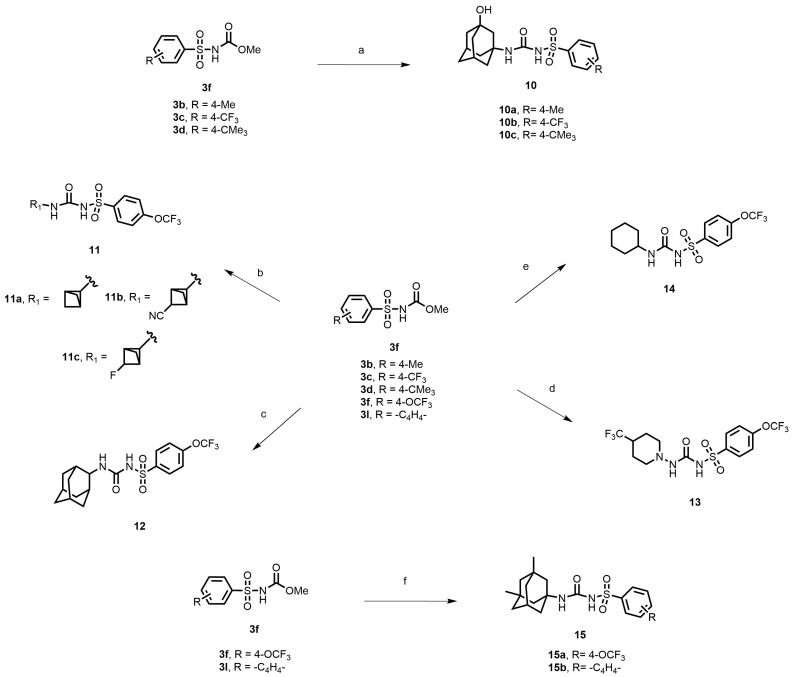
Reagents and conditions: (a) 3-aminoadamantan-1-ol, toluene, reflux, 4 h; (b) various bicycloamines, triethylamine, toluene, 100 °C, 3 h; (c) adamantan-2-amine, toluene, reflux, 4 h; (d) 4-(trifluoromethyl)piperidine, toluene, reflux, 4 h; (e) cyclohexylamine, toluene, reflux, 4 h; (f) memantine hydrochloride, triethylamine, toluene, reflux, 4 h.

**Scheme 3:**
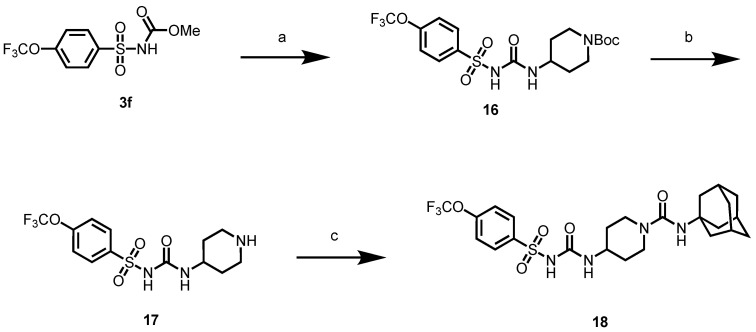
Reagents and conditions: (a) *tert*-butyl 4-aminopiperidine-1-carboxylate, toluene, reflux, 2 h; (b) trifluoroacetic acid, DCM, rt, 2 h; (c) 1-isocyanatoadamantane, triethylamine, DMF, rt, 12 h.

In another set of compounds, we modified the earlier route and attached various alkyl amines as depicted in Scheme 2. Sulfonyl carbamates **3b–d** were treated with aminoadamantan-1-ol in refluxing toluene to yield sulfonyl ureas **10a–c**. Compound **3f** was subjected to react with a set of various alkylamines including bicycloamines, adamantan-2-amine, 4-(trifluoromethyl)piperidine, cyclohexylamine, and memantine hydrochloride to form a series of ureas (compounds **11a–d**, **12–14**, and **15a–b**) following a previous protocol (Scheme 2). In another scheme of reactions, compound **3f** was refluxed with tert-butyl 4-aminopiperidine-1-carboxylate to form urea **16** (Scheme 3). Deprotection of the Boc group by trifluoroacetic acid (TFA) led to compound **17**, which was further reacted with 1-isocyanatoadamantane in the presence of triethylamine in DMF solvent to afford compound **18**, containing two urea groups (Scheme 3). 

## 3. Results and Discussion

### 3.1. Activity-Guided Design of sEH Inhibitors

The rational development of the sEH inhibitors was guided by their activity profile as measured in in vitro assays through a structure–activity relationship (SAR). The mammalian sEH is a bifunctional enzyme containing an N-terminal phosphatase region and a C-terminal hydrolase domain [37,38]. The hydrolysis process of EETs is catalyzed by the C-terminal domain to the corresponding diols. Two tyrosine residues (Tyr381 and Tyr465) make up the hydrolase catalytic pocket of the C-terminal hydrolase. These residues connect with the oxygen atom of the epoxide through two hydrogen bonds. On the other side of Tyr381 and Tyr465, the nucleophilic carboxylic acid Asp333 of the catalytic triad Asp333-Asp495-His523 is orientated and activated by His523 and Asp495 and attacks the epoxide carbon backbone to form an ester bond with the opening epoxide. The corresponding diols are produced because of this ester’s subsequent hydrolysis [39,40]. The conversion of EETs to their corresponding diols by sEH diminishes the biological activity of epoxides [8,41].

Several findings showed that urea and amide groups fit well in the hydrolase catalytic pocket [14,42]. Specifically, the carbonyl oxygen of amide or urea is involved in hydrogen bond formation with tyrosine residues and the NH of ureas or amides serves as a hydrogen bond donor to Asp333. Hence, urea and amide derivatives have been developed as competitive, reversible, and highly potent sEH inhibitors [43,44]. Urea derivatives have been created continually due to their high potency, with a focus on improving their solubility and bioavailability to potentially cure EpFA-related disorders. 

The first enhancement to the sEH inhibitor design from DCU was the addition of a flexible chain, such as 12-(3-adamantan-1-ylureido)dodecanoic acid (AUDA), on one side of the urea function. While keeping the inhibitory effect, the addition of a flexible side chain greatly increased AUDA’s water solubility and decreased the melting point (Figure 1B) [42]. The adamantyl group utilized in AUDA produced remarkably high detection sensitivity in an LC–MS analysis as well as high potency in the target sEH in the early stages of sEHi development. 

Our study features the synthesis and evaluation of urea derivatives bearing aryl sulfonyl groups as soluble epoxide hydrolase inhibitors. We initiated our design from the urea core, incorporating key structural requisites for manifesting significant sEH inhibition. Strategic substitution at specific positions through SAR development in the lead led to potent inhibitors on human sEH. AUDA was used as the positive control for the fluorescence assay, against which the inhibitory activity of the compounds (evaluated in terms of the IC_50_ value) was monitored (Table 1). Most of the urea and thiourea compounds were found to have nanomolar to sub micromolar potency against human recombinant sEH. We strategically kept the adamantyl group attached to the urea core as the catalytic site is lipophilic in nature. To increase the polarity and improve the solubility in water, we maintained the substituted aromatic sulfonyl group. To begin with, we changed the aromatic substitution to determine how that plays a role in potency (**4a–l**). Compound **4a** with the phenyl substitution was found to manifest moderate sEH inhibition, with an IC_50_ value of 0.10 µM against human recombinant sEH (Table 2). The incorporation of a methyl group at the *para* position of the phenyl group markedly enhanced potency in compound **4b** (IC_50_ 16.3 nM). More, bulkier groups at the *para* position resulted in a drastic drop in potency (**4c–d**). Interestingly, when alkoxy groups such as 4-OMe and 4-OCF_3_ were present there, it showed very significant sEH inhibitory activity, having IC_50_ values of 1.69 and 1.21 nM, respectively (Table 2, Figure S1). But when the position of the -OCF_3_ was altered from *para* to *meta* (**4g**) and *ortho* (**4h**), sEH inhibition decreased to a significant extent (Table 1). In the next set of compounds, we replaced the para substituents with fluoro and chloro groups in compounds **4j** and **4k**. Both were promising sEH inhibitors, the latter being more active with IC_50_ 2 nM. When the substituted phenyl group was replaced with the naphthyl group in compound **4l**, it resulted in a marked enhancement in the potency (IC_50_ 1.21 nM). In our next modifications, we replaced the urea group with a thiourea in compounds **6a–d** and methylated thiourea (**7**). Out of them, **6a** and **6d** were active, having IC_50_ values 0.13 µM and 4.66 nM, respectively. In the next set of compounds (**8a–b** and **9a–d**), we incorporated amine and hydrazine substituents, which led to a marked decrease in the potency for most of the compounds. The only exception was compound **9a**, with an IC_50_ value of 14.2 nM (Table 2). When 1-adamantyl attachment to the urea was substituted with the 3-hydroxy-1-adamantyl group (**10a–c**) and several bicycloamines (**11a–d**), the inhibitory activity diminished, advocating for the fact that the presence of 1-adamantylamine is very important. In a subsequent development, we attached an isomer 2-adamantyl group (**12**) and replaced the 1-adamantyl group with a couple of aliphatic cyclohexyl groups (**13–14, 16–17**) but none of them showed any activity (Table 1). 3,5-Dimethyl-1-adamantyl group replacements in compounds **15a** and **15b** were found to have significant sEH inhibition, with IC_50_ values of 3.87 nM and 0.95 µM, respectively. We synthesized compound **18** with two urea groups in it. It showed sEH inhibition, with an IC_50_ value of 38.7 nM (Figure S1). In a nutshell, we can conclude that the strategic structure–activity relationship development led to a set of modestly potent sEH inhibitors with substituents such as adamantyl and substituted phenyl groups in the urea pharmacophore. In the sulfonyl urea-based sEHi, we found the adamantyl group to be indispensable for sEH inhibition in the sulfonyl ureas series. To our satisfaction, we found that most of the urea compounds exhibited nanomolar to sub micromolar sEH inhibition, with compounds **4f** and **4l** being the most active inhibitors, having IC_50_ values of 2.94 and 1.69 nM, respectively, against human recombinant sEH (Table 2). We then investigated their potency against mouse recombinant sEH. Surprisingly, although somewhat precedented, many compounds that were potent in *h*sEH showed a lower order of potency in mouse recombinant sEH [27,45].

### 3.2. Molecular Docking Study with ** *4f*** and ** *4l***

**Figure 2:**
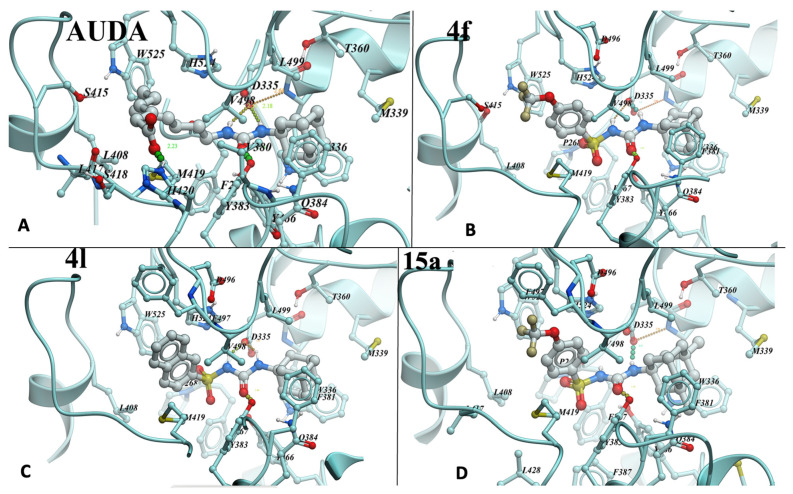
AUDA (**A**), **4f** (**B**), **4l** (**C**), and **15a** (**D**) compounds docked into the active site of human sEH. **4f** and other sulfonyl urea-based sEHi were docked into PDB:6FR2 using ICM-Pro software version 3.9-3b (Molsoft, San Diego, CA, USA) [46,47]. Residues involved in contact with the compounds are numbered.

To evaluate the binding mode of sulfonyl urea-based sEHi, we conducted molecular docking of lead compounds (Figure 2A–D). The structure of sEHi complexed to LK864 (1-[(4~{S})-9-propan-2-ylsulfonyl-1-oxa-9-azaspiro[5.5]undecan-4-yl]-3-[[4-(trifluoromethyloxy)phenyl]methyl]urea (PDB:6FR2) was chosen for modeling because it produced the best prediction of the binding pose of the “native” ligand compared to re-docking for other structures of sEH–urea complexes in PDB. RMSD for the prediction was 0.975 Å. Sulfonyl ureas indeed dock similarly to ureas: nitrogens of the urea group form hydrogen bonds with Asp335 carboxyl, while carbonyl forms a hydrogen bond with the hydroxyl of Tyr383 (Figure 2). Although the sulfonyl group is not involved in hydrogen bond formation, its computed contribution to binding energy per atom in **4f** (4.3%) exceeds that of the urea (3.75%) and (trifluoromethyloxy)phenyl groups (2.2%). Only the adamantyl group contributes more, 5% per atom. Remarkably, adamantyl groups of AUDA, **4f**, and **4l** (Figure 2A–C) overlap completely and fill the corresponding part of the pocket cavity completely, thus explaining the preference for the group in the most active compounds. It is possible that another bulky group may work as well or better during the optimization of inhibitors if it would allow for additional interactions with nearby Q384. Meanwhile, a subpocket occupied by the (trifluoromethyloxy)phenyl group of **4f** or the naphthyl moiety of **4l** is not filled completely. Thus, the affinity and solubility of the derivatives can be further improved by hydrogen bonding to the Ser415 sidechain of the pocket and interactions with the main chain of the 415–419 loop. The carboxyl group of AUDA forms an additional hydrogen bond with His420 (Figure 2A), with ionic interaction also possible. However, the high flexibility of AUDA’s long hydrocarbon chain, leading to an entropic penalty, is a likely reason for the observed higher Kd that is improved in more rigid derivatives. Notably, the compounds in this work had a higher potency for human sEH but showed a lower order of potency for mouse sEH. Docking results, however, were unable to pinpoint the species-difference inhibitory potency seen for this set of compounds.

### 3.3. In Vitro ADME and In Vivo Pharmacokinetic Study

Based on the sEH inhibition, we shortlisted compounds **4f** and **4l**, with similar structural features except for their nature in terms of aromatic substitution, to evaluate further. **4f** with 4-trifluoromethoxyphenyl and **4l** with the naphthyl group were evaluated in a panel of in vitro ADME assays [48,49]. They were assessed through a series of established assays, providing us insights into how in vitro pharmacokinetic properties are affected by structural modifications in the scaffold. We investigated aqueous solubility at pH 7.4, Caco-2 permeability, and plasma protein binding, and performed a microsomal stability. 

Assay, through a series of established in vitro experiments (Table 3). Compound **4f** was found to have moderate aqueous solubility as compared to **4l**. This is due to the presence of a more polar -OCF_3_ group in **4f** in comparison to the highly hydrophobic naphthyl group in **4l**. It is noteworthy that both **4f** and **4l** demonstrated good human plasma stability even after 2 h. Caco-2 permeability for these compounds showed that these compounds are brain penetrable, and their efflux ratios (<2) indicated that **4f** and **4l** are not Pgp substrates. 

**Figure 3:**
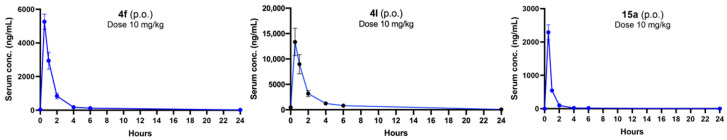
Concentrations of **4f, 4l**, and **15a** in serum after single oral administrations at 10 mg/kg dose.

Next, we proceeded with in vivo investigations using compound **4f** after taking into account all of the previously mentioned parameters as all of the in vitro pharmacokinetic parameters of this compound are in the ideal range. We have explored the in vivo pharmacokinetic profile of compounds **4f**, **4l**, and **15a**. Both the compounds **4f** and **15a** are structurally very similar, only differing in the aliphatic amine attachment. We found that the plasma half-life was short for compounds **4f** and **15a** (<2 h) after both oral and intraperitoneal administration (10 mg/kg) of the compounds in an in vivo pharmacokinetics study (Figure 3), although the C_max_ value of **4f** was found to be better than that of **15a**. Compound **4l** also exhibited a promising C_max_ value after oral administration (10 mg/kg), though the half-life value is similar to compound **4f.** Their intraperitoneal PK profiles were also evaluated (Supplementary Materials; Figure S2). 

### 3.4. In Vivo Therapeutic Efficacy Study

#### 3.4.1. Analgesic Effects of **4f** and **4l** in Pain Models

**Figure 4:**
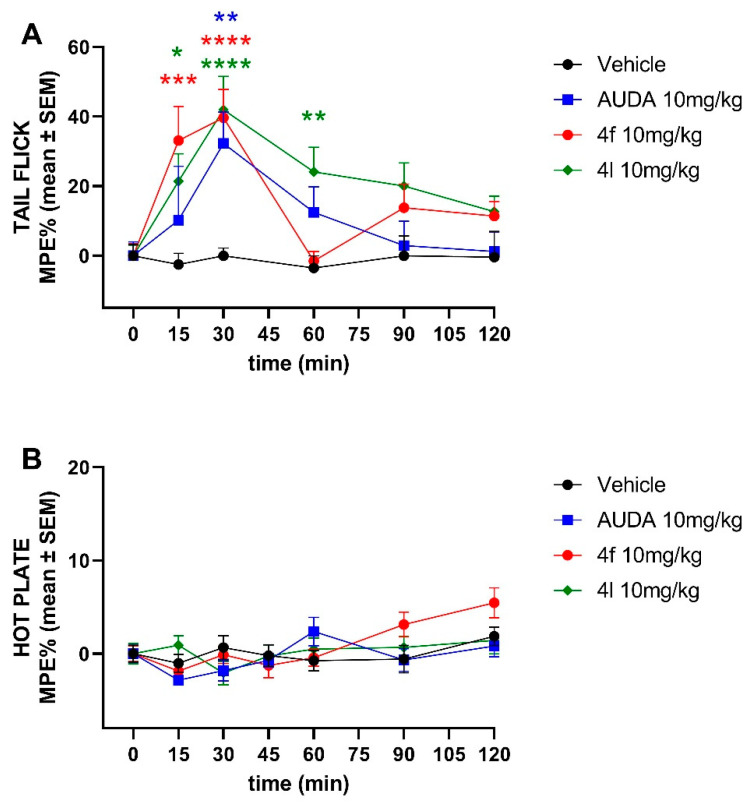
Time-course effects of ip-administered sEH inhibitors in the (**A**) tail-flick and the (**B**) hot plate test. Data are means ± SEM, n = 10–14 mice/group. * *p* < 0.05, ** *p* < 0.01, *** *p* < 0.001, and **** *p* < 0.0001. MPE, maximum possible effect.

sEH inhibitors have been implicated to have analgesic properties. Therefore, we investigated first the analgesic effects of **4f** and **4l** in tail-flick and hot plate pain models. We found that both **4f** and **4l** demonstrate similar analgesic activity to AUDA after a single intraperitoneal injection at 10 mg/kg in a tail-flick model (Figure 4). However, none of the sEH inhibitors, including AUDA, showed any analgesic effect in the hot plate assay, suggesting mechanisms other than CNS-mediated.

#### 3.4.2. Effects of **4f** and **4l** on Lipopolysaccharide (LPS)-Induced Acute Lung Inflammation

**Figure 5:**
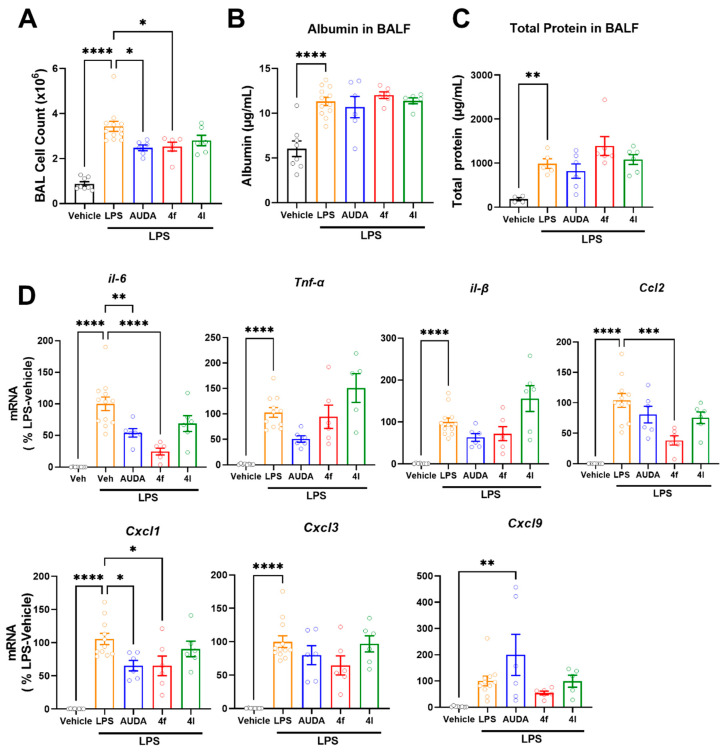
In vivo effects of 4f and 4l treatments in LPS-induced acute lung inflammation. (**A**) Numbers of cells in bronchoalveolar lavage. (**B**) Levels of albumin in bronchoalveolar lavage fluid. (**C**) Levels of total protein in bronchoalveolar lavage fluid. (**D**) Gene expressions of inflammatory cytokines and chemokines in LPS-induced acute lung injury model. Data are means ± SEM, n = 6–12 mice/group. * *p* < 0.05, ** *p* < 0.01, *** *p* < 0.001, and **** *p* < 0.0001. AUDA (10 mg/kg, ip, qd), **4f**, and **4l** (10 mg/kg, ip, qid).

The increased activity of soluble epoxide hydrolase promotes inflammatory conditions. Acute lung injury (ALI) is a state of acute inflammation that results in the disruption of the lung endothelium and epithelial cells [50]. Characteristic features of ALI include the loss of alveolar–capillary membrane integrity, vascular permeability, excessive neutrophil transmigration, and the release of pro-inflammatory mediators and cytotoxic components. LPS causes acute inflammation resulting in an increased number of immune cell infiltrations to the lungs and the expression of cytokines and other inflammatory mediators, eventually leading to mortality in mice. In the present study, an LPS-induced acute lung injury model in mice was used to evaluate the sEH inhibitors for their role in the attenuation of inflammation. LPS increased cell numbers in bronchoalveolar lavage (BAL), indicating an increased infiltration of immune cells (Figure 5A); treatment with either prototypic sEH inhibitor AUDA (10 mg/kg, i.p.) or **4f** (10 mg/kg, i.p.) similarly attenuated the number of cells in BAL (Figure 5A). On the other hand, neither AUDA nor novel sEH inhibitors’ (**4f** and **4l**) effect on LPS induced increases in albumin (Figure 5B) and total protein (Figure 5C) in bronchoalveolar lavage fluid (BALF). Increased albumin levels and total protein levels are indicators of vascular permeability and alveolar–capillary membrane integrity, respectively. Since sEH inhibition by AUDA and **4f** similarly reduced cell infiltration (Figure 5A), this endorsed sEH's role in the inflammatory process. Therefore, we investigated the gene expression of inflammatory markers in lungs (Figure 5D). Interleukin 6, TNF-alpha, and interleukin 1B are the major inflammatory cytokines. Both AUDA and **4f** treatments attenuated LPS-induced expressions of inflammatory cytokines such as interleukin 6 (IL-6) and interleukin 1B (IL-1B) (Figure 5D). However, **4l** had no effect on reducing these. Additionally, both AUDA and **4f** similarly attenuated the expression of *CXCL1* (Figure 5D). Notably, the effect of **4f** was more pronounced in reducing *IL-6* and *CCL-2* expressions (Figure 5D). These results suggest that sEH inhibition has no direct effect on preventing vascular permeability (Figure 5B) and alveolar–capillary membrane integrity (Figure 5C) in LPS-induced acute lung injury. Importantly, these results demonstrate that **4f** achieved comparable and potent in vivo sEH inhibition to that of AUDA in the lungs.

## 4. Experimental Section

### 4.1. Chemical Synthesis and General Methods

All starting materials, reagents, and solvents were purchased from commercial suppliers and used without further purification. Air-sensitive reactions were carried out under a dry nitrogen or argon atmosphere. RediSepRf silica gel columns were used for column chromatographic purification on the Teledyne ISCO CombiFlash system. ^1^H NMR spectra were recorded at 400 MHz (Jeol) and 800 MHz (Bruker) frequencies, and ^13^C NMR spectra were recorded at 100 MHz (Jeol) and 200 MHz (Bruker) frequencies in CDCl_3_ or DMSO-d_6_ solvent using tetramethylsilane (TMS) as the internal standard. Mass spectra (HRMS) were recorded on a VG 7070E spectrometer or a JEOL SX102a mass spectrometer. Melting points were determined on an MPA100 Stanford Research System apparatus. Thin-layer chromatography (TLC) analyses were carried out on Analtech/Miles Scientific silica gel GHLF 0.25 mm plates using various gradients of EtOAc/n-hexanes or CHCl_3_/MeOH. TLC plates were visualized under UV light at 254 nm wavelengths. All compounds tested had ≥95% purity, which was determined by a combination of TLC, ^1^H NMR, ^13^C NMR, liquid chromatography−mass spectrometry (LC−MS), and high-resolution mass spectrometry. Detection in LC−MS was carried out on an Agilent 1200 system using an Agilent EC-C18, Poroshell, 2.7 μM (3 mm × 50 mm) where the mobile phase was 50–98% acetonitrile (0.1% formic acid). The LC−MS chromatogram showed the expected molecular (MH^+^) ions as well as a single peak at UV (254 nm).

**General procedure A for the synthesis of sulfonyl carbamates (3a–l)** [35,51]: synthesis of methyl tosylcarbamate (**3a**): 10 g benzenesulfonamide (**1a**) was dissolved in dry ACN (60 mL). Triethylamine (22 mL, 2.5 equiv.) was added to the solution. Methyl chloroformate **2** (7.4 mL, 1.5 equiv.) was added dropwise to the reaction mixture in an ice-cold condition. The reaction mixture was stirred at room temperature for 12 h. Completion of the reaction was determined by thin-layer chromatography. ACN was removed under a vacuum. The residue was then dissolved in ethyl acetate and the organic layer was extracted with a saturated sodium bicarbonate solution. The aqueous part was acidified with conc. HCl to obtain a precipitate, which was filtered, dried, and concentrated to give compound **3a** as a white solid (9.20 g, 67%). ^1^H NMR (400 MHz; CDCl_3_): *δ* 7.85 (d, *J* = 8.0 Hz, 2H), 7.52 (d, *J* = 8.0 Hz, 2H), 3.72 (s, 3H), 1.87 (s, 3H).

**General procedure B for the synthesis of adamantyl ureas (4a–l):** 5.0 g suitable sulfonyl carbamate (**3**) was dissolved in 50 mL toluene. To the solution, adamantyl amine (0.8 equiv.) was added and the reaction mixture was refluxed for 4 h. Toluene was evaporated, and to it, isopropanol was added and triturated to produce a white solid product (**4a–l**). 

**Synthesis of *N*-(adamantan-1-yl)carbamoyl)benzenesulfonamide (4a):** Reaction of compound **3a** (5 g, 23.23 mmol) and 1-adamantylamine (2.81 g, 18.59 mmol) was done following general procedure B to get compound **4a** (4.12 g, 53%). Mp 179–181 °C; ^1^H NMR (400 MHz; CDCl_3_): *δ* 7.90 (d, *J* = 8.0 Hz, 2H), 7.64 (d, *J* = 8.0 Hz, 1H), 7.55 (t, *J* = 8.0 Hz, 2H), 6.35 (s, 1H), 2.06 (s, 3H), 1.91 (s, 6H), 1.65 (s, 6H). ^13^C NMR (100 MHz; CDCl_3_): *δ* 149.4, 139.7, 133.8, 129.4, 127.0, 52.2, 41.6, 36.2, 29.4. LRMS 619.5, HRMS (C_17_H_23_N_2_O_3_S) [M + H]^+^ found *m*/*z* 357.1243, and calculated 357.1249.

**Synthesis of *N*-((adamantan-1-yl)carbamoyl)-4-methylbenzenesulfonamide (4b):** Reaction of compound **3b** (5 g, 21.81 mmol) and 1-adamantylamine (2.64 g, 17.45 mmol) was performed using general procedure B to afford compound **4b** (3.95 g, 52%). Mp 174–176 °C; ^1^H NMR (800 MHz; CDCl_3_): *δ* 7.74 (d, *J* = 8.0 Hz, 2H), 7.32 (d, *J* = 8.0 Hz, 2H), 6.33 (s, 1H), 2.42 (s, 3H), 2.04 (s, 3H), 1.88 (s, 6H), 1.63 (s, 6H). ^13^C NMR (200 MHz; CDCl_3_): *δ* 149.4, 145.0, 136.9, 130.1, 127.2, 52.2, 41.8, 36.4, 29.6, 21.9. HRMS (C_18_H_25_N_2_O_3_S) [M + H]^+^ found *m*/*z* 349.1582, and calculated 349.1586.

**Synthesis of *N*-((adamantan-1-yl)carbamoyl)-4-(trifluoromethyl)benzenesulfonamide (4c):** Reaction of compound **3c** (5 g, 17.65 mmol) and 1-adamantylamine (2.14 g, 14.12 mmol) was done following general procedure B to afford compound **4c** (4.85 g, 68%). Mp 139–141 °C; ^1^H NMR (400 MHz; CDCl_3_ + CD_3_OD): *δ* 8.05 (d, *J* = 8.0 Hz, 2H), 7.74 (d, *J* = 8.0 Hz, 2H), 2.03 (s, 3H), 1.87 (s, 6H), 1.63 (s, 6H). ^13^C NMR (100 MHz; CDCl_3_ + CD_3_OD): *δ* 127.4, 127.2, 125.8, 125.7, 53.5, 51.1, 41.7, 36.3, 29.4. HRMS (C_18_H_22_F_3_N_2_O_3_S) [M + H]^+^ found *m*/*z* 403.1307, and calculated 403.1303.

**Synthesis of *N*-(adamantan-1-yl)carbamoyl)-4-(*tert*-butyl)benzenesulfonamide (4d):** Reaction of compound **3d** (5 g, 18.43 mmol) and 1-adamantylamine (2.23 g, 14.74 mmol) was performed using general procedure B to get compound **4d** (4.78 g, 66%). Mp 247–249 °C; ^1^H NMR (800 MHz; CDCl_3_): *δ* 7.82 (d, *J* = 8.0 Hz, 2H), 7.55 (d, *J* = 8.0 Hz, 2H), 2.05 (s, 3H), 1.90 (s, 6H), 1.65 (s, 6H), 1.35 (s, 9H). ^13^C NMR (200 MHz; CDCl_3_): *δ* 157.6, 150.3, 137.2, 127.0, 126.4, 52.1, 41.8, 36.4, 31.3, 29.6. HRMS (C_21_H_31_N_2_O_3_S) [M + H]^+^ found *m*/*z* 391.2061, and calculated 391.2055.

**Synthesis of *N*-(adamantan-1-yl)carbamoyl)-4-methoxybenzenesulfonamide (4e):** Reaction of compound **3e** (5 g, 20.39 mmol) and 1-adamantylamine (2.47 g, 16.31 mmol) was done following general procedure B to afford compound **4e** (4.27 g, 57%). Mp 163–165 °C; ^1^H NMR (800 MHz; CDCl_3_): *δ* 7.82 (d, *J* = 8.0 Hz, 2H), 7.01 (d, *J* = 8.0 Hz, 2H), 6.33 (s, 1H), 3.89 (s, 3H), 2.07 (s, 3H), 1.91 (s, 6H), 1.66 (s, 6H). ^13^C NMR (200 MHz; CDCl_3_): *δ* 163.9, 149.5, 131.3, 129.4, 114.6, 55.9, 52.2, 41.8, 36.4, 29.5. HRMS (C_18_H_25_N_2_O_4_S) [M + H]^+^ found *m*/*z* 365.1528, and calculated 365.1535.

**Synthesis of *N*-(adamantan-1-yl)carbamoyl)-4-(trifluoromethoxy)benzenesulfonamide (4f):** Reaction of compound **3f** (5 g, 16.71 mmol) and 1-adamantylamine (2.02 g, 13.37 mmol) was performed following general procedure B to get compound **4f** (4.71 g, 67%). Mp 165–167 °C; ^1^H NMR (400 MHz; CDCl_3_): *δ* 7.96 (d, *J* = 8.0 Hz, 2H), 7.37 (d, *J* = 8.0 Hz, 2H), 2.07 (s, 6H), 1.91 (s, 3H), 1.67 (s, 6H). ^13^C NMR (200 MHz; CDCl_3_): *δ* 153.0, 149.8, 138.1, 129.5, 121.3, 119.0, 52.5, 41.8, 36.3, 29.5. HRMS (C_18_H_22_F_3_N_2_O_4_S) [M + H]^+^ found *m*/*z* 419.1250, and calculated 419.1252.

**Synthesis of *N*-((adamantan-1-yl)carbamoyl)-3-(trifluoromethoxy)benzenesulfonamide (4g):** Reaction of compound **3g** (5 g, 16.71 mmol) and 1-adamantylamine (2.02 g, 13.37 mmol) was performed following general procedure B to get compound **4g** (4.51 g, 65%). Mp 151–153 °C; ^1^H NMR (400 MHz; CDCl_3_): *δ* 7.84 (d, *J* = 8.0 Hz, 1H), 7.75 (s, 1H), 7.60 (t, *J* = 8.0 Hz, 1H), 7.49 (d, *J* = 8.0 Hz, 1H), 6.34 (s, 1H), 2.08 (s, 3H), 1.91 (s, 6H), 1.66 (t, *J* = 12.0 Hz, 6H). ^13^C NMR (100 MHz; CDCl_3_): *δ* 149.4, 131.2, 126.1, 125.3, 119.7, 52.4, 41.6, 36.2, 29.4. HRMS (C_18_H_22_F_3_N_2_O_4_S) [M + H]^+^ found *m*/*z* 419.1252, and calculated 419.1252.

**Synthesis of *N*-((adamantan-1-yl)carbamoyl)-2-(trifluoromethoxy)benzenesulfonamide (4h):** Reaction of compound **3h** (5 g, 16.71 mmol) and 1-adamantylamine (2.02 g, 13.37 mmol) was done following general procedure B to afford compound **4h** (4.39 g, 63%). Mp 170–172 °C; ^1^H NMR (400 MHz; CDCl_3_): *δ* 8.01 (d, *J* = 8.0 Hz, 1H), 7.69 (t, *J* = 8.0 Hz, 1H), 7.46–7.42 (m, 2H), 6.16 (s, 1H), 2.02 (s, 3H), 1.83 (s, 6H), 1.61 (t, *J* = 12.0 Hz, 6H). ^13^C NMR (100 MHz; CDCl_3_): *δ* 148.4, 135.6, 131.4, 130.8, 126.6, 120.6, 52.2, 41.5, 36.2, 29.4. HRMS (C_18_H_22_F_3_N_2_O_4_S) [M + H]^+^ found *m*/*z* 419.1260, and calculated 419.1252.

**Synthesis of *N*-((adamantan-1-yl)carbamoyl)-4-cyanobenzenesulfonamide (4i):** Reaction of compound **3i** (5 g, 20.81 mmol) and 1-adamantylamine (2.52 g, 16.65 mmol) was done following general procedure B to get compound **4i** (3.95 g, 53%). Mp 194–196 °C; ^1^H NMR (400 MHz; CDCl_3_): *δ* 8.02 (d, *J* = 8.0 Hz, 2H), 7.85 (d, *J* = 8.0 Hz, 1H), 6.20 (s, 1H), 2.07 (s, 2H), 1.89 (s, 1H), 1.67 (t, *J* = 12.0 Hz, 6H). ^13^C NMR (200 MHz; CDCl_3_): *δ* 149.2, 143.9, 133.3, 127.9, 117.7, 117.2, 52.7, 41.8, 36.3, 29.5. HRMS (C_18_H_22_N_3_O_3_S) [M + H]^+^ found *m*/*z* 360.1376, and calculated 360.1382.

**Synthesis of *N*-(adamantan-1-yl)carbamoyl)-4-fluorobenzenesulfonamide (4j):** Reaction of compound **3j** (5 g, 21.44 mmol) and 1-adamantylamine (2.59 g, 17.15 mmol) was done following general procedure B to get compound **4j** (4.12 g, 54%). Mp 169–171 °C; ^1^H NMR (400 MHz; CDCl_3_): *δ* 7.90 (d, *J* = 8.0 Hz, 2H), 7.20 (d, *J* = 8.0 Hz, 2H), 6.27 (s, 1H), 2.06 (s, 3H), 1.90 (s, 6H), 1.65 (t, *J* = 12.0 Hz, 6H). ^13^C NMR (100 MHz; CDCl_3_): *δ* 164.5, 149.3, 135.7, 129.9, 116.8, 52.3, 41.7, 36.2, 29.4. HRMS (C_17_H_22_N_2_O_3_FS) [M + H]^+^ found *m*/*z* 353.1336, and calculated 353.1335.

**Synthesis of *N*-(adamantan-1-yl)carbamoyl)-4-chlorobenzenesulfonamide (4k):** Reaction of compound **3k** (5 g, 20.03 mmol) and 1-adamantylamine (2.42 g, 16.02 mmol) was performed following general procedure B to afford compound **4k** (4.34 g, 59%). Mp 163–165 °C; ^1^H NMR (800 MHz; CDCl_3_): *δ* 7.81 (d, *J* = 8.0 Hz, 2H), 7.51 (d, *J* = 8.0 Hz, 2H), 6.25 (s, 1H), 2.05 (s, 3H), 1.88 (s, 6H), 1.63 (s, 6H). ^13^C NMR (200 MHz; CDCl_3_): *δ* 149.4, 140.6, 138.3, 129.8, 128.6, 52.5, 41.8, 36.3, 29.5. HRMS (C_17_H_22_ClN_2_O_3_S) [M + H]^+^ found *m*/*z* 369.1037, and calculated 369.1040.

**Synthesis of *N*-(adamantan-1-yl)carbamoyl)naphthalene-2-sulfonamide (4l):** Reaction of compound **3l** (5 g, 18.85 mmol) and 1-adamantylamine (2.28 g, 15.08 mmol) was done following general procedure B to get compound **4l** (4.13 g, 57%). Mp 155–157 °C; ^1^H NMR (400 MHz; CDCl_3_): *δ* 8.44 (s, 1H), 7.95–7.88 (m, 3H), 7.83 (d, *J* = 8.0 Hz, 1H), 7.68–7.59 (m, 2H), 6.39 (s, 1H), 2.02 (s, 6H), 1.93 (s, 3H), 1.89 (s, 6H). ^13^C NMR (100 MHz; CDCl_3_): *δ* 149.6, 136.6, 135.3, 132.0, 129.8, 129.4, 128.7, 128.1, 127.9, 121.9, 52.2, 41.7, 36.2, 29.4. HRMS (C_21_H_25_N_2_O_3_S) [M + H]^+^ found *m*/*z* 385.1582, and calculated 385.1586.

**General procedure C for the synthesis of adamantyl thioureas (6a–d):** Compound **4** (0.5 g, 1.0 equiv.) was dissolved in dry toluene (8 mL). To the solution was added N, N-diisopropylethylamine (2.5 equiv.) under a N_2_ atmosphere. POCl_3_ (2 equiv.) was added to the reaction mixture under an ice-cold condition, and it was refluxed for 1 h. Completion of the reaction was confirmed by thin-layer chromatography to yield intermediates **5a–d**, which were proceeded to the next step. Toluene was evaporated and a solution of sodium thiosulfate (2 equiv.) dissolved in a 9 mL mixture of dioxane and water (8:1) was added dropwise to the reaction mixture. The reaction mixture was heated at 85–90 °C for 1–2 h. The solvent was removed under a vacuum, extracted with DCM, and washed with a brine solution. The residue was purified by silica gel column chromatography to provide compounds **6a–d** as white solids.

**Synthesis of *N*-((adamantan-1-yl)carbamothioyl)benzenesulfonamide (6a):** Compound **4a** (0.5 g, 1.50 mmol) was reacted with POCl_3_ (0.28 mL, 3.0 mmol) in dry toluene in the presence of N, N-diisopropylethylamine (0.65 mL, 3.75 mmol) as a base. Solvent was evaporated and the intermediate **5a** formed was reacted with sodium thiosulfate (0.47 g, 3.0 mmol) according to general procedure C to form compound **6a** (0.36 g, 69%). Mp 144–146 °C; ^1^H NMR (800 MHz; CDCl_3_): *δ* 7.92 (s, 1H), 7.89 (d, *J* = 8.0 Hz, 2H), 7.69 (d, *J* = 8.0 Hz, 1H), 7.59 (t, *J* = 8.0 Hz, 2H), 2.12 (s, 6H), 2.10 (s, 3H), 1.67 (s, 6H). ^13^C NMR (200 MHz; CDCl_3_): *δ* 175.2, 138.6, 134.4, 129.7, 127.3, 55.9, 40.6, 36.3, 29.6. HRMS (C_17_H_23_N_2_O_2_S_2_) [M + H]^+^ found *m*/*z* 351.1199, and calculated 351.1201. 

**Synthesis of *N*-((adamantan-1-yl)carbamothioyl)-4-(*tert*-butyl)benzenesulfonamide (6b):** Compound **4d** (0.5 g, 1.28 mmol) was reacted with POCl_3_ (0.24 mL, 2.56 mmol) in dry toluene in the presence of N, N-diisopropylethylamine (0.56 mL, 3.2 mmol) as a base. Solvent was evaporated and the intermediate **5b** formed was reacted with sodium thiosulfate (0.40 g, 2.56 mmol) according to general procedure C to form compound **6b** (0.39 g, 75%). Mp 171–173 °C; ^1^H NMR (800 MHz; CDCl_3_): *δ* 7.93 (s, 1H), 7.79 (d, *J* = 8.0 Hz, 2H), 7.58 (d, *J* = 8.0 Hz, 2H), 2.11 (s, 6H), 2.09 (s, 3H), 1.66 (s, 6H), 1.35 (s, 9H). ^13^C NMR (200 MHz; CDCl_3_): *δ* 175.4, 158.6, 135.6, 127.2, 126.7, 55.9, 40.5, 36.3, 31.2, 29.6. HRMS (C_21_H_31_N_2_O_2_S_2_) [M + H]^+^ found *m*/*z* 407.1833, and calculated 407.1827.

**Synthesis of *N*-((adamantan-1-yl)carbamothioyl)-4-methoxybenzenesulfonamide (6c):** Compound **4e** (0.5 g, 1.37 mmol) was reacted with POCl_3_ (0.26 mL, 2.74 mmol) in dry toluene in the presence of N, N-diisopropylethylamine (0.60 mL, 3.4 mmol) as a base. Solvent was evaporated and the intermediate **5c** formed was reacted with sodium thiosulfate (0.43 g, 2.74 mmol) according to general procedure C to form compound **6c** (0.32 g, 61%). Mp 172–174 °C; ^1^H NMR (800 MHz; CDCl_3_): *δ* 7.93 (s, 1H), 7.81 (d, *J* = 8.0 Hz, 2H), 7.03 (d, *J* = 8.0 Hz, 2H), 3.90 (s, 3H), 2.14 (s, 6H), 2.10 (s, 3H), 1.67 (s, 6H). ^13^C NMR (200 MHz; CDCl_3_): *δ* 175.5, 164.3, 130.0, 129.6, 114.9, 56.0, 55.9, 40.6, 36.4, 29.6. HRMS (C_18_H_25_N_2_O_3_S_2_) [M + H]^+^ found *m*/*z* 381.1314, and calculated 381.1307. 

**Synthesis of N-((adamantan-1-yl)carbamothioyl)-4-(trifluoromethoxy)benzenesulfonamide (6d):** Compound **4f** (0.5 g, 1.19 mmol) was reacted with POCl_3_ (0.22 mL, 2.38 mmol) in dry toluene in the presence of N, N-diisopropylethylamine (0.52 mL, 3.0 mmol) as a base. Solvent was evaporated and the intermediate **5d** formed was reacted with sodium thiosulfate (0.38 g, 2.38 mmol) according to general procedure C to form compound **6d** (0.41 g, 79%). Mp 152–154 °C; ^1^H NMR (800 MHz; CDCl_3_): *δ* 7.91 (d, *J* = 8.0 Hz, 1H), 7.84 (s, 1H), 7.39 (d, *J* = 8.0 Hz, 1H), 2.09 (s, 6H), 1.92 (s, 3H), 1.64 (t, *J* = 16.0 Hz, 6H). ^13^C NMR (200 MHz; CDCl_3_): *δ* 175.1, 153.4, 136.8, 129.6, 128.4, 121.3, 42.8, 40.6, 36.3, 29.6. HRMS C_18_H_22_F_3_N_2_O_3_S_2_ [M + H]^+^ found *m*/*z* 435.1029, and calculated 435.1024.

**Synthesis of methyl N-((adamantan-1-yl)-N′-((4-(trifluoromethoxy)phenyl)sulfonyl)carbamimidothioate (7):** Compound **4d** (0.5 g, 1.28 mmol) was reacted with POCl_3_ (0.24 mL, 2.56 mmol) in dry toluene in the presence of N, N-diisopropylethylamine (0.56 mL, 3.2 mmol) as a base. The toluene was evaporated and the intermediate **5b** formed was reacted with a solution of sodium thiosulfate (0.40 g, 2.56 mmol) in a 9 mL mixture of dioxane and water (8:1). The reaction mixture was heated at 85–90 °C for 1–2 h. The solvent was removed under a vacuum, excess methyl iodide was added to it, and the reaction mixture was stirred at room temperature for 15 min. The solvent was removed under a vacuum, extracted with DCM, and washed with a brine solution. The residue was purified by silica gel column chromatography to afford compound **7** (0.33 g, 61%). Mp 121–123 °C; ^1^H NMR (800 MHz; CDCl_3_): *δ* 7.92 (d, *J* = 8.0 Hz, 1H), 7.28 (d, *J* = 8.0 Hz, 1H), 2.36 (s, 3H), 2.10 (s, 3H), 2.01 (s, 6H), 1.64 (t, *J* = 16.0 Hz, 6H). ^13^C NMR (200 MHz; CDCl_3_): *δ* 151.8, 141.5, 128.9, 128.5, 121.3, 120.9, 41.9, 36.2, 29.7, 15.3. HRMS (C_19_H_24_F_3_N_2_O_3_S_2_) [M + H]^+^ found *m*/*z* 449.1180, and calculated 449.1180.

**General procedure D for the synthesis of compounds 8a–b and 9a–d:** Compound **4f** (0.5 g, 1.0 equiv.) was dissolved in dry toluene (8 mL). To the solution was added N, N-diisopropylethylamine (2.5 equiv.) under a N_2_ atmosphere. POCl_3_ (2 equiv.) was added to the reaction mixture under an ice-cold condition, and it was refluxed for 1 h. Completion of the reaction was confirmed by thin-layer chromatography. Intermediate **5f** was not isolated. To the reaction mixture dissolved in DCM were added various amine and hydrazine derivatives (2 equiv.). The solvent was removed under a vacuum, extracted with DCM, and washed with a brine solution. The residue was purified by silica gel column chromatography to afford compounds **8a–b** and **9a–d** as solids.

**Synthesis of *N*-((adamantan-1-yl)amino)(amino)methylene)-4-(trifluoromethoxy)benzenesulfonamide (8a):** Compound **4f** (0.5 g, 1.19 mmol) was reacted with POCl_3_ (0.22 mL, 2.38 mmol) in dry toluene in the presence of N, N-diisopropylethylamine (0.52 mL, 3.0 mmol) as a base. Solvent was evaporated and the intermediate **5d** formed was reacted with 1 mL ammonia in a methanol solution (7M) according to general procedure D to form compound **8a** (0.31 g, 62%). Mp 221–223 °C; ^1^H NMR (400 MHz; CDCl_3_): *δ* 7.93 (d, *J* = 8.0 Hz, 2H), 7.28 (d, *J* = 8.0 Hz, 2H), 5.99 (s, 2H), 2.10 (s, 3H), 1.92 (s, 6H), 1.66 (t, *J* = 12.0 Hz, 6H). ^13^C NMR (200 MHz; CDCl_3_): *δ* 155.7, 146.1, 142.3, 131.5, 128.0, 120.8, 52.9, 42.1, 36.1, 29.4, 14.1. HRMS (C_18_H_23_F_3_N_3_O_3_S) [M + H]^+^ found *m*/*z* 418.1409, and calculated 418.1412.

**Synthesis of *N*-((adamantan-1-yl)amino)(methylamino)methylene)-4-(trifluoromethoxy)benzenesulfonamide (8b):** Compound **4f** (0.5 g, 1.19 mmol) was reacted with POCl_3_ (0.22 mL, 2.38 mmol) in dry toluene in the presence of N, N-diisopropylethylamine (0.52 mL, 3.0 mmol) as a base. Solvent was evaporated and the intermediate **5d** formed was reacted with methylamine hydrochloride (0.16 g, 2.38 mmol) according to general procedure D to afford compound **8b** (0.35 g, 67%). Mp 134–136 °C; ^1^H NMR (400 MHz; CDCl_3_): *δ* 7.92 (d, *J* = 8.0 Hz, 2H), 7.26 (d, *J* = 8.0 Hz, 2H), 2.79 (s, 3H), 2.07 (s, 3H), 1.94 (s, 2H), 1.64 (t, *J* = 16.0 Hz, 6H). ^13^C NMR (100 MHz; CDCl_3_): *δ* 155.1, 151.1, 142.8, 128.0, 120.7, 42.3, 36.1, 29.5, 28.4. HRMS (C_19_H_25_F_3_N_3_O_3_S) [M + H]^+^ found *m*/*z* 432.1565, and calculated 432.1569.

**Synthesis of *N*-((adamantan-1-yl)-N′-((4-(trifluoromethoxy)phenyl)sulfonyl)hydrazinecarboximidamide (9a):** Compound **4f** (0.5 g, 1.19 mmol) was reacted with POCl_3_ (0.22 mL, 2.38 mmol) in dry toluene in the presence of N, N-diisopropylethylamine (0.52 mL, 3.0 mmol) as a base. Solvent was evaporated and the intermediate **5d** formed was reacted with 1 mL solution of hydrazine monohydrate according to general procedure D to afford compound **9a** (0.40 g, 77%). Mp 134–136 °C; ^1^H NMR (400 MHz; CDCl_3_): *δ* 8.11 (s, 1H), 7.92 (d, *J* = 8.0 Hz, 2H), 7.26 (d, *J* = 8.0 Hz, 2H), 6.44 (s, 1H), 3.64 (s, 2H), 2.05 (s, 3H), 1.99 (s, 6H), 1.64 (t, *J* = 12.0 Hz, 6H). ^13^C NMR (100 MHz; CDCl_3_): *δ* 155.8, 142.7, 127.9, 120.7, 52.4, 42.0, 36.3, 29.5. HRMS C_18_H_24_F_3_N_4_O_3_S [M + H]^+^ found *m*/*z* 433.1517, and calculated 433.1521.

**Synthesis of *N*-((adamantan-1-yl)-2-methyl-N′-((4-(trifluoromethoxy)phenyl)sulfonyl)hydrazine-1-carboximidamide (9b):** Compound **4f** (0.5 g, 1.19 mmol) was reacted with POCl_3_ (0.22 mL, 2.38 mmol) in dry toluene in the presence of N, N-diisopropylethylamine (0.52 mL, 3.0 mmol) as a base. Solvent was evaporated and the intermediate **5d** formed was reacted with methylhydrazine (0.12 mL, 2.38 mmol) according to general procedure D to afford compound **9b** (0.31 g, 58%). Mp 212–214 °C; ^1^H NMR (400 MHz; CDCl_3_): *δ* 7.95 (d, *J* = 8.0 Hz, 2H), 7.27 (d, *J* = 8.0 Hz, 2H), 6.66 (s, 1H), 3.57 (s, 3H), 1.81 (s, 3H), 1.67 (s, 6H), 1.48 (d, *J* = 12.0 Hz, 3H), 1.28 (d, *J* = 12.0 Hz, 3H). ^13^C NMR (100 MHz; CDCl_3_): *δ* 155.4, 143.5, 127.8, 126.7, 120.8, 52.7, 45.2, 41.8, 36.0, 29.3. HRMS C_19_H_26_F_3_N_4_O_3_S [M + H]^+^ found *m*/*z* 447.1684, and calculated 447.1678.

**Synthesis of *N*-((adamantan-1-yl)-2-phenyl-N′-((4-(trifluoromethoxy)phenyl)sulfonyl)hydrazine-1-carboximidamide (9c):** Compound **4f** (0.5 g, 1.19 mmol) was reacted with POCl_3_ (0.22 mL, 2.38 mmol) in dry toluene in the presence of N, N-diisopropylethylamine (0.52 mL, 3.0 mmol) as a base. Solvent was evaporated and the intermediate **5d** formed was reacted with phenylhydrazine (0.23 mL, 2.38 mmol) according to general procedure D to afford compound **9c** (0.31 g, 62%). Mp 220–222 °C; ^1^H NMR (400 MHz; CDCl_3_): *δ* 8.46 (s, 1H), 7.97 (d, *J* = 8.0 Hz, 2H), 7.29 (d, *J* = 8.0 Hz, 2H), 7.22 (d, *J* = 8.0 Hz, 2H), 6.99 (d, *J* = 8.0 Hz, 1H), 6.70 (d, *J* = 8.0 Hz, 2H), 6.10 (s, 1H), 5.63 (s, 1H), 2.04 (s, 3H), 1.98 (s, 6H), 1.63 (t, *J* = 12.0 Hz, 6H). ^13^C NMR (100 MHz; CDCl_3_): *δ* 155.2, 146.0, 142.5, 129.7, 128.2, 122.7, 120.8, 113.8, 52.9, 41.8, 36.2, 29.5. HRMS C_24_H_28_F_3_N_4_O_3_S [M + H]^+^ found *m*/*z* 509.1841, and calculated 509.1834.

**Synthesis of *N*-((adamantan-1-yl)-2,2-dimethyl-N′-((4-(trifluoromethoxy)phenyl)sulfonyl)hydrazine-1-carboximidamide (9d):** Compound **4f** (0.5 g, 1.19 mmol) was reacted with POCl_3_ (0.22 mL, 2.38 mmol) in dry toluene in the presence of N, N-diisopropylethylamine (0.52 mL, 3.0 mmol) as a base. Solvent was evaporated and the intermediate **5d** formed was reacted with N,N-dimethylhydrazine (0.18 mL, 2.38 mmol) according to general procedure D to afford compound **9d** (0.33 g, 60%). Mp 126–128 °C; ^1^H NMR (400 MHz; CDCl_3_): *δ* 7.92 (d, *J* = 8.0 Hz, 2H), 7.65 (s, 1H), 7.26 (d, *J* = 8.0 Hz, 2H), 6.27 (s, 1H), 2.49 (s, 6H), 2.04 (s, 3H), 1.96 (s, 6H), 1.63 (t, *J* = 12.0 Hz, 6H). ^13^C NMR (100 MHz; CDCl_3_): *δ* 153.4, 151.1, 142.8, 127.9, 120.6, 52.4, 47.6, 42.0, 36.3, 29.5. HRMS C_20_H_28_F_3_N_4_O_3_S [M + H]^+^ found *m*/*z* 461.1840, and calculated 461.1834.

**General procedure E for the synthesis of compounds 10a–c:** 5.0 g suitable sulfonyl carbamates (**3b–d**) was dissolved in 50 mL toluene. To the solution, 3-aminoadamantan-1-ol (0.8 equiv.) was added, and the reaction mixture was refluxed for 4 h. Toluene was evaporated, and to it, isopropanol was added and triturated to produce a white solid product (**10a–c**). 

**Synthesis of *N*-((3-hydroxyadamantan-1-yl)carbamoyl)-4-methylbenzenesulfonamide (9a):** Compound **3b** (5.0 g, 21.81 mmol) was dissolved in 50 mL toluene. 3-aminoadamantan-1-ol (2.92 g, 17.45 mmol) was added to the solution, and the reaction was performed according to general procedure E to get compound **10a** (4.36 g, 55%). Mp 192–194 °C; ^1^H NMR (400 MHz; CDCl_3_ + CD_3_OD): *δ* 7.80 (d, *J* = 8.0 Hz, 2H), 7.34 (d, *J* = 8.0 Hz, 2H), 2.45 (s, 3H), 2.23 (s, 2H), 2.06 (s, 1H), 1.84 (s, 2H), 1.79 (s, 4H), 1.52 (s, 2H), 1.26 (s, 4H). ^13^C NMR (100 MHz; CDCl_3_ + CD_3_OD): *δ* 150.1, 144.5, 136.9, 129.8, 127.1, 68.7, 60.6, 53.9, 48.6, 43.6, 40.3, 34.7, 30.5, 21.6. HRMS C_18_H_25_N_2_O_4_S [M + H]^+^ found *m*/*z* 365.1538, and calculated 365.1535.

**Synthesis of *N*-((-3-hydroxyadamantan-1-yl)carbamoyl)-4-(trifluoromethyl)benzenesulfonamide (10b):** Compound **3c** (5.0 g, 17.65 mmol) was dissolved in 50 mL toluene. 3-aminoadamantan-1-ol (2.36 g, 14.12 mmol) was added to the solution and the reaction was performed according to general procedure E to provide compound **10b** (3.81 g, 52%). Mp 164–166 °C; ^1^H NMR (400 MHz; CDCl_3_ + CD_3_OD): *δ* 8.12 (d, *J* = 8.0 Hz, 1H), 7.83 (d, *J* = 8.0 Hz, 2H), 2.22 (s, 2H), 1.83 (s, 2H), 1.80 (s, 4H), 1.64 (s, 4H), 1.52 (s, 2H). ^13^C NMR (100 MHz; CDCl_3_ + CD_3_OD): *δ* 136.2, 128.4, 126.4, 122.0, 90.3, 68.8, 54.2, 43.8, 40.5, 35.0, 30.8. HRMS C_18_H_22_F_3_N_2_O_4_S [M + Na]^+^ found *m*/*z* 441.1074, and calculated 441.1072.

**Synthesis of 4-(*tert*-butyl)-*N*-((-3-hydroxyadamantan-1-yl)carbamoyl)benzenesulfonamide (10c):** Compound **3d** (5.0 g, 18.43 mmol) was dissolved in 50 mL toluene. 3-aminoadamantan-1-ol (2.47 g, 14.74 mmol) was added to the solution, and the reaction was performed according to general procedure E to afford compound **10c** (3.95 g, 53%). Mp 187–189 °C; ^1^H NMR (400 MHz; CDCl_3_): *δ* 7.76 (d, *J* = 8.0 Hz, 2H), 7.47 (d, *J* = 8.0 Hz, 2H), 3.13 (s, 6H), 2.15 (s, 2H), 1.78 (s, 2H), 1.71 (t, *J* = 12.0 Hz, 3H), 1.44 (s, 2H), 1.27 (s, 9H). ^13^C NMR (100 MHz; CDCl_3_ + CD_3_OD): *δ* 129.0, 128.2, 126.4, 125.8, 68.8, 53.5, 52.0, 43.6, 43.1, 41.7, 40.5, 35.0, 31.0, 30.5. HRMS *m*/*z*: [M + H]^+^ calculated for C_21_H_31_N_2_O_4_S, 407.1999; found, 407.2005.

**General procedure F for the synthesis of compounds 11a–c:** Compound **4f** (0.5 g) was dissolved in 5 mL toluene. To the solution were added suitable bicycloamines (1.2 equiv.) and triethylamine (1.5 equiv.) as a base. The reaction mixture was heated at 80 °C for 3 h. The solvent was removed under a vacuum, extracted with DCM, and washed with brine solution. The residue was purified by silica gel column chromatography to get compounds **11a–c** as white solids.

**Synthesis of *N*-(bicyclo [1.1.1]pentan-1-ylcarbamoyl)-4-(trifluoromethoxy)benzenesulfonamide (11a):** Compound **4f** (0.5 g, 1.67 mmol) was dissolved in 5 mL toluene. To the solution were added bicyclo [1.1.1]pentan-1-amine hydrochloride (0.24 g, 2.00 mmol) and triethylamine (0.35 mL, 2.51 mmol) as a base. The reaction was carried out following general procedure F to afford compound **11a** as a white solid (0.41 g, 69%). Mp 157–159 °C; ^1^H NMR (400 MHz; CDCl_3_): *δ* 7.94 (d, *J* = 8.0 Hz, 2H), 7.36 (d, *J* = 8.0 Hz, 2H), 2.45 (s, 1H), 2.04 (s, 6H). ^13^C NMR (100 MHz; CDCl_3_): *δ* 150.8, 137.8, 129.3, 121.1, 52.7, 48.3, 24.5. HRMS *m*/*z*: [M + H]^+^ calculated for C_13_H_14_F_3_N_2_O_4_S, 351.0626; found, 351.0620.

**Synthesis of *N*-((3-cyanobicyclo [1.1.1]pentan-1-yl)carbamoyl)-4-(trifluoromethoxy)benzenesulfonamide (11b):** Compound **4f** (0.5 g, 1.67 mmol) was dissolved in 5 mL toluene. To the solution were added 3-aminobicyclo [1.1.1]pentane-1-carbonitrile hydrochloride (0.29 g, 2.00 mmol) and triethylamine (0.35 mL, 2.51 mmol) as a base. The reaction was carried out following general procedure F to get compound **11b** as a white solid (0.45 g, 72%). Mp 222–224 °C; ^1^H NMR (400 MHz; CDCl_3_): *δ* 7.95 (d, *J* = 8.0 Hz, 2H), 7.29 (d, *J* = 8.0 Hz, 2H), 2.40 (s, 6H). ^13^C NMR (100 MHz; CDCl_3_ + CD_3_OD): *δ* 152.7, 150.9, 137.9, 129.8, 120.8, 117.0, 56.7, 48.8, 21.5. HRMS *m*/*z*: [M + H]^+^ calculated for C_14_H_13_F_3_N_3_O_4_S, 376.0579; found, 376.0580.

**Synthesis of *N*-((3-fluorobicyclo [1.1.1]pentan-1-yl)carbamoyl)-4-(trifluoromethoxy)benzenesulfonamide (11c):** Compound **4f** (0.5 g, 1.67 mmol) was dissolved in 5 mL toluene. To the solution were added 3-fluorobicyclo [1.1.1]pentan-1-amine hydrochloride (0.28 g, 2.00 mmol) and triethylamine (0.35 mL, 2.51 mmol) as a base. The reaction was performed following general procedure F to afford compound **11c** as a white solid (0.39 g, 63%). Mp 191–193 °C; ^1^H NMR (400 MHz; CDCl_3_): *δ* 7.96 (d, *J* = 8.0 Hz, 1H), 7.30 (d, *J* = 8.0 Hz, 2H), 2.27 (s, 6H). ^13^C NMR (100 MHz; CDCl_3_ + CD_3_OD): *δ* 152.7, 147.4, 138.2, 129.7, 120.9, 55.4, 55.2. HRMS *m*/*z*: [M + H]^+^ calculated for C_13_H_13_F_4_N_2_O_4_S, 369.0532; found, 369.0537.

**Synthesis of *N*-((adamantan-2-yl)carbamoyl)-4-(trifluoromethoxy)benzenesulfonamide (12):** 5.0 g sulfonyl carbamate **3f** (16.71 mmol) was dissolved in 50 mL toluene. To the solution, 2-adamantylamine hydrochloride (2.50 g, 13.37 mmol) was added, and the reaction mixture was refluxed for 4 h. Toluene was evaporated, and to it, isopropanol was added and triturated to produce **12** as a white solid product (4.32 g, 62%). Mp 153–155 °C; ^1^H NMR (400 MHz; CDCl_3_): *δ* 8.86 (s, 1H), 7.97 (d, *J* = 8.0 Hz, 2H), 7.34 (d, *J* = 8.0 Hz, 2H), 6.93 (d, *J* = 8.0 Hz, 1H), 3.88 (d, *J* = 8.0 Hz, 1H), 1.85–1.78 (m, 7H), 1.73–1.62 (m, 7H). ^13^C NMR (100 MHz; CDCl_3_): *δ* 153.0, 150.7, 137.9, 129.3, 121.1, 54.6, 37.4, 37.0, 32.1, 27.2. HRMS *m*/*z*: [M + H]^+^ calculated for C_18_H_22_F_3_N_2_O_4_S, 419.1252; found, 419.1252.

**Synthesis of *N*-((4-(trifluoromethoxy)phenyl)sulfonyl)-4-(trifluoromethyl)piperidine-1-carboxamide (13):** 5.0 g sulfonyl carbamate **3f** (16.71 mmol) was dissolved in 50 mL toluene. To the solution, 2-adamantylamine hydrochloride (2.05 g, 13.37 mmol) was added, and the reaction mixture was refluxed for 4 h. Toluene was evaporated, and to it, isopropanol was added and triturated to provide **13** as a white solid product (4.21 g, 60%). Mp 84–86 °C; ^1^H NMR (400 MHz; CDCl_3_): *δ* 7.97 (d, *J* = 8.0 Hz, 2H), 7.33 (d, *J* = 8.0 Hz, 2H), 4.38 (d, *J* = 12.0 Hz, 1H), 3.48 (d, *J* = 12.0 Hz, 1H), 2.92–2.65 (m, 2H), 2.24–2.13 (m, 2H), 1.97–1.80 (m, 4H). ^13^C NMR (100 MHz; CDCl_3_): *δ* 159.0, 140.3, 128.8, 128.5, 121.1, 120.7, 43.0, 24.6, 21.5. HRMS *m*/*z*: [M + H]^+^ calculated for C_14_H_15_F_6_N_2_O_4_S, 421.0657; found, 421.0664.

**Synthesis of N-(cyclohexylcarbamoyl)-4-(trifluoromethoxy)benzenesulfonamide (14):** 5.0 g sulfonyl carbamate **3f** (16.71 mmol) was dissolved in 50 mL toluene. To the solution, cyclohexanamine (1.33 g, 13.37 mmol) was added, and the reaction mixture was refluxed for 4 h. Toluene was evaporated, and to it, isopropanol was added and triturated to produce **14** as a white solid product (3.45 g, 56%). Mp 141–143 °C; ^1^H NMR (400 MHz; CDCl_3_): *δ* 8.86 (s, 1H), 7.94 (d, *J* = 8.0 Hz, 2H), 7.34 (d, *J* = 8.0 Hz, 2H), 6.42 (d, *J* = 8.0 Hz, 1H), 3.61 (d, *J* = 8.0 Hz, 1H), 1.83 (d, *J* = 8.0 Hz, 2H), 1.67 (d, *J* = 8.0 Hz, 2H), 1.59 (d, *J* = 8.0 Hz, 1H), 1.38–1.29 (m, 2H), 1.22–1.14 (m, 3H). ^13^C NMR (100 MHz; CDCl_3_): *δ* 153.0, 150.8, 137.9, 129.3, 121.1, 119.0, 49.3, 32.9, 25.4, 24.5. HRMS *m*/*z*: [M + H]^+^ calculated for C_14_H_18_F_3_N_2_O_4_S, 367.0939; found, 367.0937.

**Synthesis of *N*-((3,5-dimethyladamantan-1-yl)carbamoyl)-4-(trifluoromethoxy)benzenesulfonamide (15a):** 5.0 g sulfonyl carbamate **3f** (16.71 mmol) was dissolved in 50 mL toluene. To the solution, 3,5-dimethyladamantan-1-amine (2.88 g, 13.37 mmol) and triethylamine (3.48 mL, 25.07 mmol) were added, and the reaction mixture was refluxed for 3 h. Toluene was evaporated, and to it, isopropanol was added and triturated to afford **15a** as a white solid product (4.19 g, 56%). Mp 124–126 °C; ^1^H NMR (400 MHz; CDCl_3_): *δ* 7.96 (d, *J* = 8.0 Hz, 2H), 7.34 (d, *J* = 8.0 Hz, 2H), 6.21 (s, 1H), 2.11 (s, 1H), 1.57–1.47 (m, 6H), 1.35–1.24 (m, 6H), 0.82 (s, 6H). ^13^C NMR (100 MHz; CDCl_3_): *δ* 129.3, 121.0, 53.8, 50.5, 48.6, 47.7, 42.5, 40.2, 32.5, 30.1. HRMS *m*/*z*: [M + H]^+^ calculated for C_20_H_26_F_3_N_2_O_4_S, 447.1565; found, 447.1571.

**Synthesis of *N*-((3,5-dimethyladamantan-1-yl)carbamoyl)naphthalene-2-sulfonamide (15b):** 5 0g sulfonyl carbamate **3l** (18.85 mmol) was dissolved in 50 mL toluene. To the solution, 3,5-dimethyladamantan-1-amine (3.25 g, 15.08 mmol) and triethylamine (3.92 mL, 28.28 mmol) were added, and the reaction mixture was refluxed for 3 h. Toluene was evaporated, and to it, isopropanol was added and triturated to afford **15b** as a white solid product (4.34 g, 63%). Mp 190–192 °C; ^1^H NMR (400 MHz; CDCl_3_): *δ* 8.46 (s, 1H), 7.98 (d, *J* = 8.0 Hz, 2H), 7.93 (d, *J* = 8.0 Hz, 1H), 7.71–7.63 (m, 2H), 7.41 (s, 1H), 6.41 (s, 1H), 2.11 (s, 1H), 1.74 (s, 2H), 1.54–1.48 (m, 4H), 1.29 (q, *J* = 12.0 Hz, 4H), 1.11 (s, 2H), 0.81 (s, 6H). ^13^C NMR (100 MHz; CDCl_3_): *δ* 149.9, 136.7, 134.9, 129.9, 129.5, 129.4, 128.7, 128.1, 128.0, 121.8, 53.8, 50.5, 47.7, 42.5, 40.2, 32.5, 30.0. HRMS *m*/*z*: [M + H]^+^ calculated for C_23_H_29_N_2_O_3_S, 413.1899; found, 413.1906.

**Synthesis of *tert*-butyl 4-(3-((4-(trifluoromethoxy)phenyl)sulfonyl)ureido)piperidine-1-carboxylate (16):** Compound **3f** (2 g, 6.68 mmol) was dissolved in 20 mL toluene. To the solution was added *tert*-butyl 4-aminopiperidine-1-carboxylate (1.1 g, 5.34 mmol), and the reaction mixture was refluxed for 2 h. The solvent was removed under a vacuum, extracted with DCM, and washed with a brine solution. The residue was purified by silica gel column chromatography to produce compound **16** as a white solid (2.51 g, 80%). Mp 174–176 °C; ^1^H NMR (400 MHz; CDCl_3_): *δ* 8.51 (s, 1H), 7.95 (d, *J* = 8.0 Hz, 2H), 7.35 (d, *J* = 8.0 Hz, 2H), 6.39 (d, *J* = 8.0 Hz, 1H), 3.97–3.77 (m, 2H), 2.85 (t, *J* = 12.0 Hz, 2H), 1.85 (d, *J* = 12.0 Hz, 2H), 1.64 (s, 1H), 1.44 (s, 9H), 1.34 (d, *J* = 8.0 Hz, 2H). ^13^C NMR (100 MHz; CDCl_3_): *δ* 154.8, 150.6, 137.7, 129.4, 121.1, 80.1, 47.8, 32.0, 28.5. HRMS *m*/*z*: [M + Na]^+^ calculated for C_18_H_24_F_3_N_3_O_6_SNa, 490.1236; found, 490.1234.

**Synthesis of *N*-(piperidin-4-ylcarbamoyl)-4-(trifluoromethoxy)benzenesulfonamide (17):** Compound **16** (2 g, 4.27 mmol) was dissolved in 8 mL DCM. Trifluoroacetic acid (1.27 mL, 17.08 mmol) was added to the solution, and the reaction mixture was stirred at room temperature for 2 h. The solvent was removed under a vacuum, extracted with DCM, and washed with a brine solution. The residue was purified by silica gel column chromatography to afford compound **17** as a white solid (1.31 g, 83%). Mp > 250 °C; ^1^H NMR (400 MHz; CD_3_OD): *δ* 7.98 (s, 2H), 7.57 (s, 1H), 7.36 (s, 1H), 3.35 (s, 4H), 3.02 (s, 4H), 2.90 (s, 1H). ^13^C NMR (100 MHz; CDCl_3_ + CD_3_OD): *δ* 167.3, 166.4, 147.6, 132.2, 124.8, 124.3, 40.4, 35.2, 33.5. HRMS *m*/*z*: [M + H]^+^ calculated for C_13_H_17_F_3_N_3_O_4_S, 368.0892; found, 368.0890.

**Synthesis of *N*-(1-((adamantan-1-yl)carbamoyl)piperidin-4-yl)-N′-((4-(trifluoromethoxy)phenyl)sulfonyl)carbamimidic acid (18):** Compound **17** (0.5 g, 1.36 mmol) was dissolved in 5 mL DMF. 1-Isocyanatoadamantane (0.36 g, 2.04 mmol) and triethylamine (0.38 mL, 2.72 mmol) were added to the solution, and the reaction mixture was stirred at room temperature overnight. The solvent was removed under a vacuum and the reaction mixture was neutralized with a 1N HCl solution. The white solid obtained was purified by silica gel column chromatography to afford compound **18** (0.47 g, 63%). Mp 171–173 °C; ^1^H NMR (400 MHz; CDCl_3_): *δ* 8.02 (d, *J* = 8.0 Hz, 2H), 7.33 (d, *J* = 8.0 Hz, 2H), 6.39 (d, *J* = 8.0 Hz, 1H), 4.29 (s, 1H), 3.73 (d, *J* = 12.0 Hz, 2H), 2.84 (d, *J* = 12.0 Hz, 2H), 2.04 (s, 4H), 1.93 (s, 6H), 1.83 (s, 1H), 1.64 (t, *J* = 16.0 Hz, 6H), 1.35 (d, *J* = 8.0 Hz, 2H). ^13^C NMR (100 MHz; CDCl_3_): *δ* 156.8, 138.0, 130.0, 120.9, 51.6, 47.3, 43.1, 42.5, 36.4, 31.8, 29.6. HRMS *m*/*z*: [M + H]^+^ calculated for C_24_H_32_F_3_N_4_O_5_S, 545.2046; found, 545.2041.

### 4.2. Aqueous Solubility (Bienta Ltd.)

Aliquots of 5 µL of 20.00 mM, 10.00 mM, 5.00 mM, 2.50 mM, 1.25 mM, 0.63 mM, 0.31 mM, 0.16 mM DMSO stock solutions of the control (highly soluble 2′-deoxy-5-fluorouridine and insoluble raloxifen hydrochloride) and the test compounds were loaded in 96-well microplates with clear flat bottoms in duplicates. Then, 250 µL of PBS was dispensed into each well using a Multidrop Combi dispenser to reach the concentrations of 400.0 µM, 200.0 µM, 100.0 µM, 50.0 µM, 25.0 µM, 12.5 µM, 6.25 µM, 3.125 µM for each compound with 2% final DMSO. Then, the resulting solutions were incubated for 15 min at room temperature. The effective range of this assay is approximately 5–200 µM. The solubility of each compound was calculated using the dedicated fitting algorithm. The physical base of nephelometric titration fitting is based on the quick increase in turbidity when a concentration of the tested compound reaches its solubility level. An intersection of the regression line of the high-level turbidity points with the calculated baseline was plotted for each compound. The baseline is a linear regression line plotted for fully soluble control compound DOFU. Each concentration point signal of the test article was compared with the corresponding signal of DOFU. Concentration points with a statistically higher signal of the test article compared to DOFU were assigned as concentrations with some compound sedimentation and then were used for linear fit plotting. The abscissa of the intersection of a test article and DOFU linear fits is, therefore, the kinetic solubility of the compound.

### 4.3. Caco-2 Permeability (Bienta Ltd.)

Caco-2 cells were cultured in 75 cm^2^ flasks to 80–90% confluence according to the ATCC and Millipore recommendations in a humidified atmosphere at 37 °C and 5% CO_2_. Cells were detached with a trypsin/EDTA solution, resuspended in the complete medium containing DMEM high glucose (4.5 mg/L) with L-glutamine (4 mM) supplemented with 10% heat-inactivated Fetal Bovine Serum, 1% non-essential amino acids, and 730 nM puromycin, and seeded at a density of 5 × 105 cells in a 75 cm^2^ flask. After 5 days, cells were trypsinized and resuspended in the complete medium to a final concentration of 600 × 10^3^ cells/mL. Of the cell suspension, 400 µL was added to each well of an HTS 24-Multiwell Insert System and 25 mL of prewarmed complete medium was added to the feeder tray. Caco-2 cells were incubated in the Multiwell Insert System for 6–10 days before the transport experiments. The medium in the filter plate and feeder tray was refreshed every other day. Prior to the transport experiment, the integrity of the monolayer was verified by measuring the transepithelial electrical resistance (TEER) for every well using a Millicell-ERS system ohm meter. The final TEER values were within the range of 150–600 Ω × cm^2^, as required for the assay conditions. The 24-well insert plate was removed from its feeder plate and placed in a new sterile 24-well transport analysis plate. The inserts were washed with PBS after medium aspiration. Ketoprofen, atenolol, digoxin, and quinidine were used as reference compounds.

To determine the rate of the compounds’ transport in the apical (A)-to-basolateral (B) direction, 300 µL of the test compound dissolved in transport buffer (Hanks’ BSS (9.5 g/L) and NaHCO_3_ (0.35 g/L) with MgSO_4_ to final concentration 0.81 mM, CaCl_2_ to final concentration 1.26 mM, HEPES to final concentration 25 mM, pH adjusted to 7.4) was added into the filter wells; 1000 μL of transport buffer was added to the transport analysis plate wells. To determine transport rates in the basolateral (B)-to-apical (A) direction, 1000 μL of the test compound solutions was added into the wells of the transport analysis plate, and the wells in the filter plate were filled with 300 μL of buffer (apical compartment).

The effect of the inhibitor on the Pgp-mediated transport of the tested compounds was assessed by determining the bidirectional transport in the presence or absence of verapamil. The Caco-2 cells were preincubated for 30 min at 37 °C with 100 μM of verapamil in both apical and basolateral compartments. After removal of the preincubation medium, the test compounds with verapamil (100 μM) in transport buffer were added in donor wells, while the receiver wells were filled with the appropriate volume of transport buffer with 100 μM of verapamil. The final amounts of test and reference compounds were 10 μM each. The plates were incubated for 90 min at 37 °C under continuous shaking at 100 rpm. Aliquots of 75 μL were taken from the donor and receiver compartments for LC–MS/MS analysis. All samples were mixed with 2 volumes of acetonitrile followed by protein sedimentation by centrifuging at 10,000 rpm for 10 min. Supernatants were analyzed using the HPLC system coupled with a tandem mass spectrometer.

### 4.4. Plasma Protein Binding (Bienta Ltd.)

The assay was performed in a multiple-use 96-well dialysis unit (HTD96b dialyzer). Each individual well unit consisted of 2 chambers separated by a vertically aligned dialysis membrane of predetermined pore size (MWCO 12–14 kDa). An amount of 120 μL of non-diluted plasma spiked with the compound (1 μM, final DMSO concentration 1%) was added to one chamber, and the same volume of PBS buffer with pH 7.4 was added to the other chamber. The HTD96b dialyzer was covered with adhesive sealing film and incubated at 37 °C and shaken at 100 rpm for 5 h. For sample preparation, an aliquot of the content of each chamber was mixed with the same volume of the blank opposite matrix. To define the non-specific loss of the compound during this assay, a standard solution was created by mixing an aliquot of spiked plasma with a blank buffer without dialysis. Two aliquots of the standard solution were incubated at 37 °C and shaken at 100 rpm for 5 h (recovery samples). The other two aliquots were immediately diluted with acetonitrile and stored at 4 °C until LC–MS/MS analysis (stability samples). All samples were diluted 5-fold with 100% methanol with subsequent plasma protein sedimentation by centrifuging at 6000 rpm for 5 min. Supernatants were analyzed using the HPLC system coupled with a tandem mass spectrometer.

### 4.5. Microsomal Stability (Bienta Ltd.)

Human microsomal incubations were carried out in 96-well plates in 5 aliquots of 30 μL each (one for each time point). Human liver microsomal incubation medium comprised of phosphate buffer (100 mM, pH 7.4), MgCl_2_ (3.3 mM), NADPH (3 mM), glucose-6-phosphate (5.3 mM), glucose-6-phosphate dehydrogenase (0.67 units/mL) with 0.42 mg of liver microsomal protein per ml. In the control reactions, the NADPH-cofactor system was substituted with phosphate buffer. Test compounds (2 μM, final solvent concentration 1.6%) were incubated with microsomes at 37 °C and shaken at 100 rpm. Each reaction was performed in duplicates. Five time points over 40 min were analyzed. The reactions were stopped by adding 5 volumes of methanol containing the internal standard to the incubation aliquots, followed by protein sedimentation by centrifuging at 5500 rpm for 4 min. Supernatants were analyzed using the HPLC system coupled with a tandem mass spectrometer.

### 4.6. Molecular Docking Study

AUDA, **4f**, **4l**, and **15a** compounds were docked into the active site of human sEH. **4f** and other sulfonyl urea-based sEHis were docked into PDB:6FR2 using ICM-Pro software version 3.9-3b (Molsoft, San Diego, CA, USA) [46,47]. Residues involved in contacts with the compounds are numbered. The PDB files for the docking study are included in the Supplementary Materials.

### 4.7. In Vitro sEH Activity Assay

A fluorescence-based sEH activity assay provided a convenient method for screening human epoxide hydrolase inhibitors. The assay utilized (3-Phenyl-oxiranyl)-acetic acid cyano-(6-methoxy-naphthalen-2-yl)-methyl ester (PHOME) as a substrate. When the epoxide moiety of PHOME was hydrolyzed by epoxide hydrolase, an intramolecular cyclization occurred, which resulted in the release of a cyanohydrin under basic conditions. The cyanohydrin quickly decomposed into cyanide ions and the highly fluorescent 6-methoxy-2-naphthaldehyde, which was analyzed using an excitation wavelength of 330 nm and emission wavelength of 465 nm [52]. A fluorescence-based sEH activity assay kit was obtained from Cayman Chemical (item number: 10011671). The inhibitory function of the compounds on sEH activity was tested as detailed in the product manual from Cayman Chemical. Briefly, 3 mL sEH assay buffer (10×) was diluted with 27 mL HPLC-grade water. This final assay buffer, 25 mM bis-tris (pH 7.0), was used in the assay and for the dilution of the soluble epoxide hydrolase (sEH) enzyme. Amounts of 12 μL each of human and mouse recombinant sEH were thawed on ice. Prior to assaying, 588 μL cold assay buffer was added directly to the enzyme vial. An amount of 190 μL assay buffer and 5 μL solvent (40% DMSO in assay buffer) were added to the background wells. In the 100% initial activity wells, 185 μL assay buffer, 5 μL sEH, and 5 μL solvent were added. In inhibitor wells, 185 μL assay buffer, 5 μL solution of the synthesized compounds, and 5 μL of recombinant enzyme solution were added. To start the reaction, 5 μL of PHOME was added to each well at a final concentration of 0.25 mM. The microwell plate was shaken carefully for 10 s and covered with a plate cover. It was incubated for 15 min at 25 °C. The cover plate was removed, and fluorescence was read using an excitation wavelength of 330 nm and emission wavelength of 465 nm.

Average fluorescence (AF) of the background wells and 100% initial activity in the wells and of each of the inhibitors were determined. The background AF was subtracted from the 100% initial activity and inhibitor AFs. The % sEH activity is calculated as (inhbitited AF/% 100 initial activity) × 100.

To calculate the half maximal inhibitory concentration (IC_50_) values of the potent inhibitors, a dose-dependent fluorescence assay was performed. Multiple concentrations of the inhibitors were tested followed by plotting of the percent activity as a function of inhibitor concentration to determine the IC_50_ values.

### 4.8. In Vivo Pharmacokinetic Experiment

Twenty 8–9-week-old male C57BL/6J mice were taken for the in vivo pharmacokinetic experiment. They were divided into four groups, each containing five mice. Each group was used for either oral or intraperitoneal administration of compound **4f** or **15a**. Both the compounds were reconstituted in 5% DMSO, 5% TWEEN 80 in saline water (pH = 7.4). The mice were orally and intraperitoneally administered compounds at 10 mg/kg dose. Blood samples were collected at 0, 0.5, 1, 2, 4, 6, and 24 h after administration of the compound, and serum was isolated with the compound. For the pharmacokinetic study, 10 μL of serum samples from mice were extracted with a 2:1 mixture of LC/MS-grade chloroform and methanol, followed by intermittent vortexing, and then centrifuged at 4000 rpm for 10 min at 4 °C. Thereafter, the supernatant was collected and analyzed by LC−MS/MS to measure the concentrations of compounds **4f** and **15a** in the serum [53].

### 4.9. Lipopolysaccharide (LPS) Induced Acute Lung Injury (ALI)

LPS-induced acute lung injury induced by a single oropharyngeal instillation of lipopolysaccharide (LPS) (50 µg/mouse) in 16-week-old mice as described previously [54]. Briefly, LPS (L4391, MilliporeSigma, Burlington, MA, USA) was dissolved in sterile saline and administered at 50 µg/mouse in 40 µL after anesthesia induced by ketamine/xylazine. The mice were studied 24 h after LPS or saline instillation.

### 4.10. Bronchoalveolar Lavage Collection from Mice (BALF)

BALF was collected from anesthetized mice by lavaging lungs two times with 0.8 mL Hanks’ balanced salt solution without calcium and magnesium (HBSS) (Sigma-Aldrich, St. Louis, MI, USA). Supernatants were collected as BALF for further analyses.

### 4.11. Total Cell Count in Bronchoalveolar Lavage

The number of cells in the BALF was estimated using an automated cell counter, the Countess II FL cell counter (Thermo Fisher Scientific Inc., Waltham, MA, USA).

### 4.12. Level of Albumin in BALF

Albumin levels were measured by the Mouse Albumin Elisa Assay kit (Cat # E99-134), which was obtained from Bethyl Laboratories. BALF samples from the LPS group were diluted at a 1:2000 ratio.

### 4.13. Level of Total Protein in BALF

Total protein in BALF samples was measured using the Pierce BCA protein assay kit (Cat # 23225) as detailed in the product manual (Thermo Fisher Scientific).

### 4.14. RNA Isolation and qPCR

The right middle lobe was immediately placed in an RNA later solution and stored at −80 °C until the RNA extraction procedure. RNA extraction was performed using RNeasy Mini Kits from Qiagen (Valencia, CA, USA). One microgram of total RNA was reverse transcribed to cDNA using an iScript cDNA synthesis kit (Bio-Rad, Hercules, CA, USA). Expression of the target genes was quantified using gene-specific primers and PowerSYBRGreen master mix (Thermo Fisher Scientific) in a QuantStudio 3 Real-Time PCR instrument (Thermo Fisher Scientific). Predesigned mouse *IL-6* (QT00098875), *TNF-α* (QT00104006), *IL-1β* (QT01048355), *CXCL1* (QT00115647), *CCL2* (QT00167832), *CXCL3* (QT00151599) and *CXCL9* (QT00097062) primers were purchased from Qiagen (Valencia, CA, USA). Fold changes in gene expression relative to the housekeeping gene TATA-Box Binding Protein (Tbp, QT00198443) were determined. Gene expression values were calculated based on the 2^∆∆Ct^ method.

### 4.15. Tail-Flick Reflex Model

A tail-flick reflex test was performed as previously described [55,56]. The parameters of the tail-flick instrument (7360, Ugo Basile Instruments, Varese, Italy) were set up to keep the baseline at 2.5–4.0 s. Mice were fixed and the part about 1.5–2 cm away from the tail of the mouse was aligned with the infrared emission hole of the instrument. The time the mouse spends on the infrared emission hole surface is automatically recorded on the instrument when the mouse shows tail flick. Latencies of mice of less than 2 s or more than 5 s were excluded. After the detection of the baseline (0 min), AUDA (10 mg/kg) and compounds **4f** and **4l** (10 mg/kg) or the vehicle (1:1:18 ratio DMSO/TWEEN 80/saline) were administrated by intraperitoneal (i.p.) injection, and the latencies were measured at 15, 30, 60, 90, and 120 min after the drugs’ injection. The cut-off time in the tail-flick reflex test was set at 8 s to avoid sensitization and damage to the skin and to achieve the maximum possible effect (MPE%) = (measured value − basic value) × 100/(cut-off − basic value). C57BL/6J male mice obtained from The Jackson Laboratory (Bar Harbor, ME, USA) weighing 28 g were used for the tail-flick test. Animal procedures were conducted following the rules and regulations of the Institutional Animal Care and Use Committee of the National Institute on Alcohol Abuse and Alcoholism (NIAAA) under the protocols of LPS-GK1.

### 4.16. Hot Plate Model

Thermal nociception in the hot plate test was assessed with a commercially available apparatus (Hotplate Analgesia Meter, Columbus Instruments) consisting of a metal plate to a constant temperature of 55.0 ± 0.1 °C. Mice were put into the hot plate testing instrument to adapt for at least 15 min. The device was connected to a manually operated timer that recorded the amount of time the mouse spent on the heated surface before showing signs of nociception (e.g., licked its paws or jumped), the cut-off time was 60 s. The baseline (basic pain threshold, the response was more than 25 s, and the jumping mice were excluded.) was calculated as the mean of three readings recorded before testing at intervals of 15 min. The time course of latency was then determined at 15, 30, 45, 60, 90, and 120 min after compound treatment. Data were analyzed as time-course curves of the percentage of maximum effect MPE% = (post drug latency − baseline latency) × 100/(cut-off time − baseline latency). C57BL/6J male mice obtained from The Jackson Laboratory (Bar Harbor, ME, USA) weighing 28 g were used for the hot plate test. Animal procedures were conducted following the rules and regulations of the Institutional Animal Care and Use Committee of the National Institute on Alcohol Abuse and Alcoholism (NIAAA) under the protocols of LPS-GK1. AUDA (10 mg/kg), compounds **4f** and **4l** (10 mg/kg), or the vehicle (1:1:18 ratio DMSO/TWEEN 80/saline) were injected intraperitoneally (i.p.) [57].

### 4.17. Statistical Analysis

Statistical analysis was performed using GraphPad Prism 9 (GraphPad Software Inc., San Diego, CA, USA). One-way ANOVA followed by Bonferroni’s multiple comparisons testing was performed. *p* < 0.05 was considered significant.

Statistical analyses for tail-flick and hot plate experiments were performed using GraphPad Prism 9 (GraphPad Software Inc.). Two-way ANOVA was followed by Tukey’s multiple comparisons testing.

## 5. Conclusions

The availability of high-quality EpFA and sEH inhibitors to stabilize them contributed to the discovery of a multitude of biological effects mediated by the CYP450 branch of the arachidonate cascade by several academic investigators worldwide. Substantial studies have shown that EpFAs play essential roles in the pathology of inflammation and neuropathic pain. Our study further demonstrates the underlying molecular mechanisms of EpFAs/sEHI actions as an analgesic strategy for inflammation and pain management. Recently, several clinical trials of sEH inhibitors targeting different diseases have emphasized the importance of sEH as a promising therapeutic target [21,32,45,58]. Strategic substitution at specific positions of sulfonyl ureas through structure–activity relationship development led to a set of potent sEH inhibitors in this study. Preliminary in vitro fluorescence assays and pharmacokinetic experiments identified compound **4f** as the most suitable candidate for in vivo studies. We performed tail-flick reflex and hot plate pain models to determine the analgesic efficacy of our lead. To our satisfaction, compound **4f** manifested significant efficacy in the tail-flick reflex pain model. Further, lipopolysaccharide (LPS)-induced acute lung injury (ALI) studies in mice demonstrated that our lead is more efficacious than known sEH inhibitor AUDA in reducing the gene expressions of several inflammatory cytokines in vivo. The results from this study show that sulfonyl urea derivatives show inhibition of sEH and suggest that the solubility and potency of the compounds can be optimized further. Collectively, these results pave the way for further optimization of sulfonyl urea derivatives as sEH inhibitors for the treatment of inflammation and pain.

## Figures and Tables

**Table 1:** Analysis of human sEH inhibition through structure–activity relationship (SAR) and their physicochemical properties. ^a^ tPSA (theoretical polar surface area) and ClogP obtained from ChemDraw 22.2.

Comp	Structure	Human sEH % Inhibition	tPSA/ClogP ^a^
**AUDA**	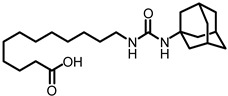	97.62	78.4/6.0
**4a**	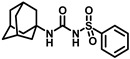	93.61	75.3/3.07
**4b**	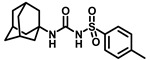	91.78	75.3/3.6
**4c**	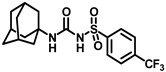	34.06	75.3/4.0
**4d**	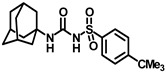	32.7	75.3/4.8
**4e**	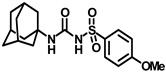	95.39	84.5/3.0
**4f**	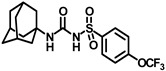	96.05	84.5/4.1
**4g**	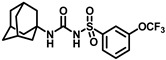	1.65	84.5/4.1
**4h**	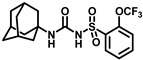	6.28	84.5/4.1
**4i**	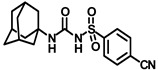	19.36	99.6/2.5
**4j**	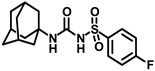	85.26	75.3/3.2
**4k**	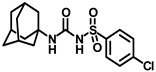	96.47	75.3/3.8
**4l**	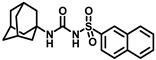	96.79	75.33/4.2
**6a**	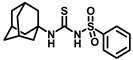	84.56	58.2/3.1
**6b**	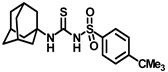	1.80	58.2/5.0
**6c**	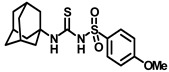	2.50	67.4/3.0
**6d**	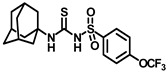	96.24	67.4/4.2
**7**	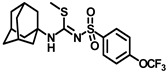	32.83	67.8/5.2
**8a**	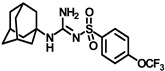	40.19	93.8/3.5
**8b**	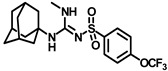	13.06	79.8/4.3
**9a**	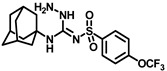	89.50	105.8/3.2
**9b**	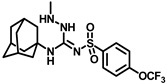	9.83	91.8/3.9
**9c**	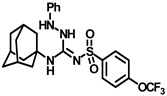	4.71	91.8/5.4
**9d**	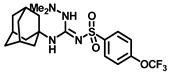	7.65	83.0/3.1
**10a**	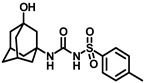	22.47	95.5/2.2
**10b**	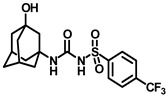	21.26	95.5/2.6
**10c**	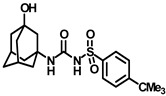	3.08	95.5/3.6
**11a**	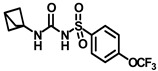	4.28	84.5/2.1
**11b**	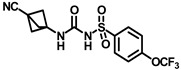	7.47	108.3/2.5
**11c**	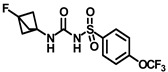	1.67	84.5/2.0
**12**	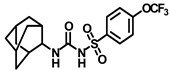	34.53	84.5/4.1
**13**	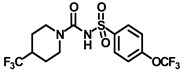	2.75	75.0/3.01
**14**	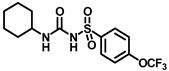	32.26	84.5/3.5
**15a**	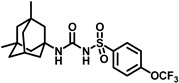	92.06	84.5/5.1
**15b**	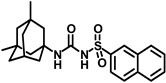	47.05	75.2/5.2
**16**	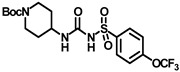	23.03	114.04/2.98
**17**	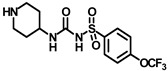	32.02	96.5/1.0
**18**	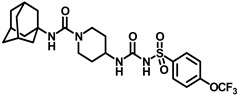	60.80	116.8/3.5

**Table 2:** Dose-dependent assay results for potent sEH inhibitors.

Comp	Structure	Human sEH Inhibition IC_50_	Mouse sEH Inhibition IC_50_
**AUDA**	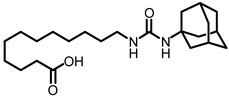	2.31 nM	26.99 nM
**4a**	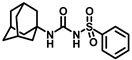	0.10 µM	>1 µM
**4b**	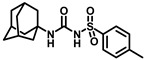	16.3 nM	0.85 µM
**4e**	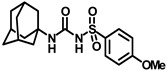	2.94 nM	>1 µM
**4f**	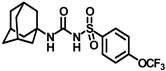	1.69 nM	0.29 µM
**4j**	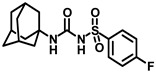	0.12 µM	>1 µM
**4k**	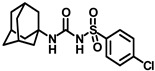	2.0 nM	>1 µM
**4l**	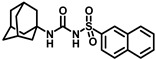	1.21 nM	0.19 µM
**6a**	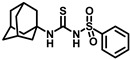	0.13 µM	>1 µM
**6d**	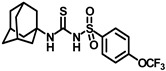	4.66 nM	0.11 µM
**9a**	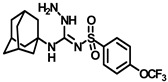	14.2 nM	>1 µM
**15a**	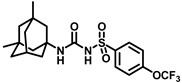	3.87 nM	22.01 nM
**15b**	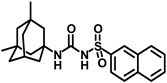	0.95 µM	>1 µM
**18**	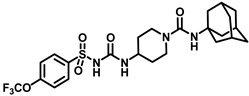	38.7 nM	0.71 µM

**Table 3:** In vitro pharmacokinetic profiles of potent compounds.

Comp	Aq. Solubility pH = 7.4 (μg/mL)	Caco-2 Permeability			Plasma Protein Binding				Human Metabolic Stability	
		Apical to Basal (P_app_) in cm/s (10^−6^)	Basal to Apical (P_app_) in cm/s (10^−6^)	Efflux Ratio	Human		Mouse			
					% of Bound Comp	% of Stability	% of Bound Comp	% of Stability	Half Life (min)	% Remaining after 1 h
**4f**	53	14.9	12.6	0.8	>99.9	99	98	106	106.3	80
**4l**	13	19.9	11.9	0.6	>99.9	99	98	88	33.4	45

## Data Availability

The data presented in this study are available on request from the corresponding author.

## Supplementary Materials

The following supporting information can be downloaded at https://www.mdpi.com/article/10.3390/molecules29133036/s1. This material is available free of charge via the Internet: Representative NMR spectra, HRMS and LCMS chromatograms and additional screening data.

## Abbreviations

sEH, soluble epoxide hydrolase; EETs, epoxyeicosatrienoic acids; SAR, structure–activity relationship; ADME, absorption, distribution, metabolism, and excretion; tPSA, theoretical polar surface area; PK, pharmacokinetics; LPS, lipopolysaccharide; ALI, acute lung injury.
