# Supplementary material for: Evaluation of the Therapeutic Potential of Sulfonyl Urea Derivatives as Soluble Epoxide Hydrolase (sEH) Inhibitors
Source: Molecules. 2024 Jun 26;29(13):3036. doi: 10.3390/molecules29133036 (PMC11242993; doi:10.3390/molecules29133036)
Supplement: Supplementary file 1 [file molecules-29-03036-s001.zip › molecules-3054635-supplementary.pdf]

Supporting Information

Rational Design, Synthesis, And Biological Evaluation of Therapeutically Effective Sulfonamide Derivatives as Soluble Epoxide Hydrolase (sEH) Inhibitors

Biswajit Kundu,[1] Szabolcs Dvoracsko,[1,2] Abhishek Basu,[2] Lenny Pommerolle,[2] Kyu Ah Kim,[1] Casey M. Wood,[1] Eve Gibbs,[1] Madeline Behee,[2] Nadya Tarasova,[3] Resat Cinar[2] and Malliga R. Iyer[1]*

[1]Section on Medicinal Chemistry, National Institute on Alcohol Abuse and Alcoholism (NIAAA), National Institutes of Health (NIH), 5625 Fishers Lane, Rockville, MD 20852, USA, [2]Section on Fibrotic Disorders, National Institute on Alcohol Abuse and Alcoholism (NIAAA), National Institutes of Health (NIH), 5625 Fishers Lane, Rockville, MD 20852, USA and [3]Laboratory of Physiologic Studies, National Institute on Alcohol Abuse and Alcoholism (NIAAA), National Institutes of Health (NIH), 5625 Fishers Lane, Rockville, MD 20852, USA. [3]

Email: malliga.iyer@nih.gov

Table of Contents

1. Caco-2 permeability	S2
2. Dose dependent assay	S3
3. In vivo pharmacokinetics of **4l**	S3
4. HRMS data	S4-S5
5. LCMS data of **4f**	S5
6. NMR spectroscopic data	S6-S42

1. Caco-2 permeability

A-B and B-A permeability data as well as the efflux ratio (P_{app}B-A/P_{app}A-B) data for the test and reference compounds are listed in the tables below.

Table S1. A-B and B-A permeability data

Test compound	P_{app} (AB), 10^{-6} cm/s				P_{app} (BA), 10^{-6} cm/s				Efflux ratio*
	1	2	Mean	SD	1	2	Mean	SD	
Ketoprofen	25.9	25.6	**25.7**	0.2	17.6	19.1	**18.3**	1.1	**0.7**
Atenolol	0.6	0.7	**0.6**	0.1					
Quinidine	11.1	14.9	**13.0**	2.7	32.4	25.3	**28.8**	5.1	**2.2**
Digoxin	0.2	0.2	**0.2**	0.0	12.5	12.2	**12.4**	0.2	**62.9**
**4f**	15.1	14.7	**14.9**	0.3	12.9	12.3	**12.6**	0.4	**0.8**
**4l**	21.8	18.1	**19.9**	2.7	11.5	12.2	**11.9**	0.5	**0.6**

* Efflux ratio is expressed as the quotient of P_{app}(BA) to P_{app}(AB)

Table S2. Recovery values for test and reference compounds

Test compound	Recovery, %											
	A-B			B-A			A-B in presence of Verapamil			B-A in presence of Verapamil		
	1	2	Mean	1	2	Mean	1	2	Mean	1	2	Mean
Ketoprofen	115	116	**116**	90	92	**91**						
Atenolol	98	104	**101**									
Quinidine	96	102	**99**	99	93	**96**	104	100	**102**	102	105	**104**
Digoxin	101	96	**99**	82	88	**85**	83	87	**85**	103	106	**105**
**4f**	72	67	**70**	70	65	**67**	*58*	*61*	**59**	71	76	**74**
**4l**	79	73	**76**	79	79	**79**	*52*	*54*	**53**	84	93	**89**

2. Dose dependent assay

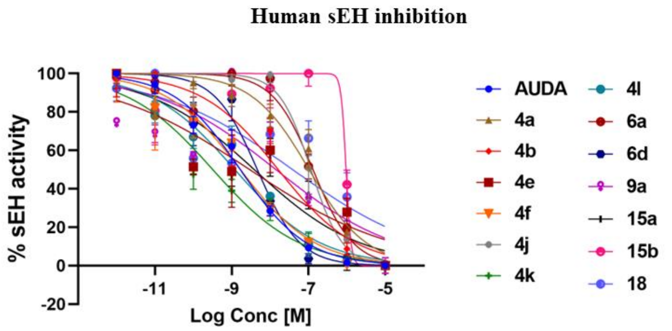

Compound	Human sEH IC_{50}	Compound	Human sEH IC_{50}
AUDA	2.31 nM	4l	1.21 nM
4a	0.10 µM	6a	0.13 µM
4b	16.3 nM	6d	4.66 nM
4e	2.94 nM	9a	14.2 nM
4f	1.69 nM	15a	3.87 nM
4j	0.12 µM	15b	0.95 µM
4k	2.0 nM	18	38.7 nM

Figure S1. Concentration-dependent inhibition of test compounds on recombinant human sEH.

3. In vivo pharmacokinetics of 4l

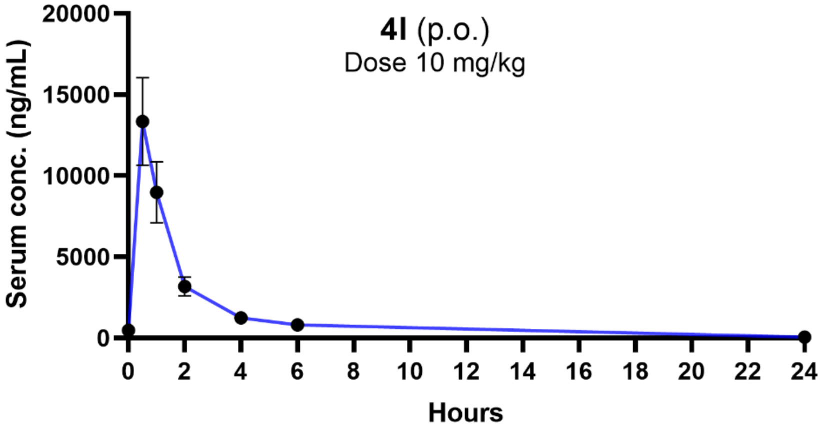

Figure S2. Concentration of **4l** in serum after single oral administration at 10 mg/kg dose.

2. HRMS data

Comp **4f**

Elemental Composition Report **Page 1**

Single Mass Analysis
Tolerance = 5.0 mDa / DBE: min = -1.5, max = 100.0
Element prediction: Off
Number of isotope peaks used for i-FIT = 3

Monoisotopic Mass, Even Electron Ions
51 formula(e) evaluated with 1 results within limits (up to 50 closest results for each mass)
Elements Used:
C: 0-100 H: 0-200 N: 2-2 O: 0-50 F: 3-3 32S: 1-1
BK-28FEB22-46 240 (4.076) AM2 (Ar,25000.0,0.00,0.00); ABS
TOF MS ES+

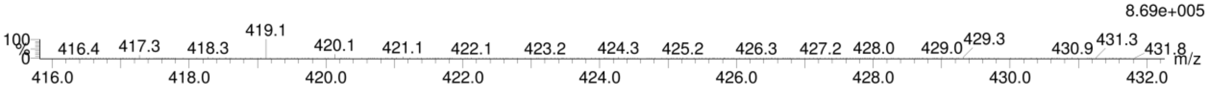

Minimum:				-1.5				
Maximum:		5.0	3.0	100.0				
Mass	Calc. Mass	mDa	PPM	DBE	i-FIT	Norm	Conf(%)	Formula
419.1250	419.1252	-0.2	-0.5	7.5	497.8	n/a	n/a	C18 H22 N2 O4 F3 32S

Comp **4l**

Elemental Composition Report **Page 1**

Single Mass Analysis
Tolerance = 5.0 mDa / DBE: min = -1.5, max = 100.0
Element prediction: Off
Number of isotope peaks used for i-FIT = 3

Monoisotopic Mass, Even Electron Ions
64 formula(e) evaluated with 1 results within limits (up to 50 closest results for each mass)
Elements Used:
C: 0-100 H: 0-200 N: 2-2 O: 0-50 32S: 1-1
BK-13DEC21-1-126 106 (1.810) AM2 (Ar,25000.0,0.00,0.00); ABS
TOF MS ES+

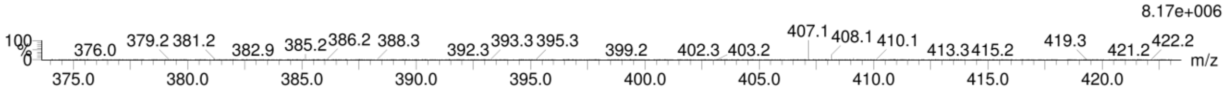

Minimum:				-1.5				
Maximum:		5.0	3.0	100.0				
Mass	Calc. Mass	mDa	PPM	DBE	i-FIT	Norm	Conf(%)	Formula
385.1582	385.1586	-0.4	-1.0	10.5	531.8	n/a	n/a	C21 H25 N2 O3 32S

Comp **15a**

Elemental Composition Report

Page 1

Single Mass Analysis

Tolerance = 5.0 mDa / DBE: min = -1.5, max = 100.0
Element prediction: Off
Number of isotope peaks used for i-FIT = 3

Monoisotopic Mass, Even Electron Ions
59 formula(e) evaluated with 1 results within limits (up to 50 closest results for each mass)
Elements Used:
C: 0-200 H: 0-200 N: 2-2 O: 0-30 F: 3-3 32S: 1-1
BK-12JAN24-352 112 (1.911) AM2 (Ar,25000.0,0.00,0.00); ABS
TOF MS ES+

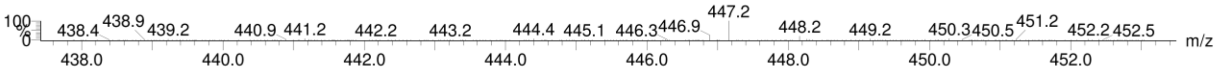

Minimum:				-1.5				
Maximum:		5.0	5.0	100.0				
Mass	Calc. Mass	mDa	PPM	DBE	i-FIT	Norm	Conf(%)	Formula
447.1571	447.1565	0.6	1.3	7.5	482.3	n/a	n/a	C20 H26 N2 O4 F3 32S

3. LCMS data of 4f

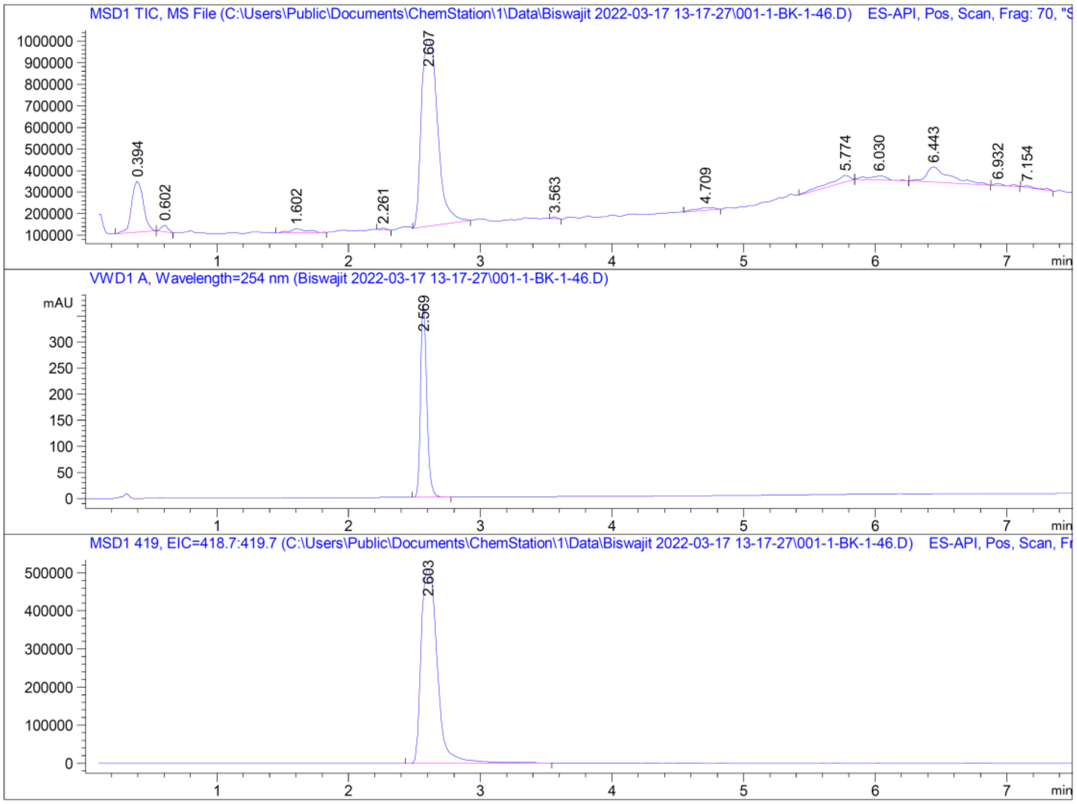

4. NMR spectra

^{1}H NMR of **4a**

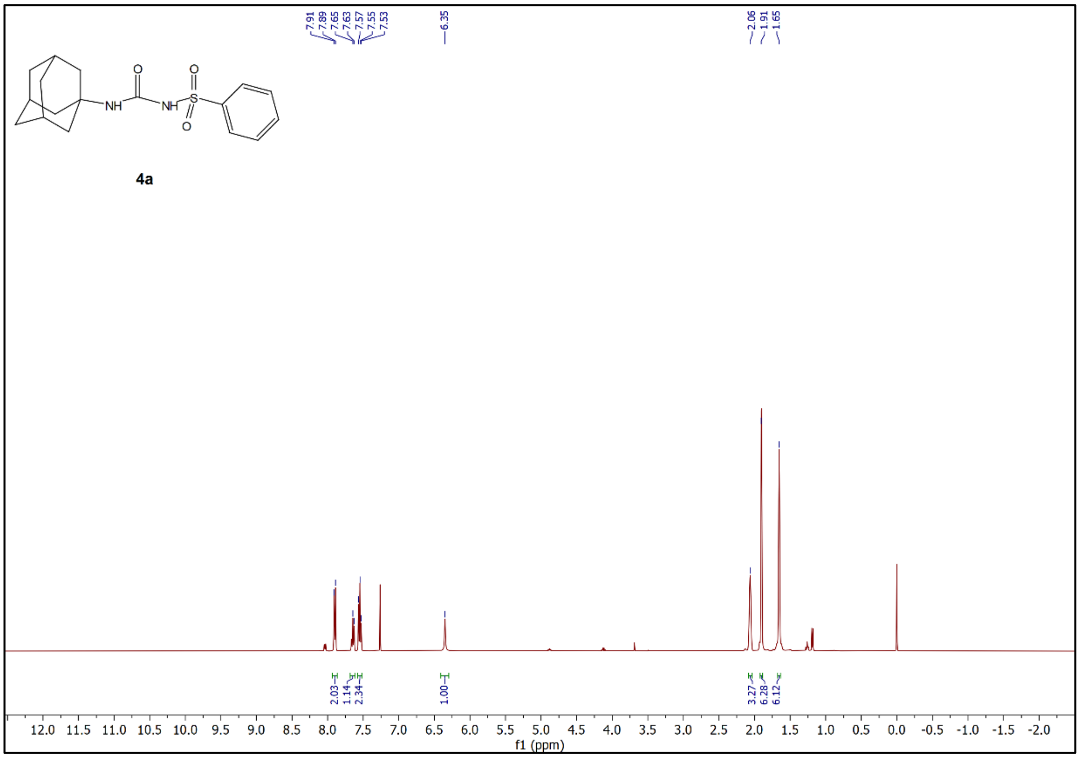

^{13}C NMR of **4a**

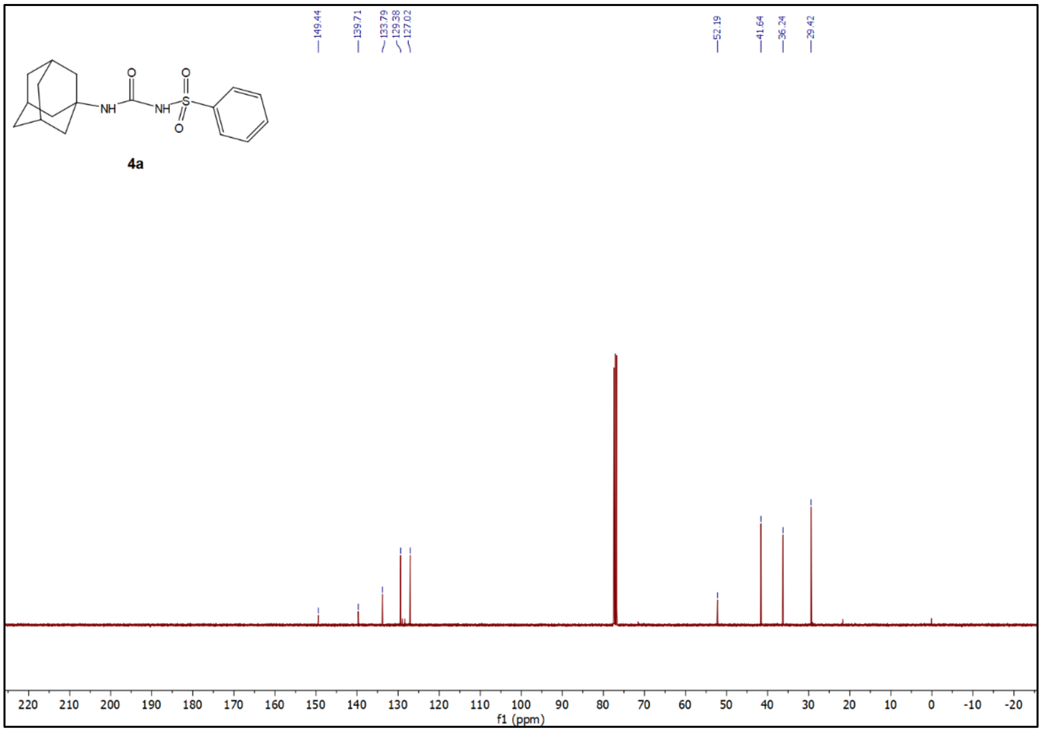

^{1}H NMR of **4b**

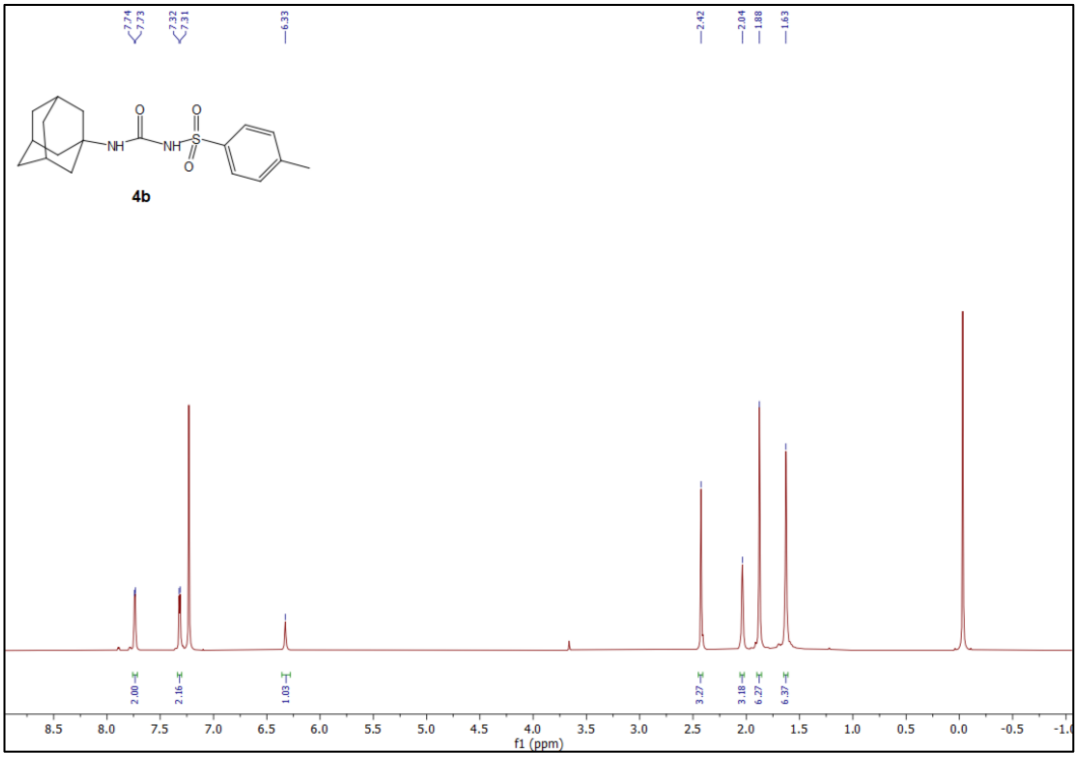

^{13}C NMR of **4b**

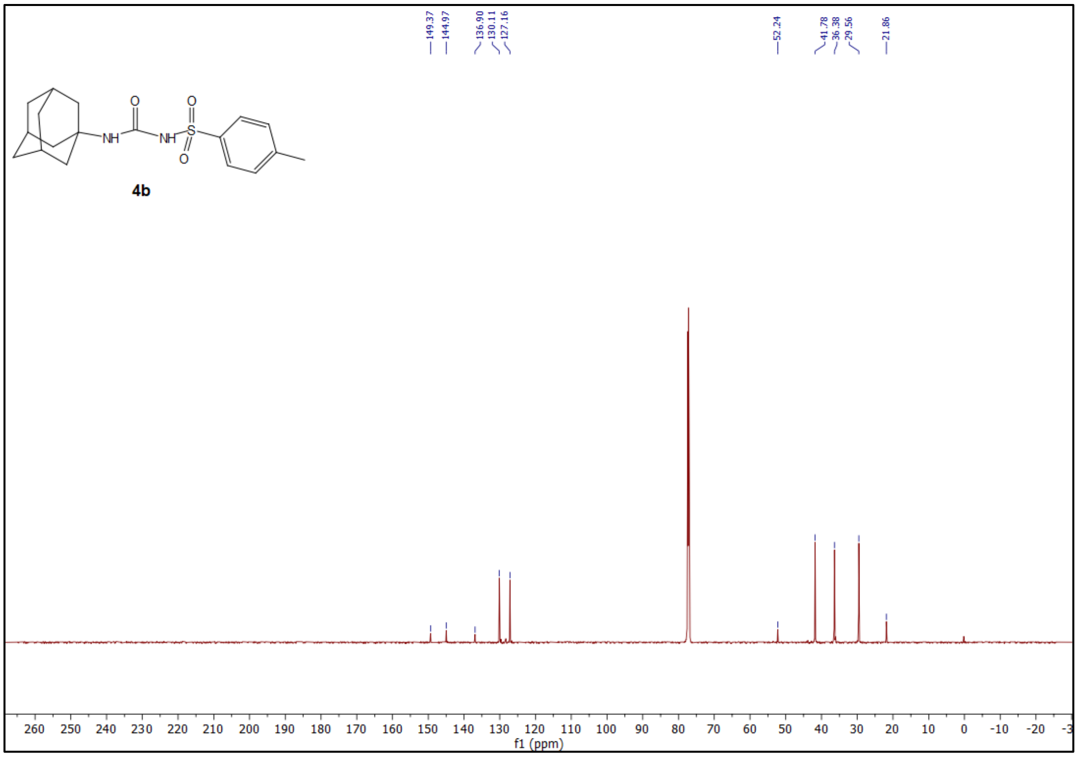

^{1}H NMR of **4c**

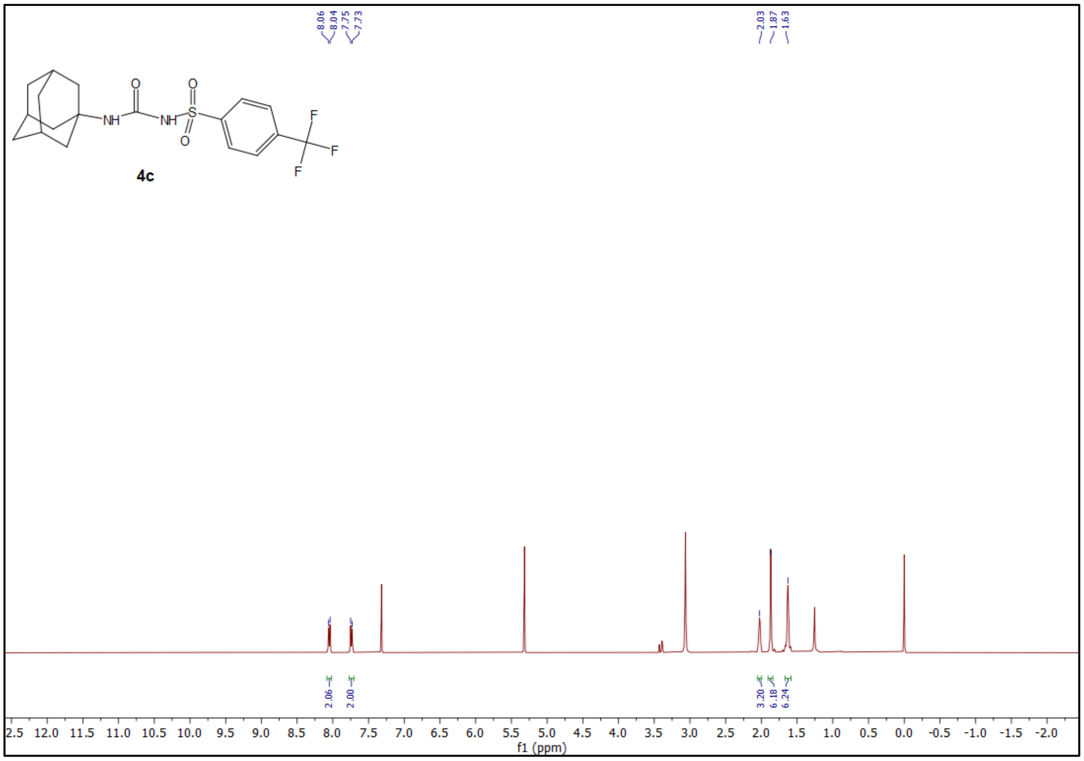

^{13}C NMR of **4c**

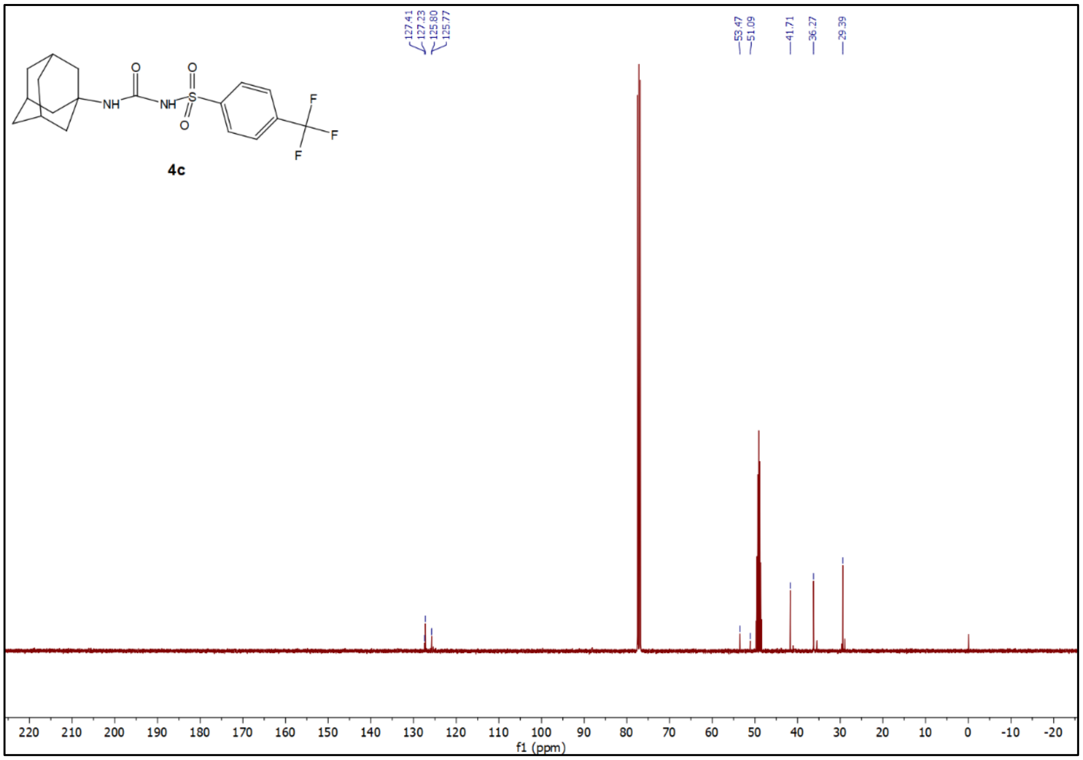

^{1}H NMR of **4d**

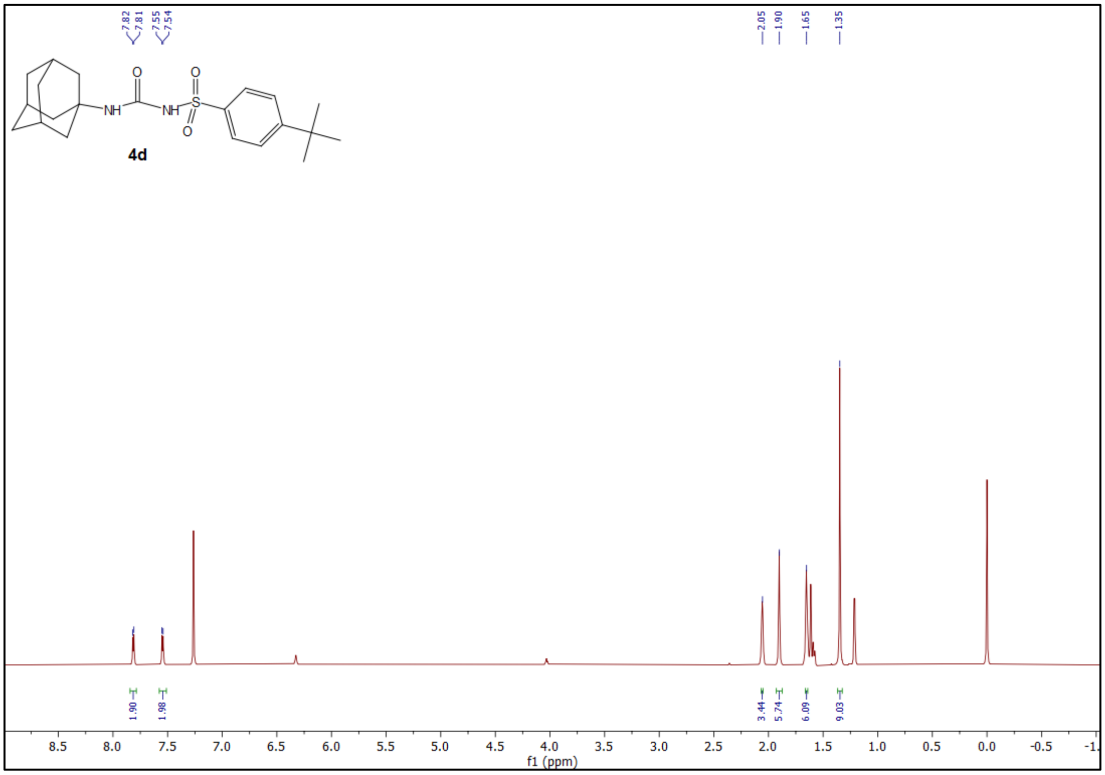

^{13}C NMR of **4d**

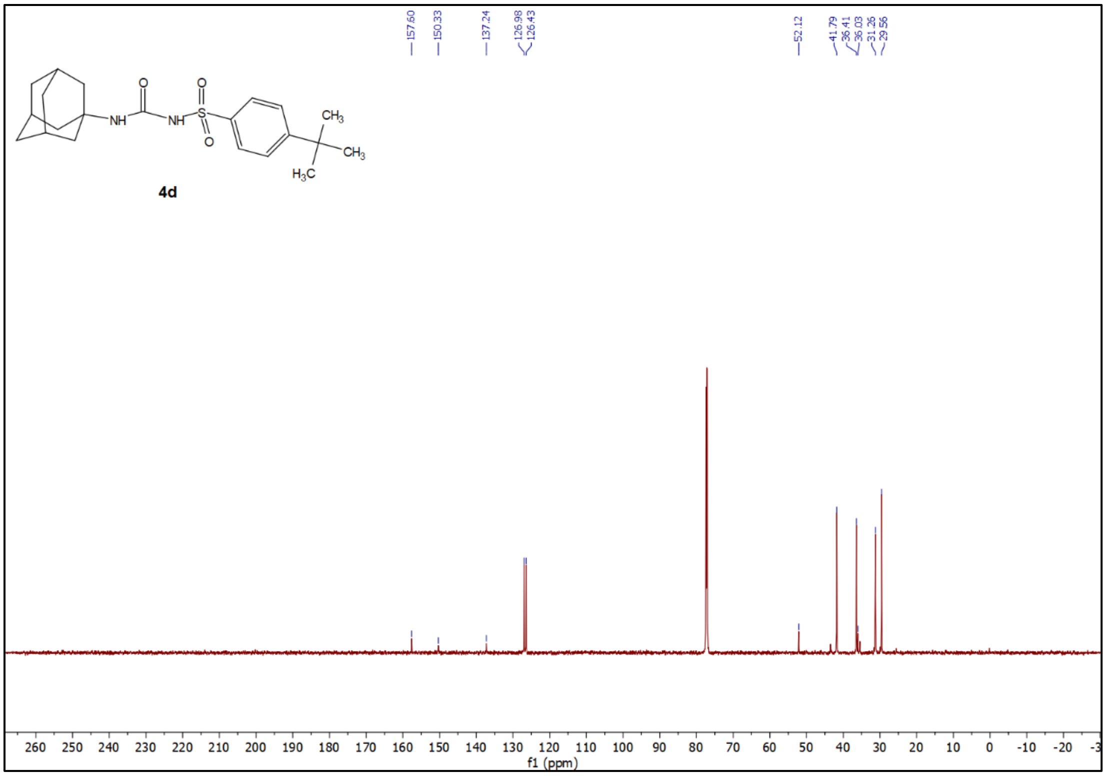

^{1}H NMR of **4e**

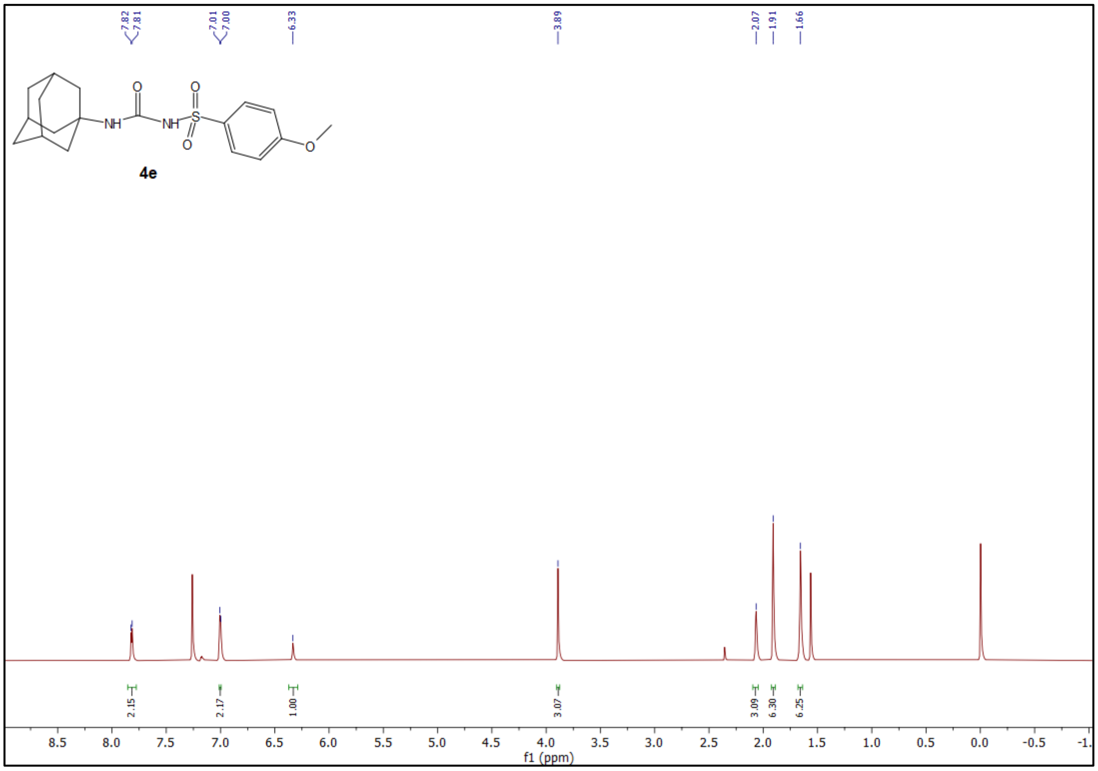

^{13}C NMR of **4e**

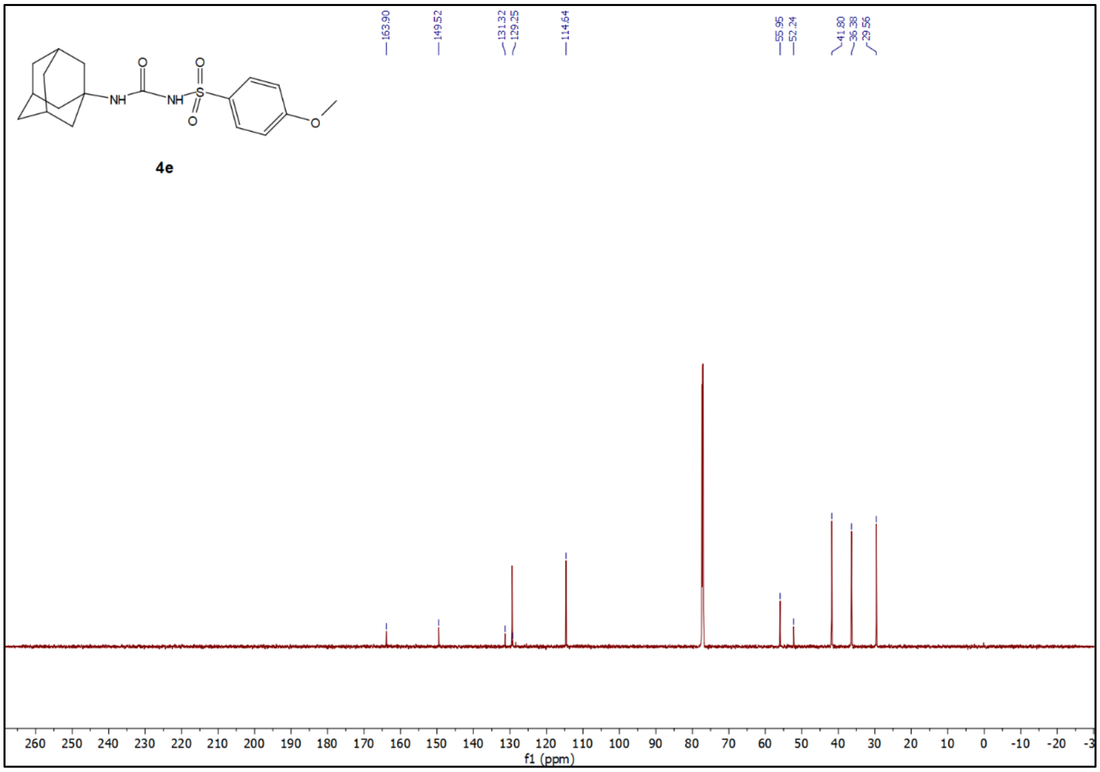

^{1}H NMR of **4f**

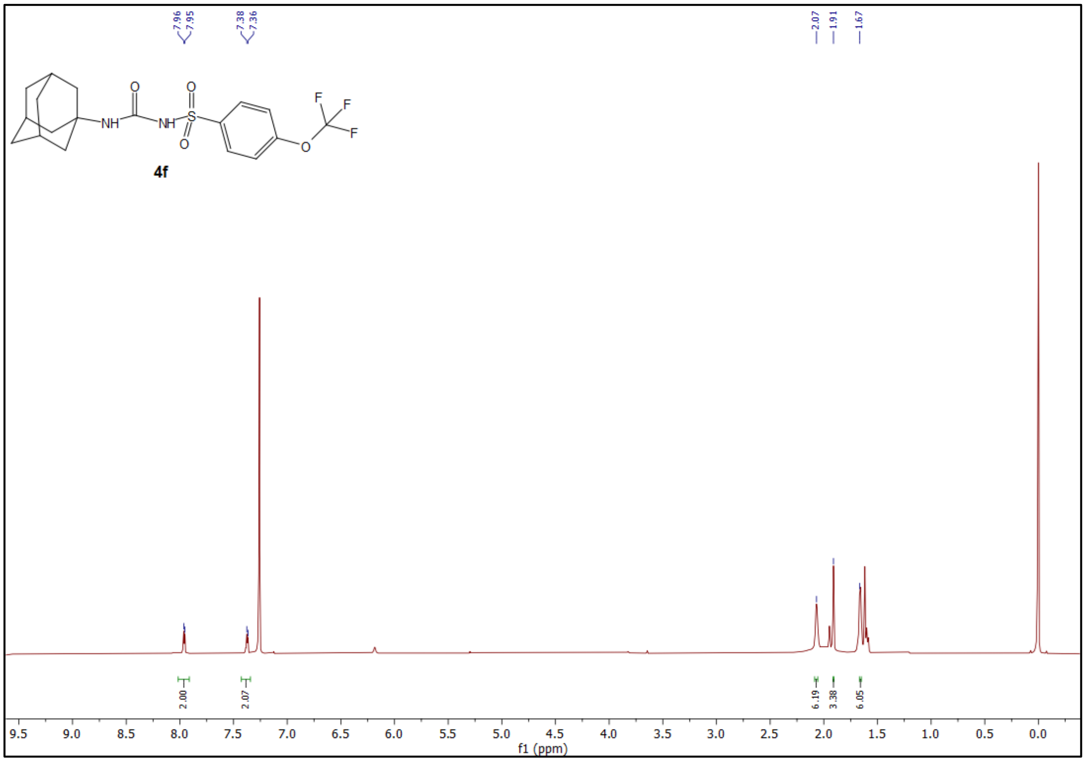

^{13}C NMR of **4f**

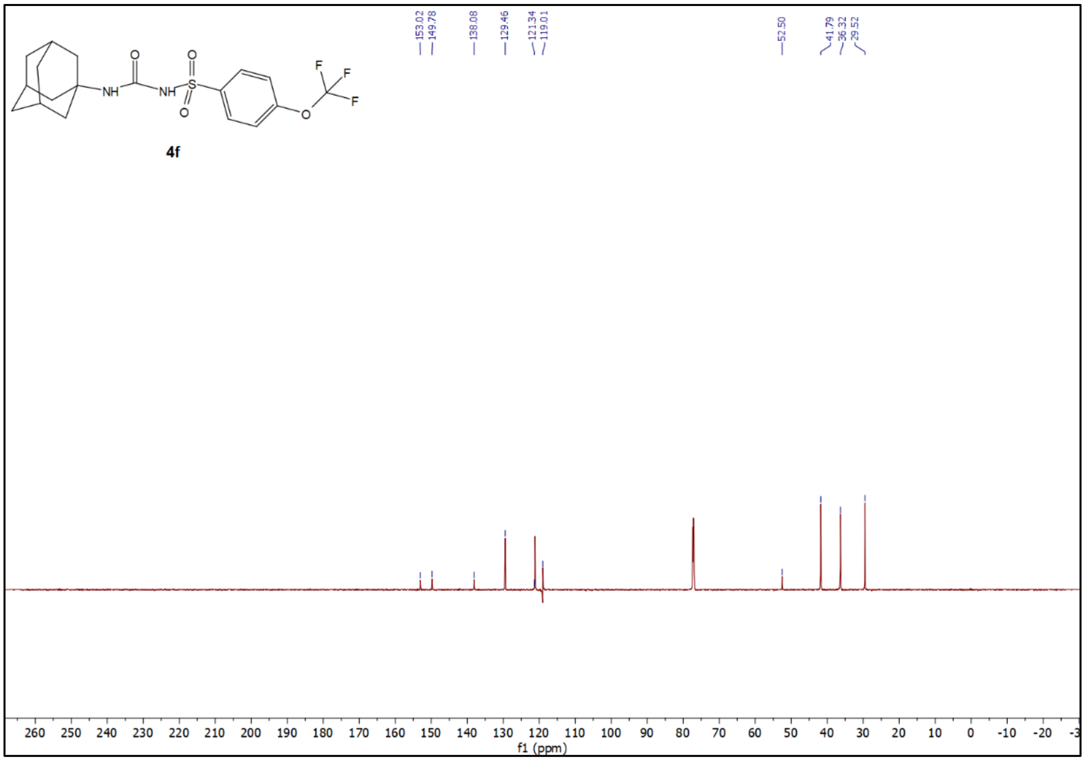

^{1}H NMR of **4g**

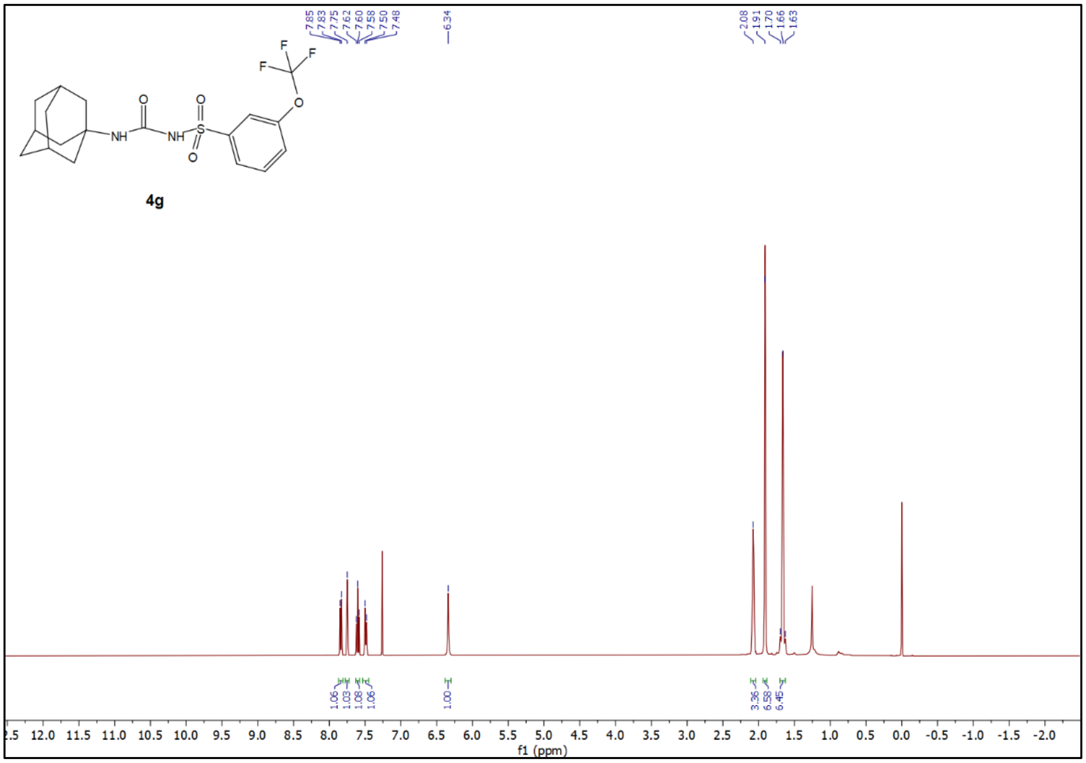

^{13}C NMR of **4g**

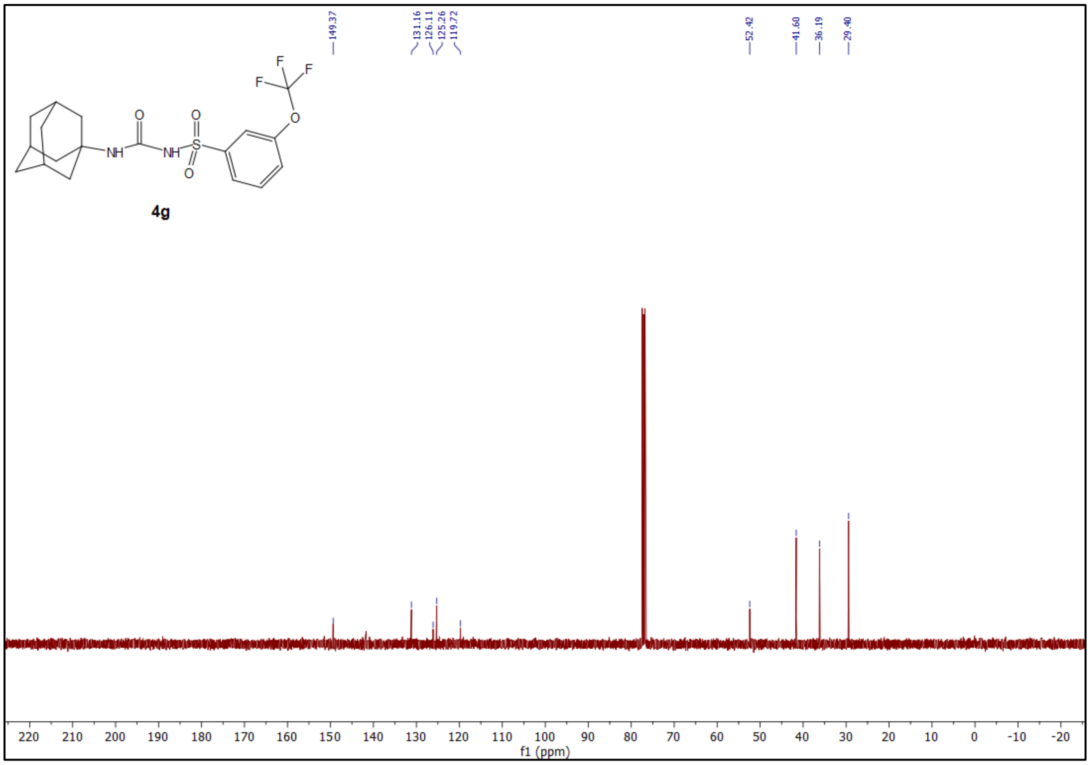

^{1}H NMR of **4h**

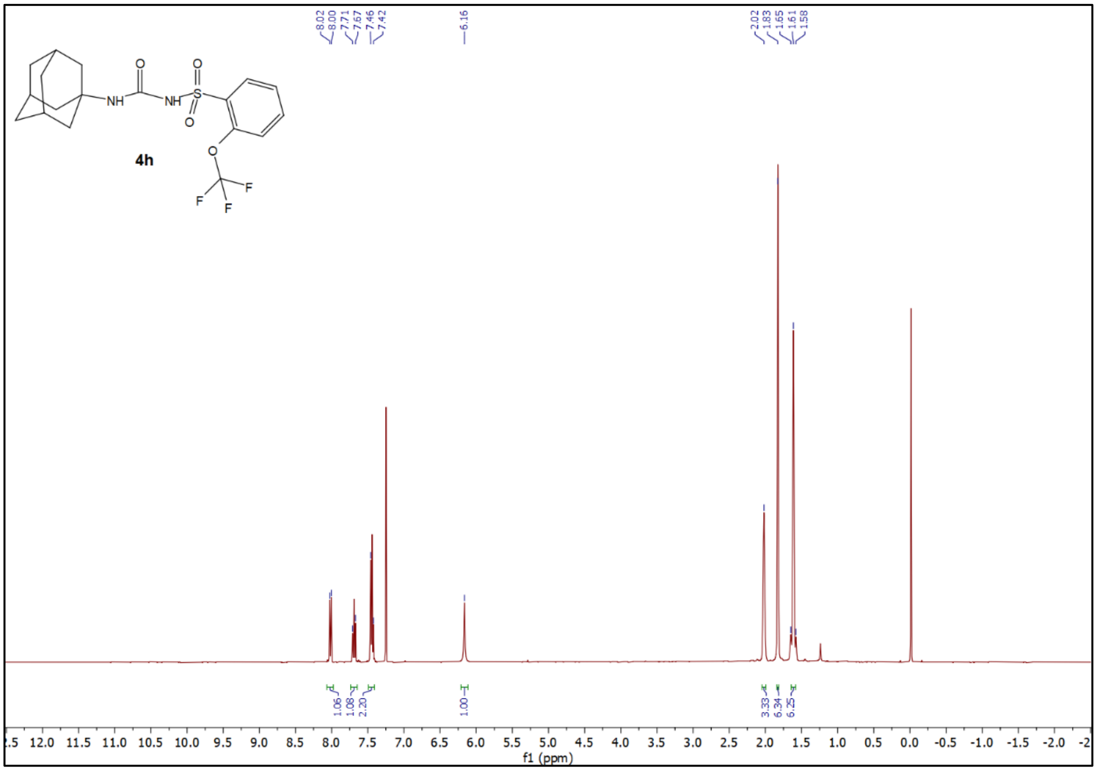

^{13}C NMR of **4h**

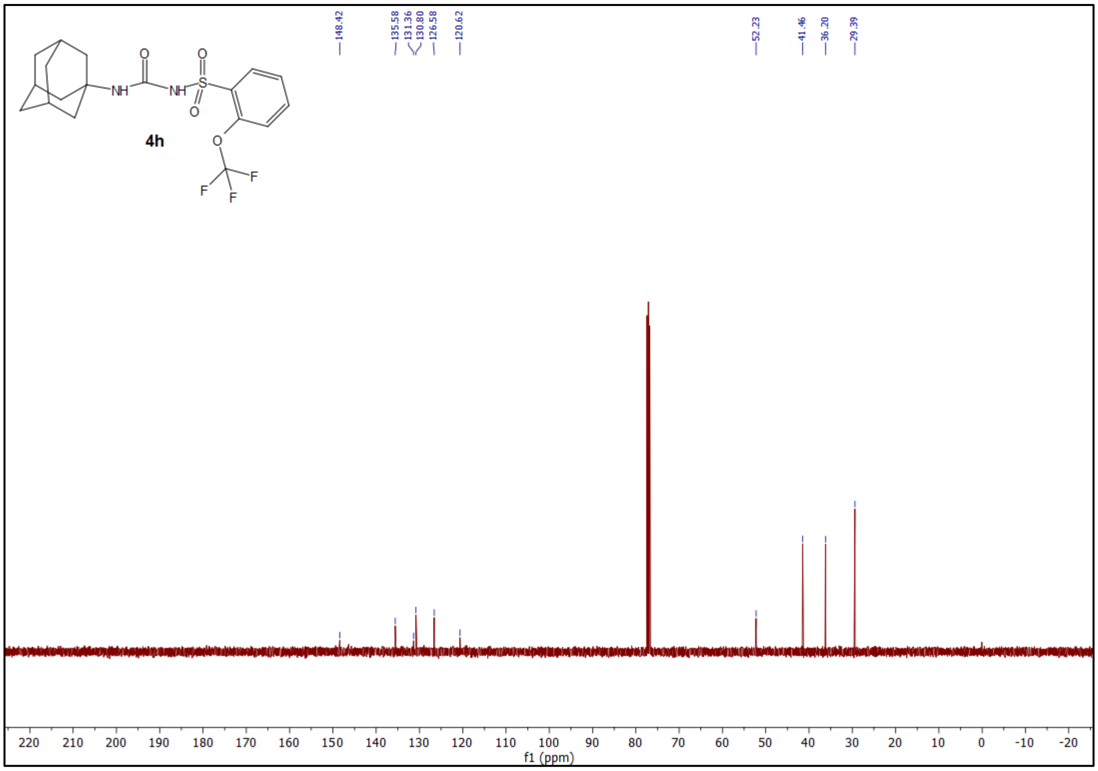

^{1}H NMR of **4i**

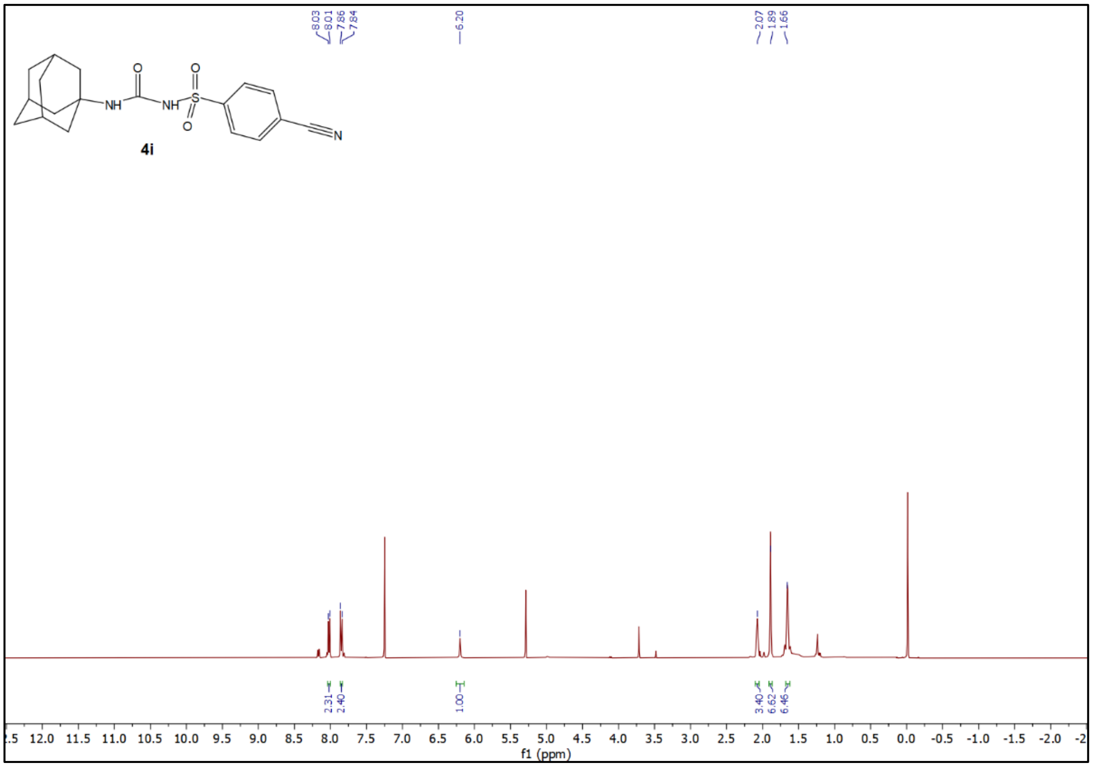

^{13}C NMR of **4i**

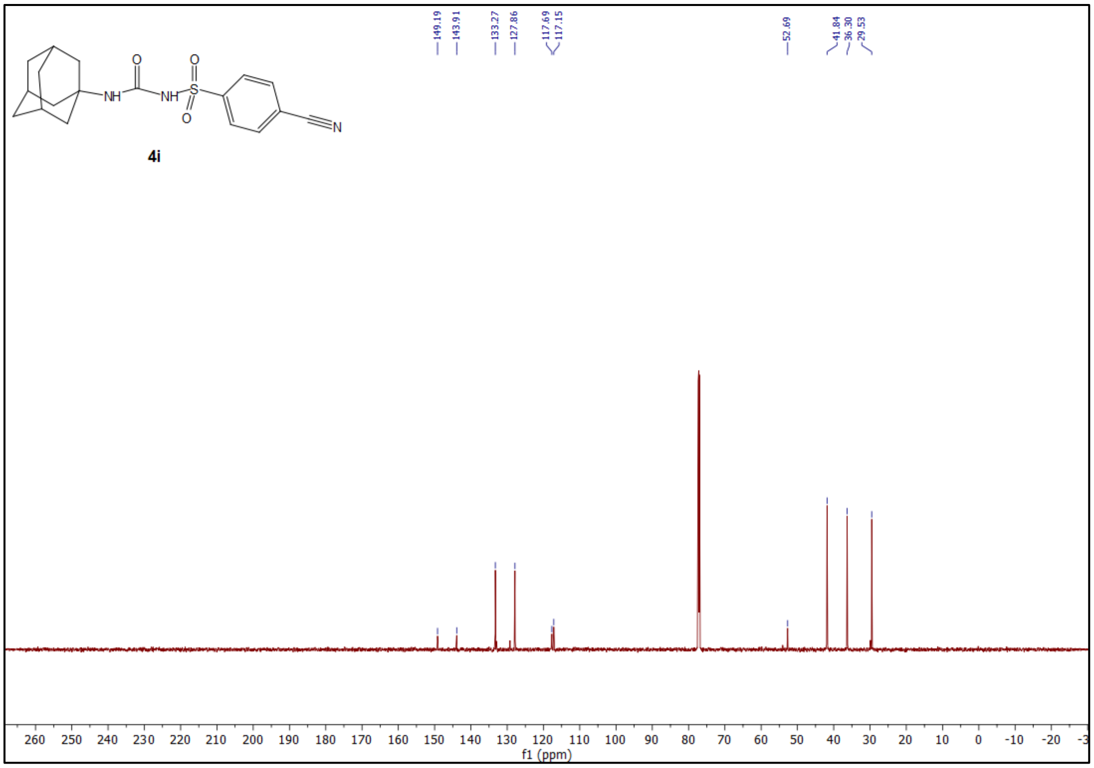

^{1}H NMR of **4j**

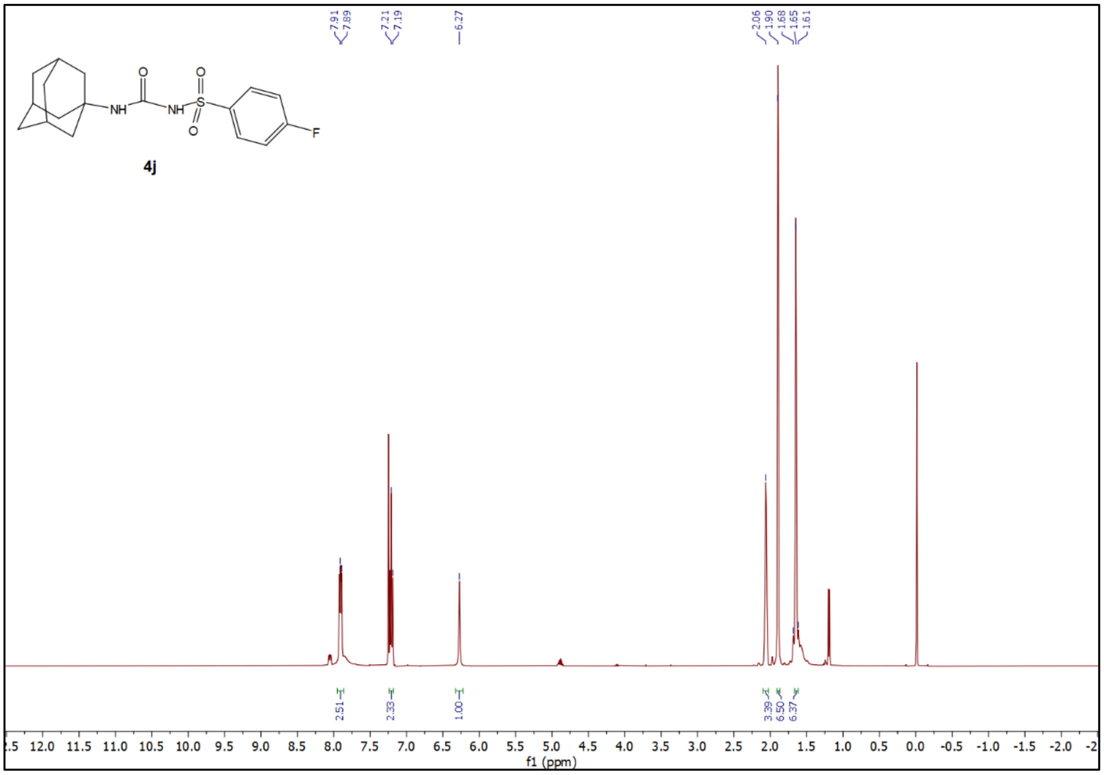

^{13}C NMR of **4j**

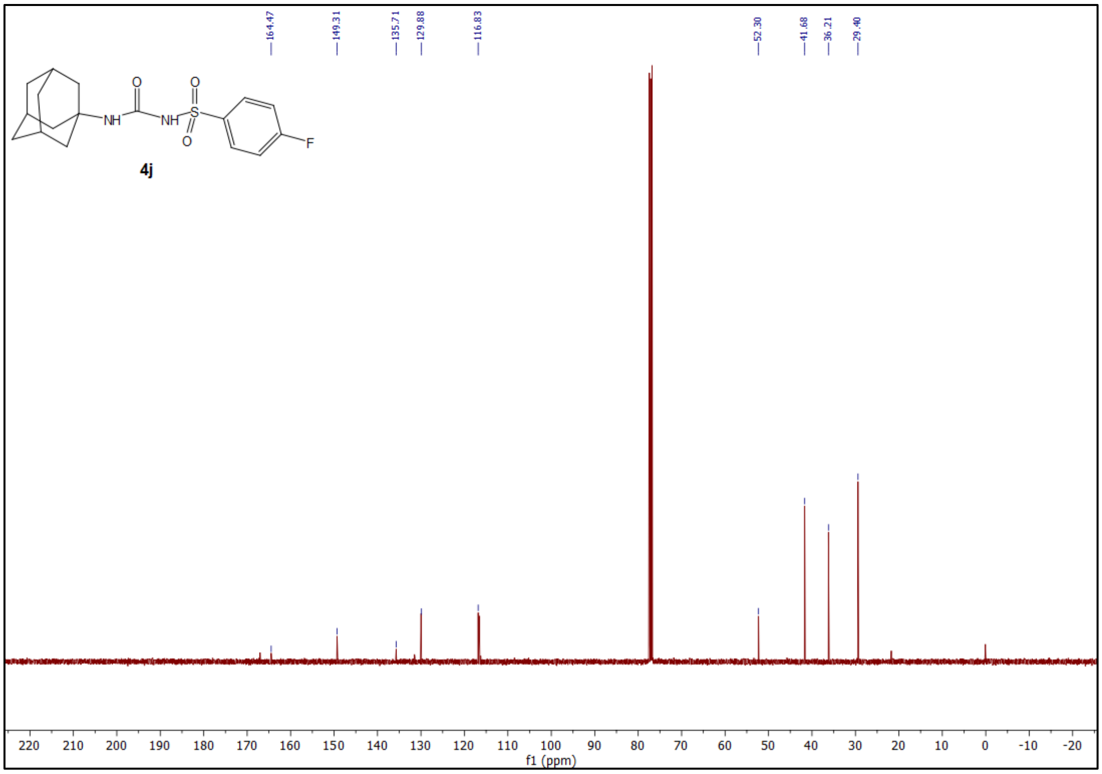

^{1}H NMR of **4k**

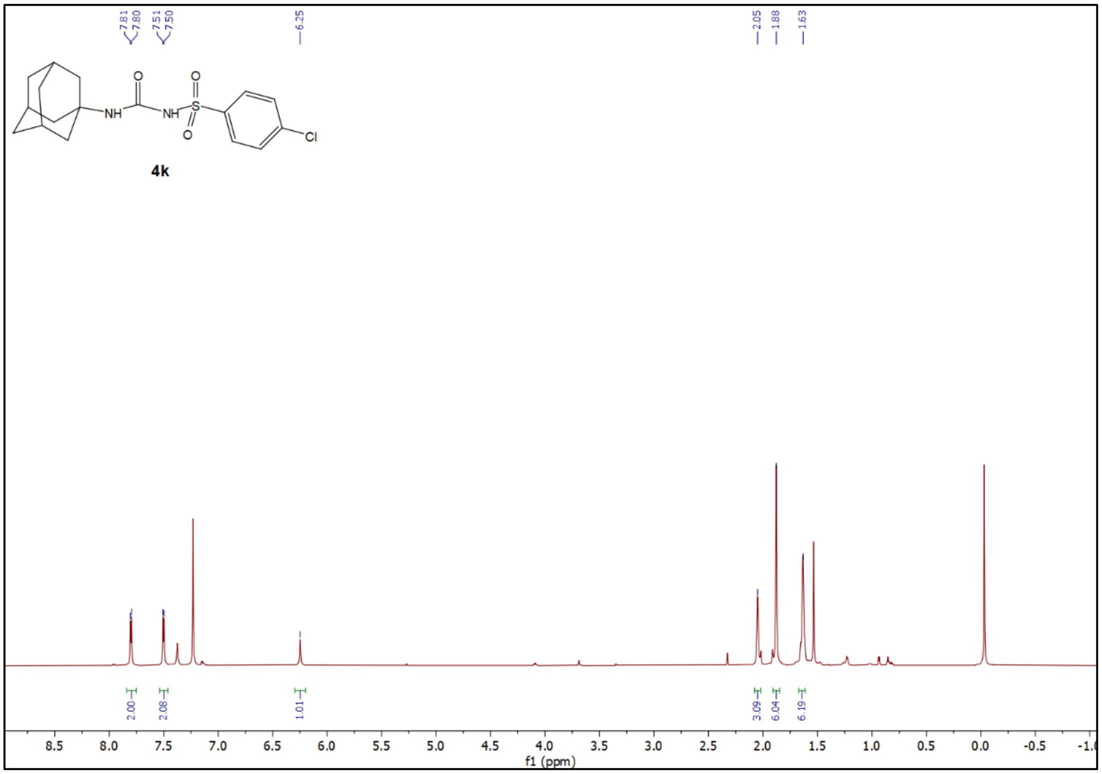

^{13}C NMR of **4k**

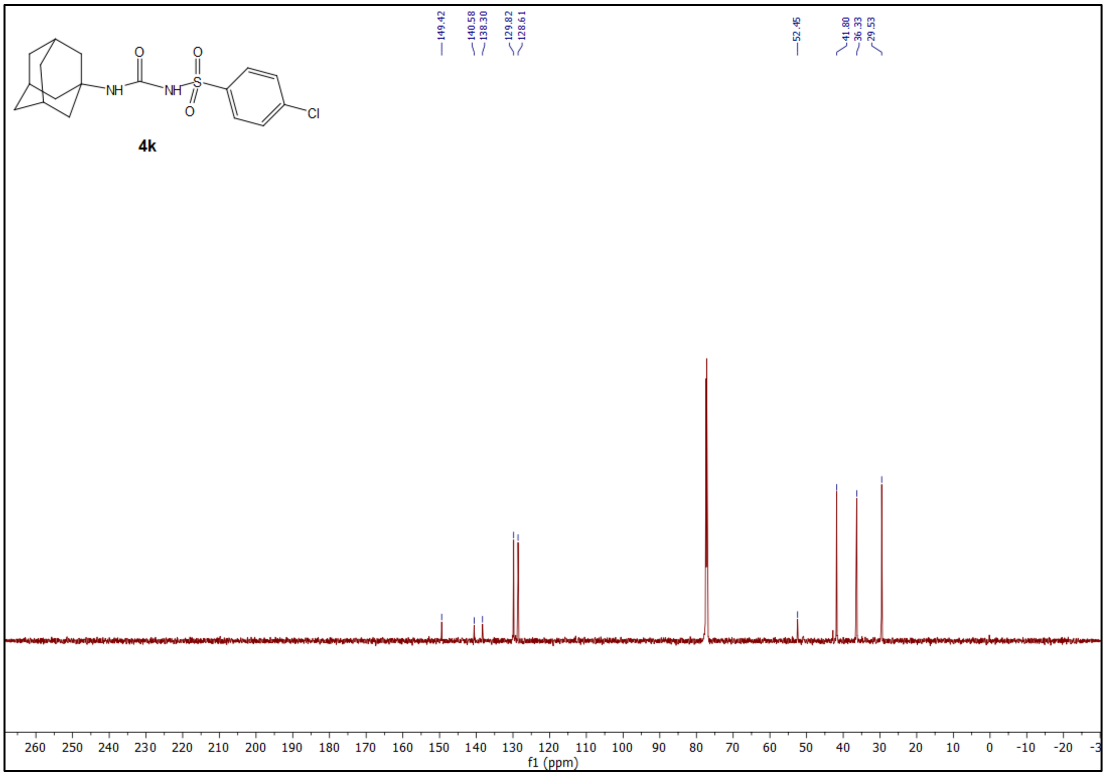

^{1}H NMR of **4l**

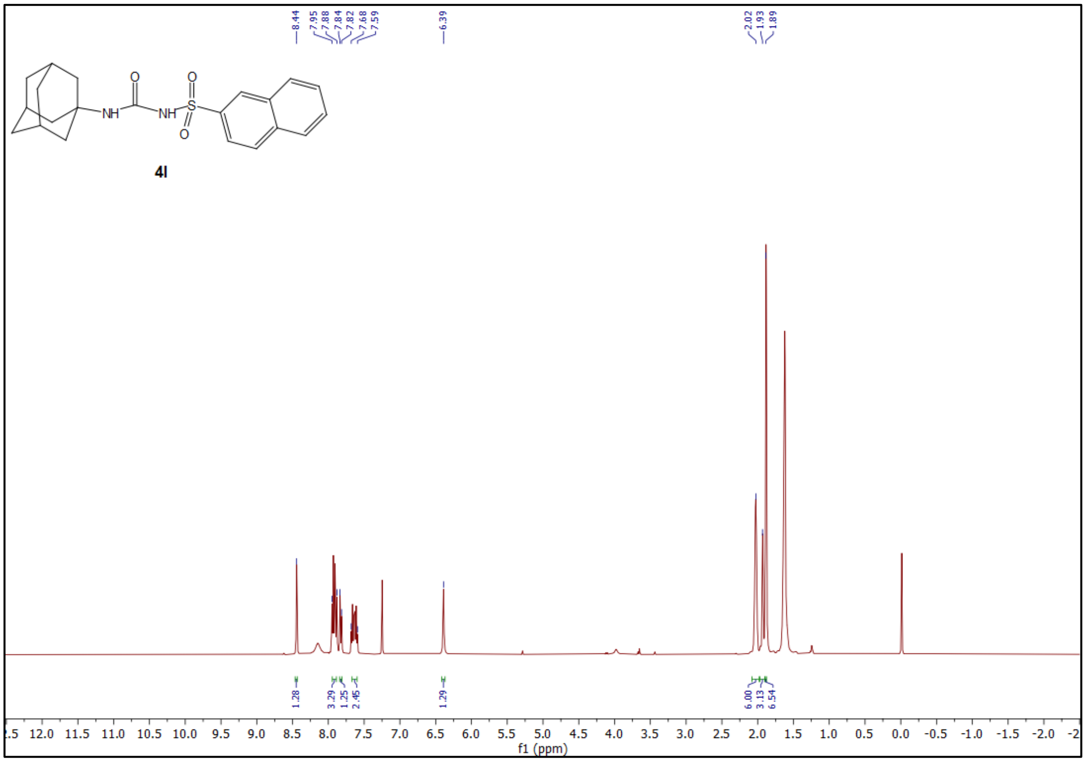

^{13}C NMR of **4l**

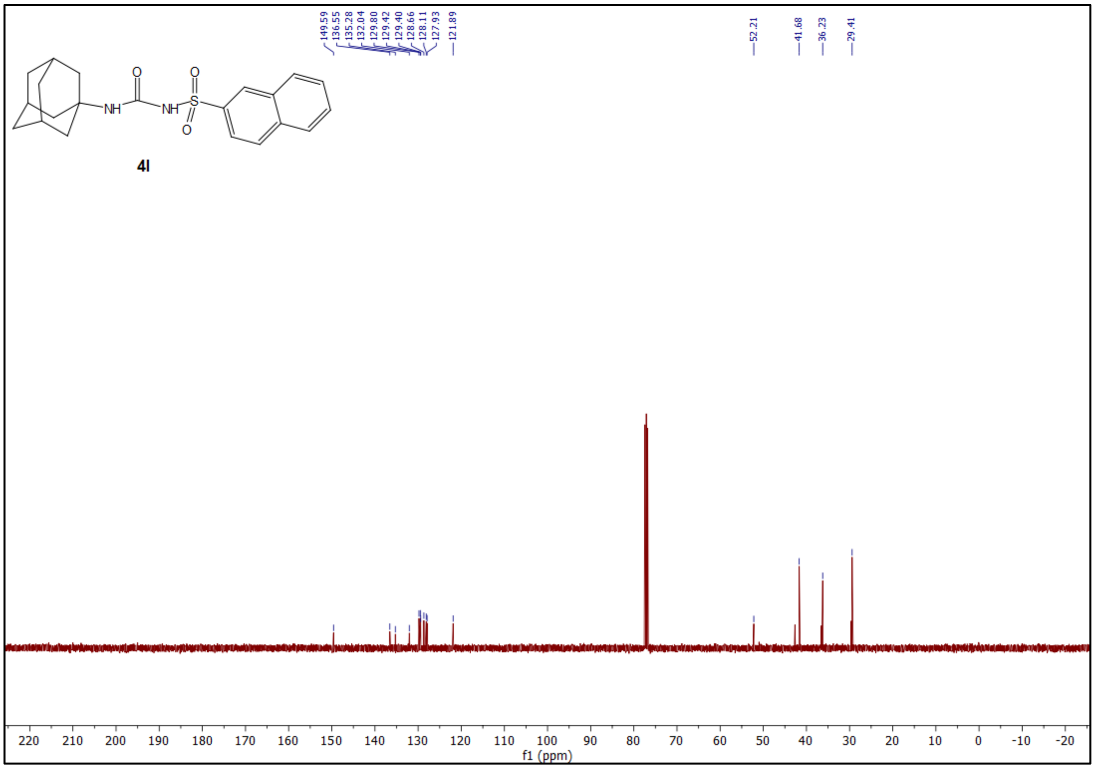
149.59
136.55
135.28
132.04
129.80
129.42
129.40
128.66
128.11
127.93
121.89
52.21
41.68
36.23
29.41
NH
NH
O
O
O
S
4l
220 210 200 190 180 170 160 150 140 130 120 110 100 90 80 70 60 50 40 30 20 10 0 -10 -20
f1 (ppm)

^{1}H NMR of **6a**

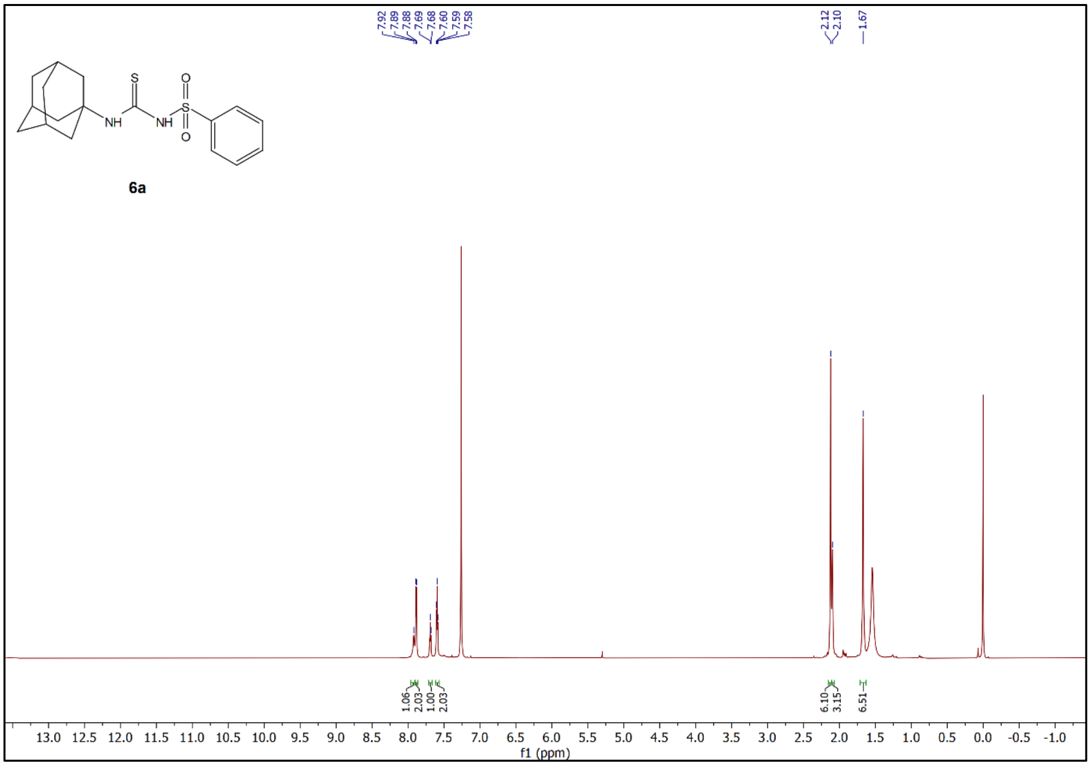
7.92
7.89
7.88
7.69
7.68
7.60
7.59
7.58
2.12
2.10
1.67
S
O
O
NH
NH
S
6a
1.06
2.03
1.00
2.03
6.10
3.15
6.51
13.0 12.5 12.0 11.5 11.0 10.5 10.0 9.5 9.0 8.5 8.0 7.5 7.0 6.5 6.0 5.5 5.0 4.5 4.0 3.5 3.0 2.5 2.0 1.5 1.0 0.5 0.0 -0.5 -1.0
f1 (ppm)

^{13}C NMR of **6a**

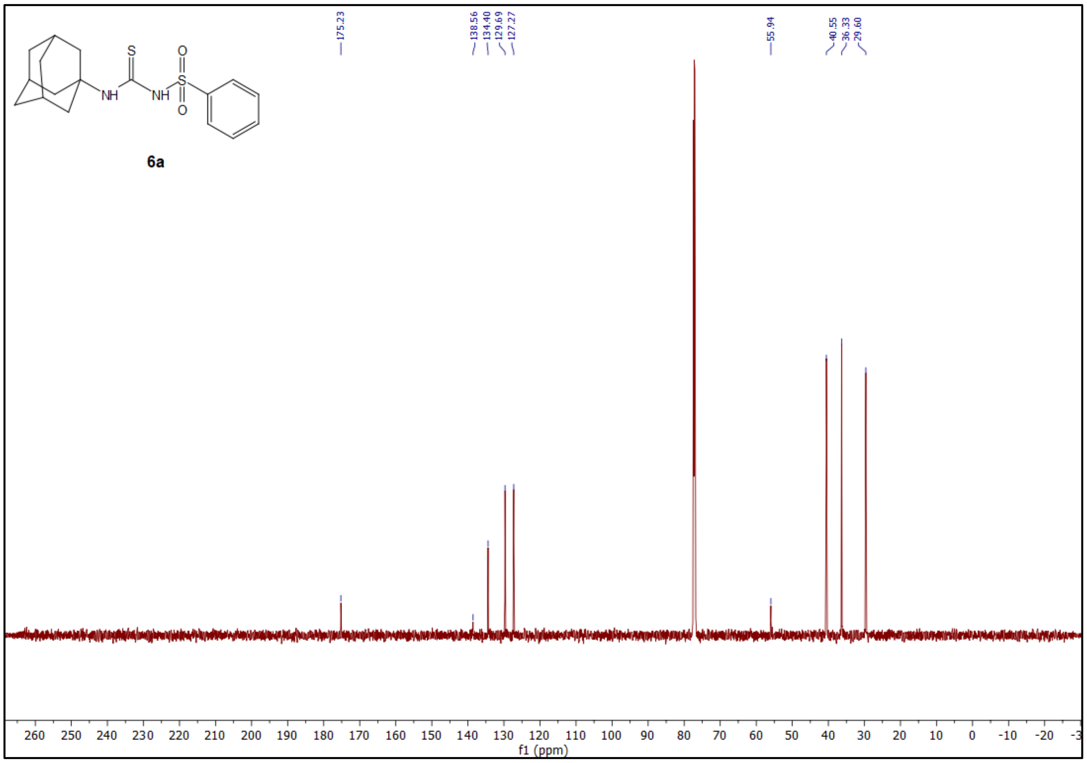

^{1}H NMR of **6b**

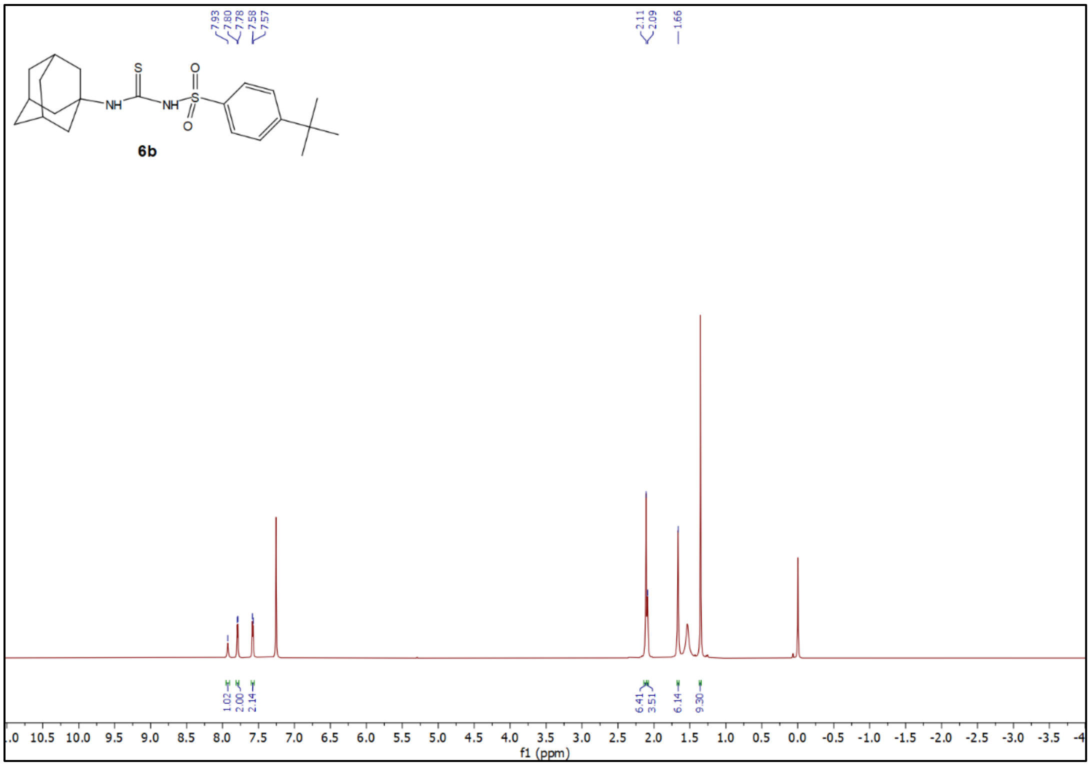

^{13}C NMR of **6b**

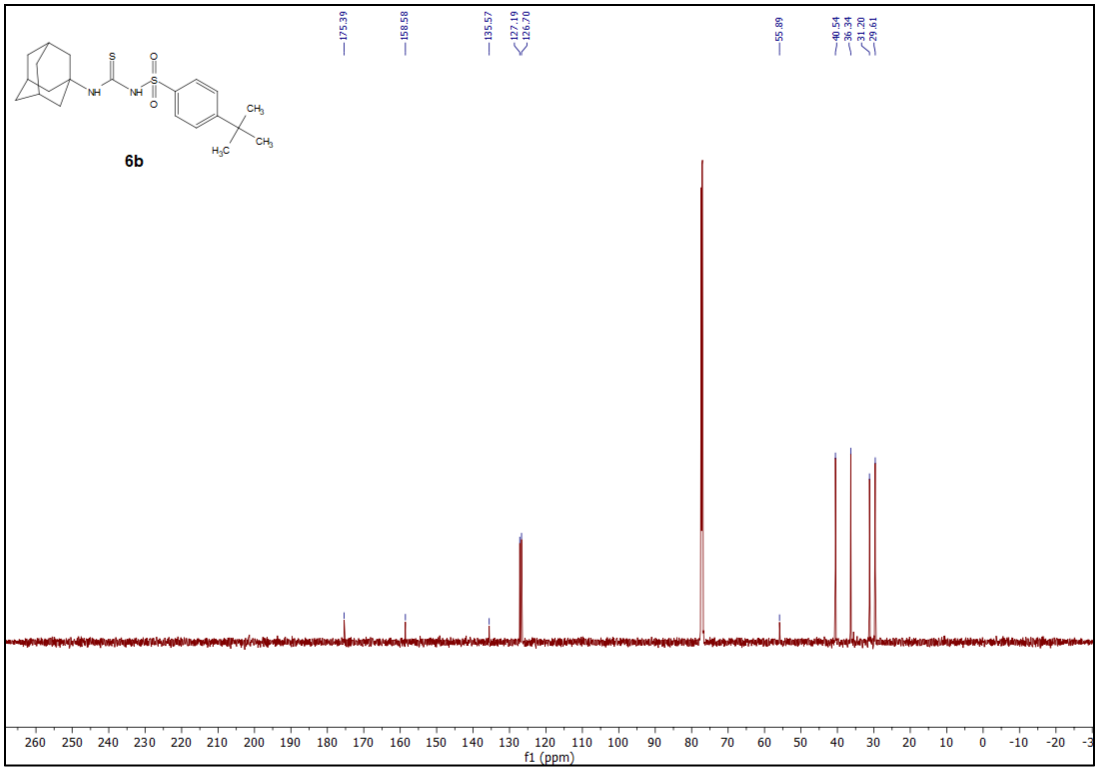

^{1}H NMR of **6c**

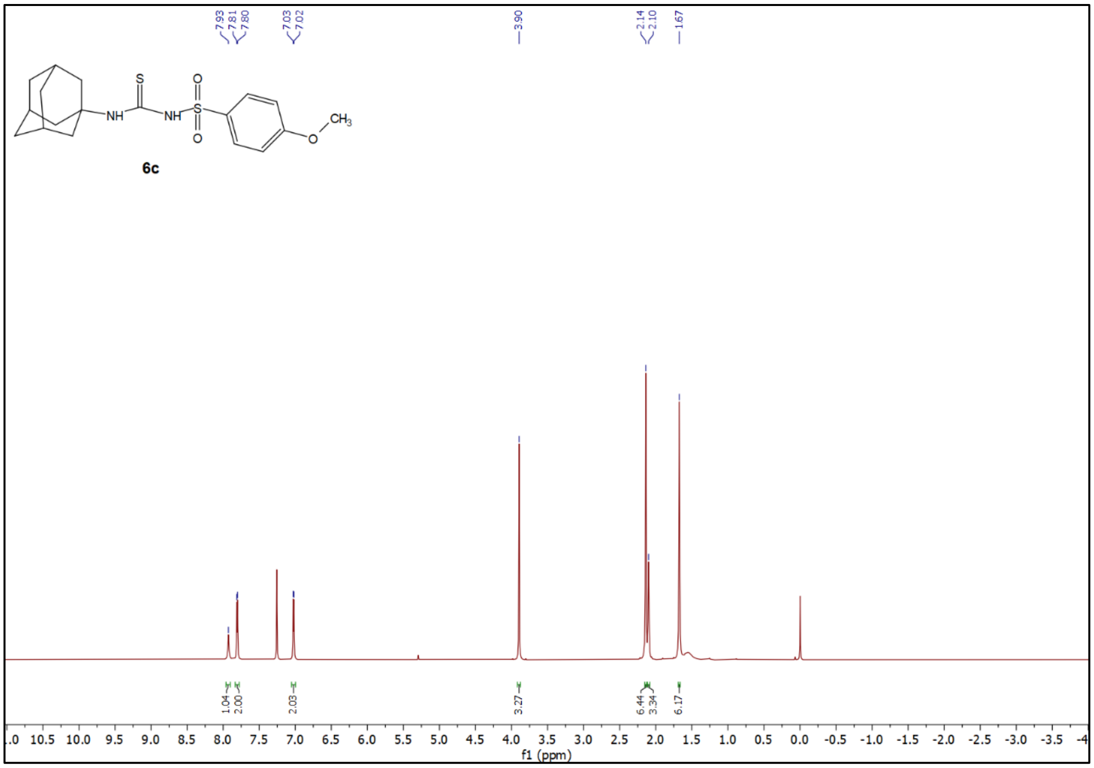

^{13}C NMR of **6c**

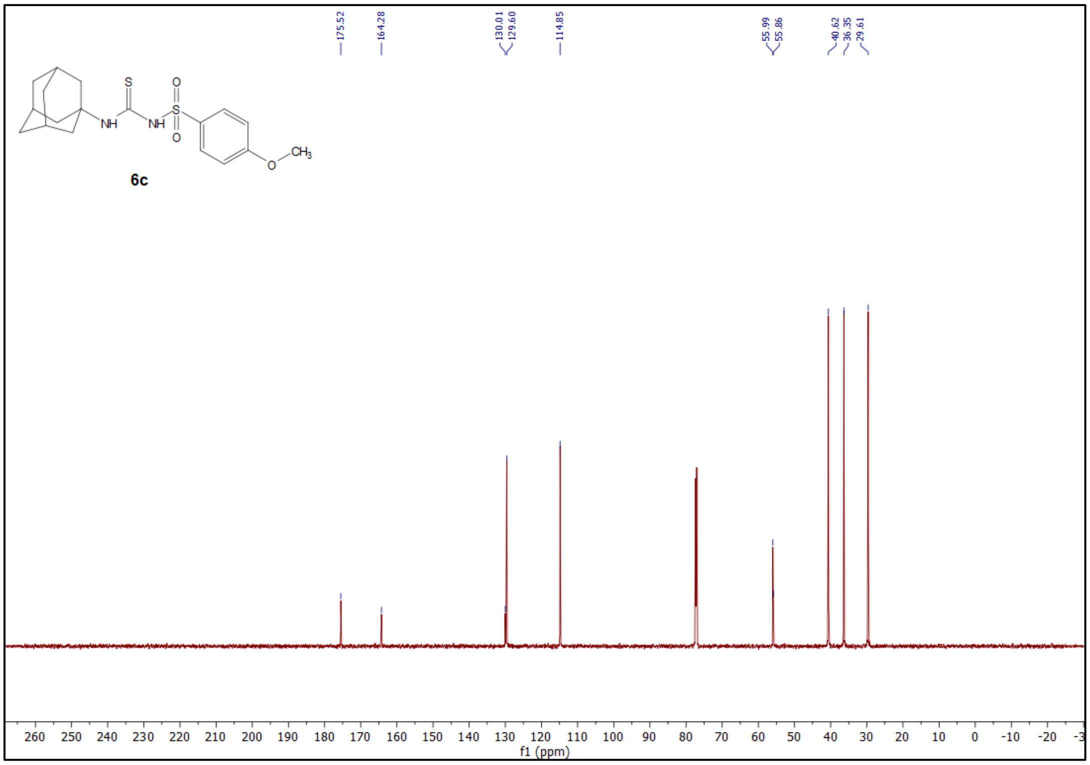

^{1}H NMR of **6d**

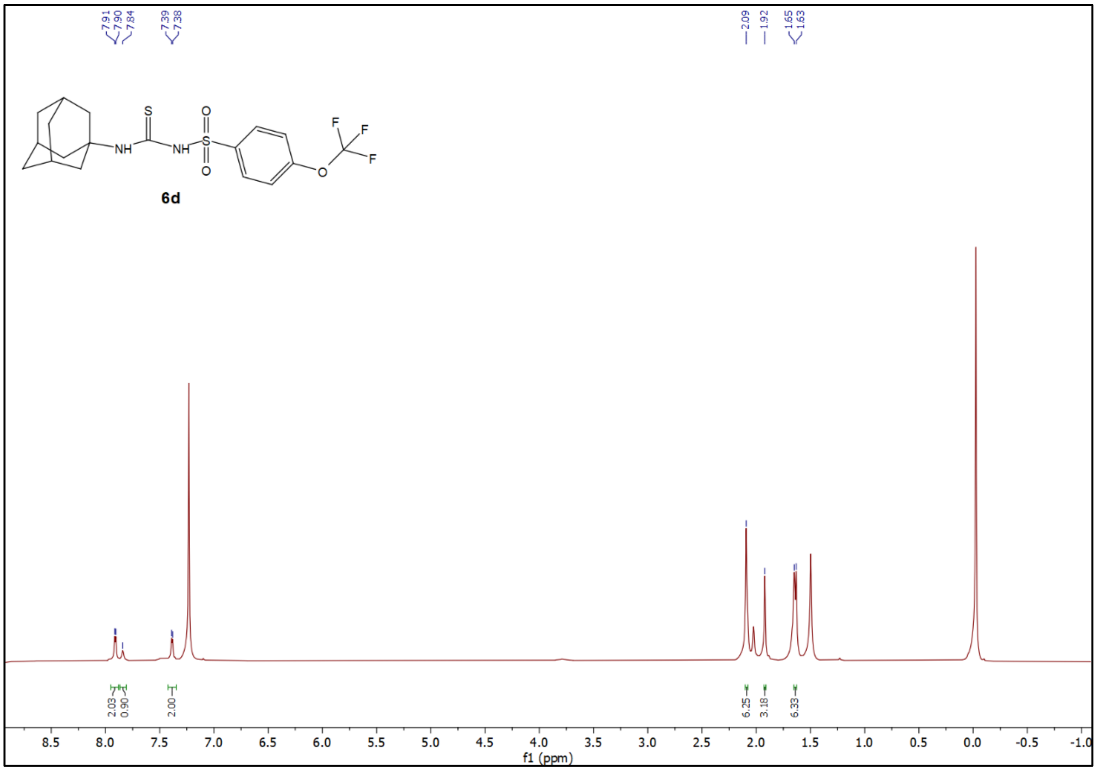

^{13}C NMR of **6d**

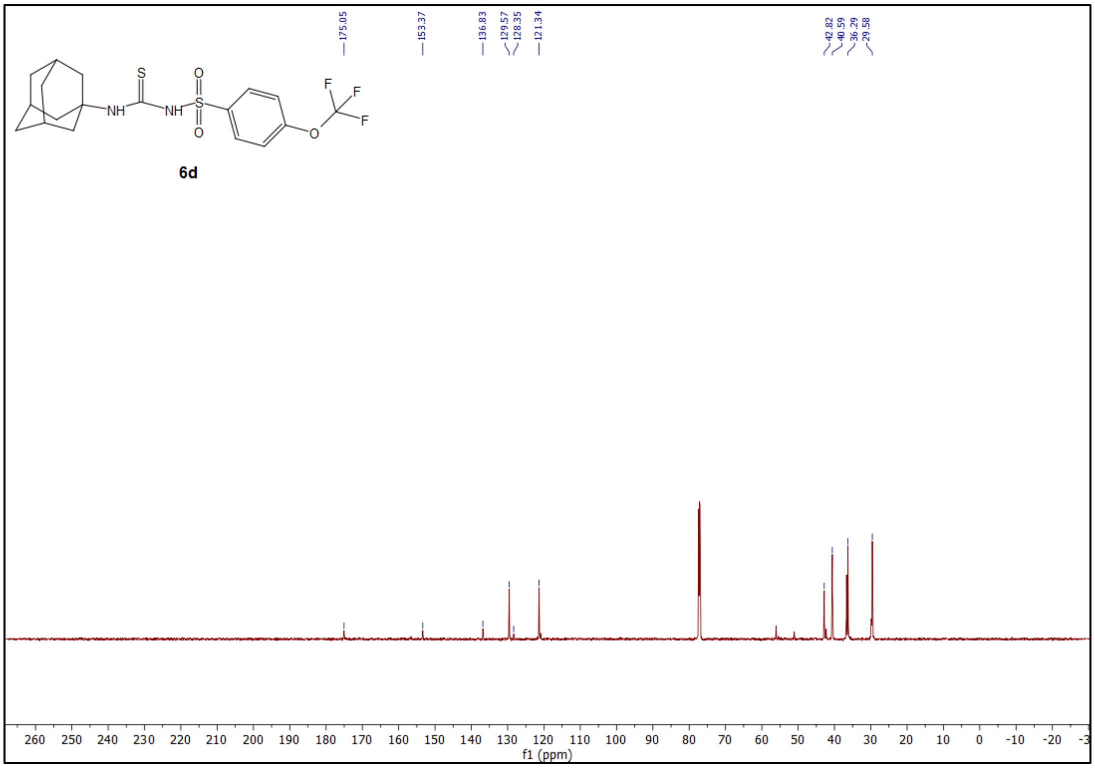

^{1}H NMR of **7**

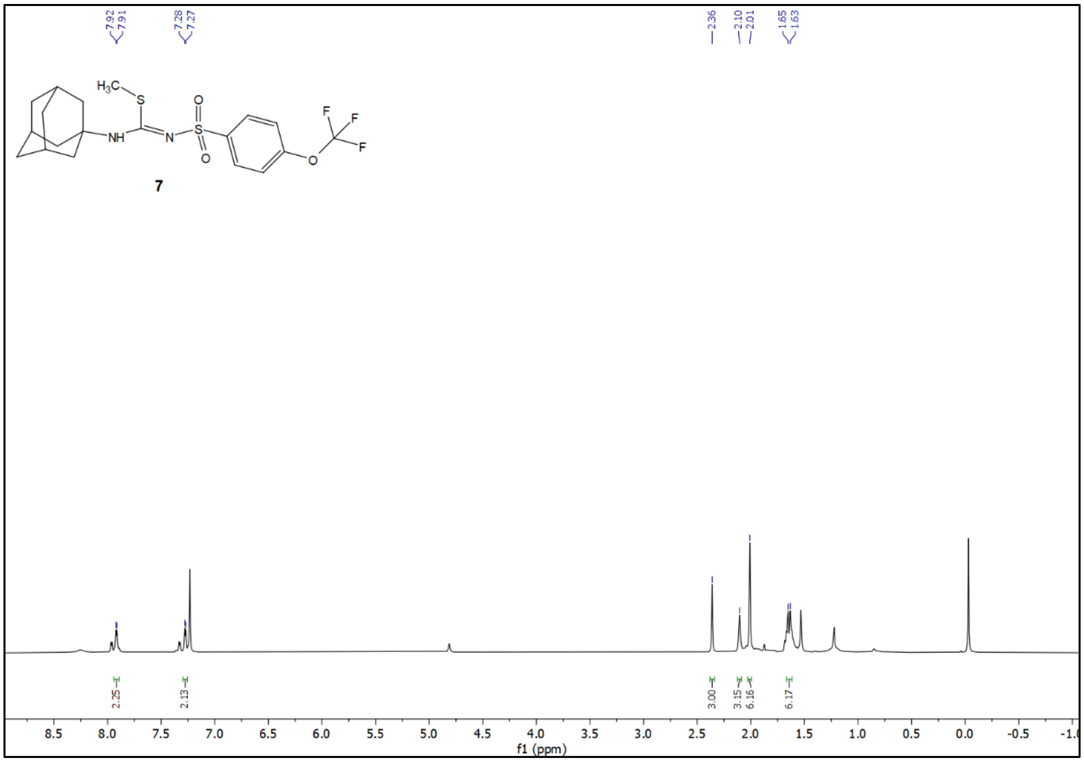

^{13}C NMR of **7**

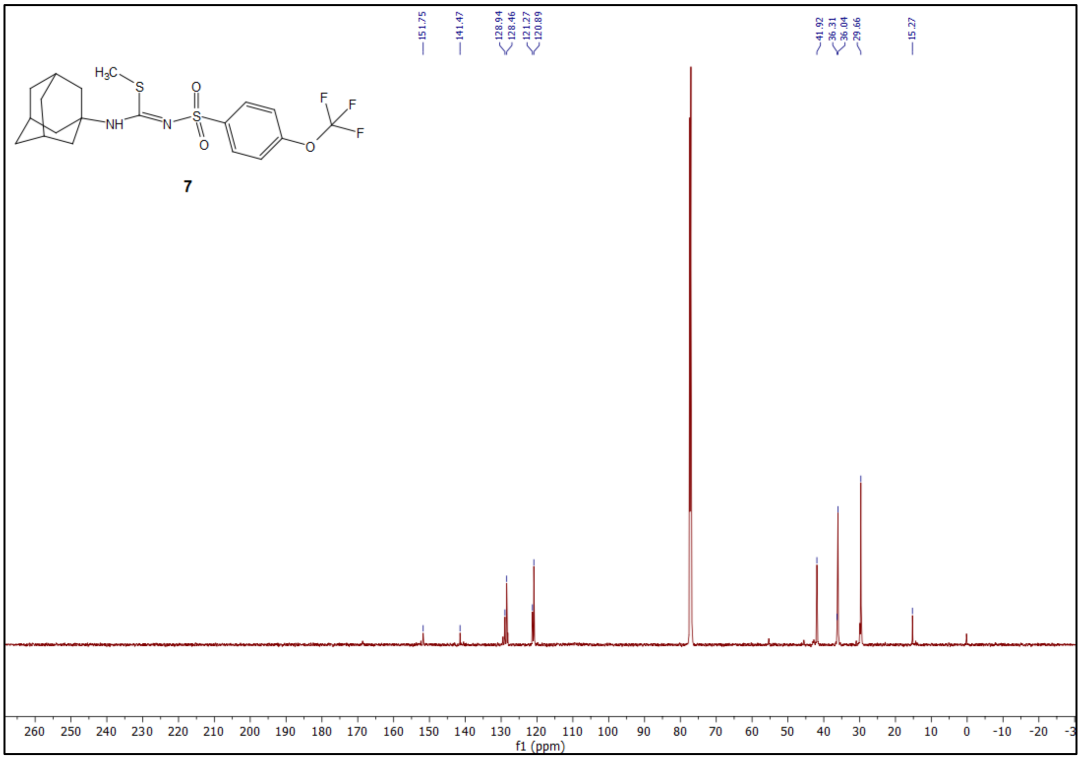

^{1}H NMR of **8a**

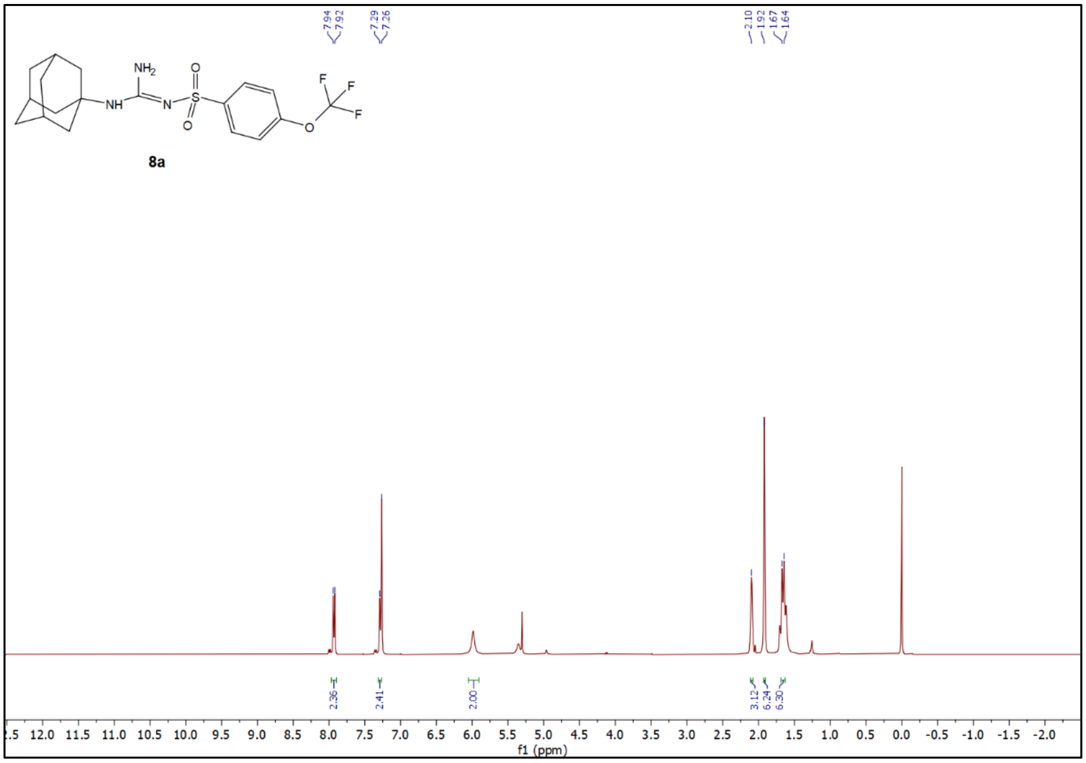

^{13}C NMR of **8a**

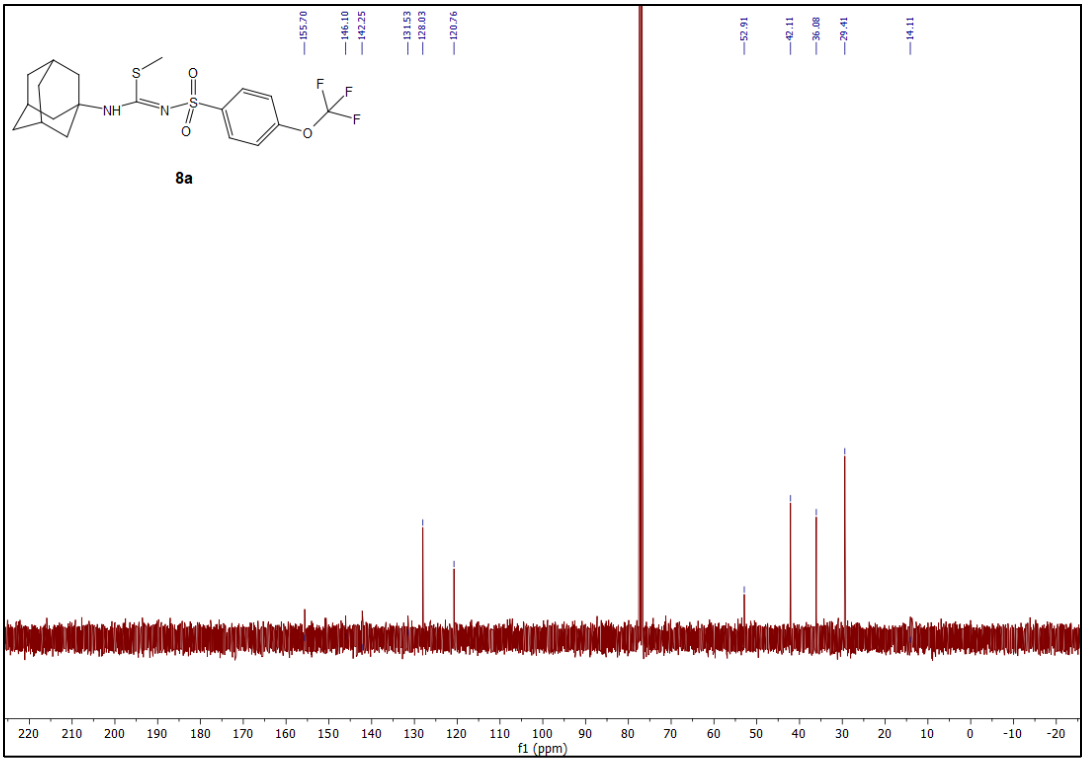

^{1}H NMR of **8b**

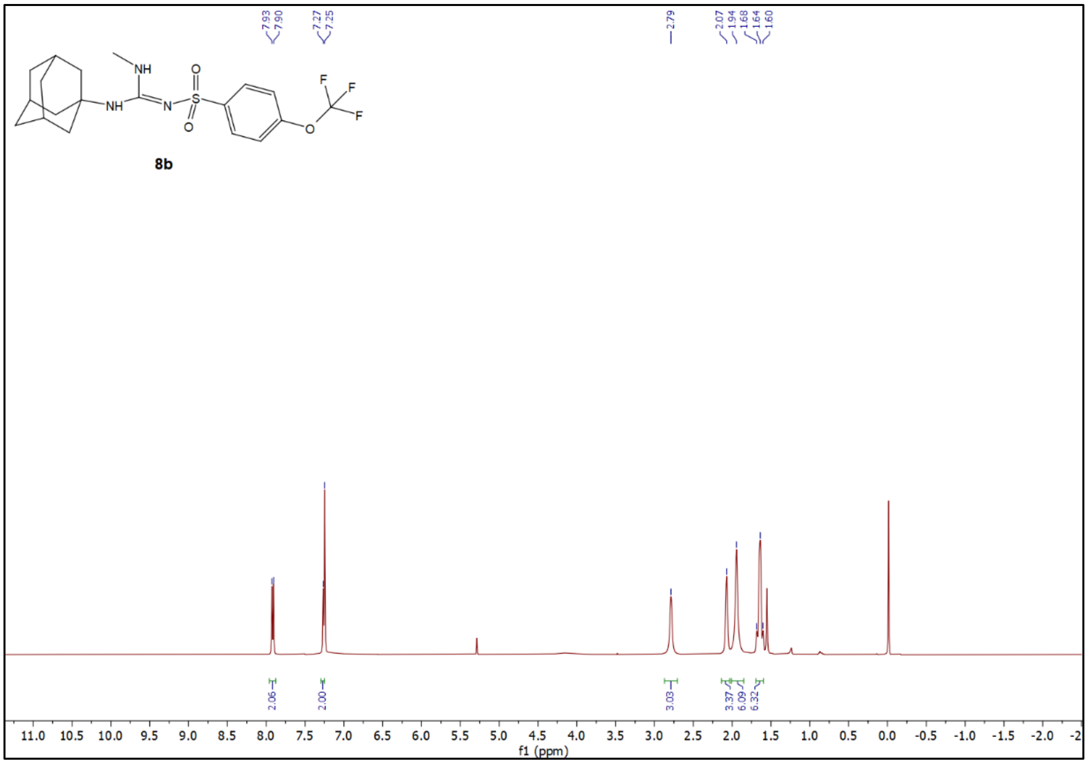

^{13}C NMR of **8b**

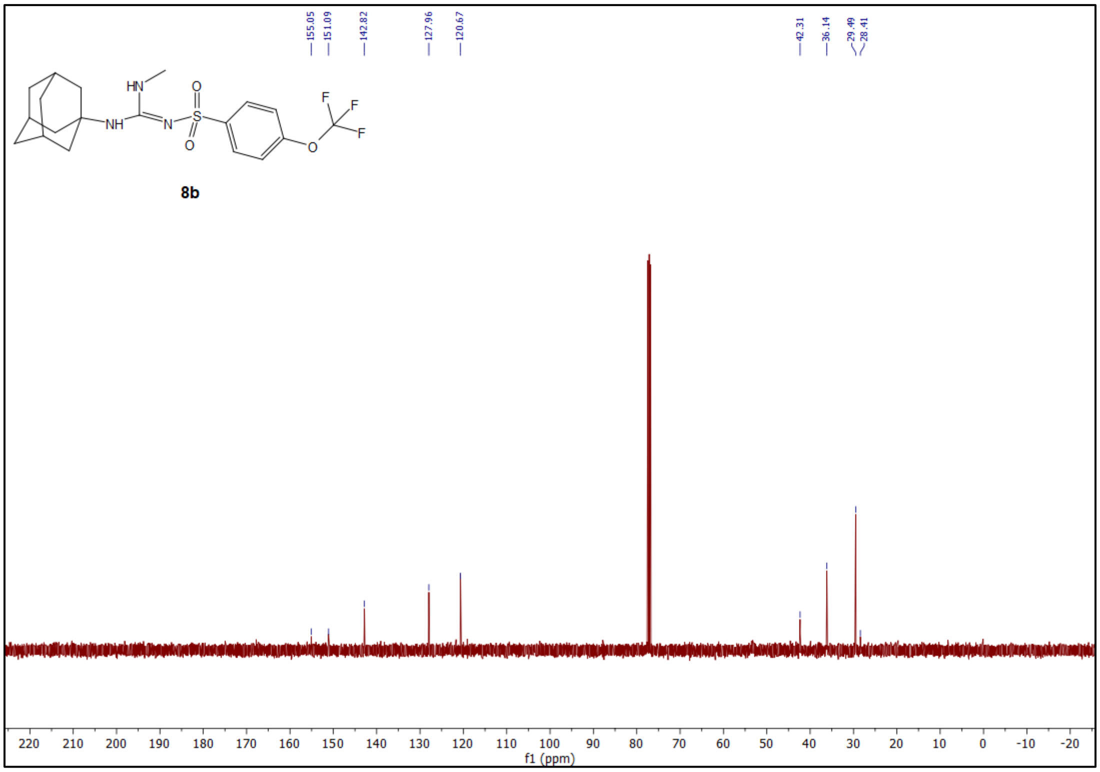

^{1}H NMR of **9a**

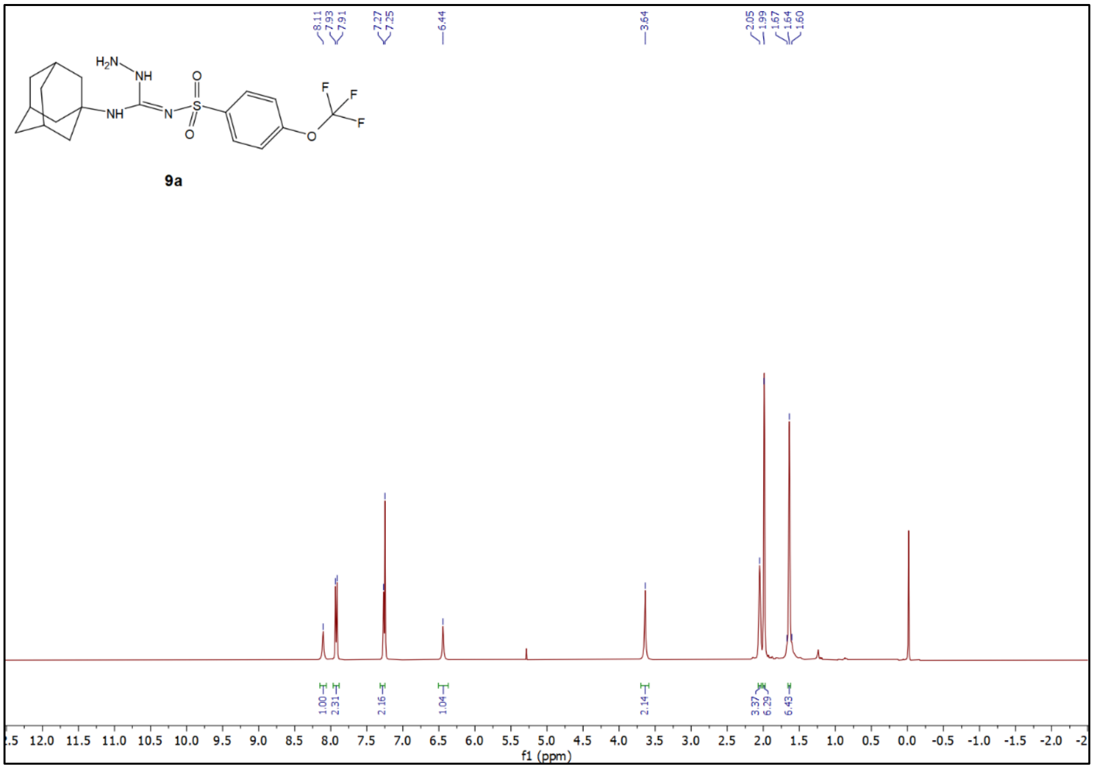

^{13}C NMR of **9a**

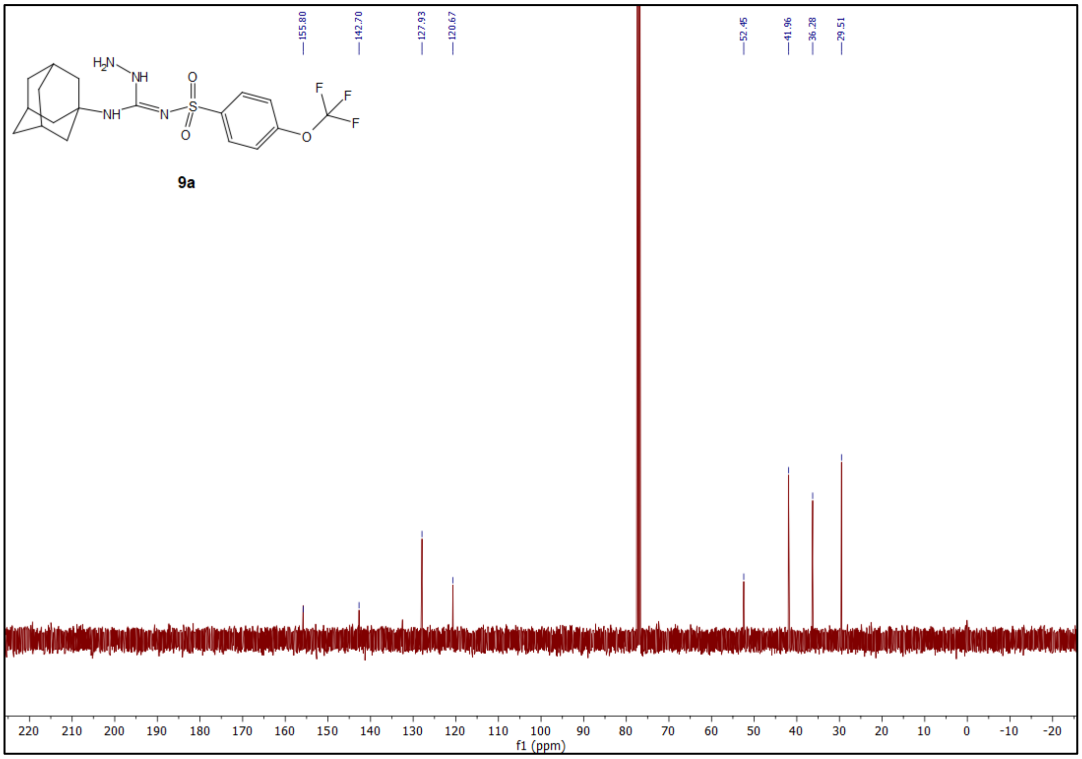

^{1}H NMR of **9b**

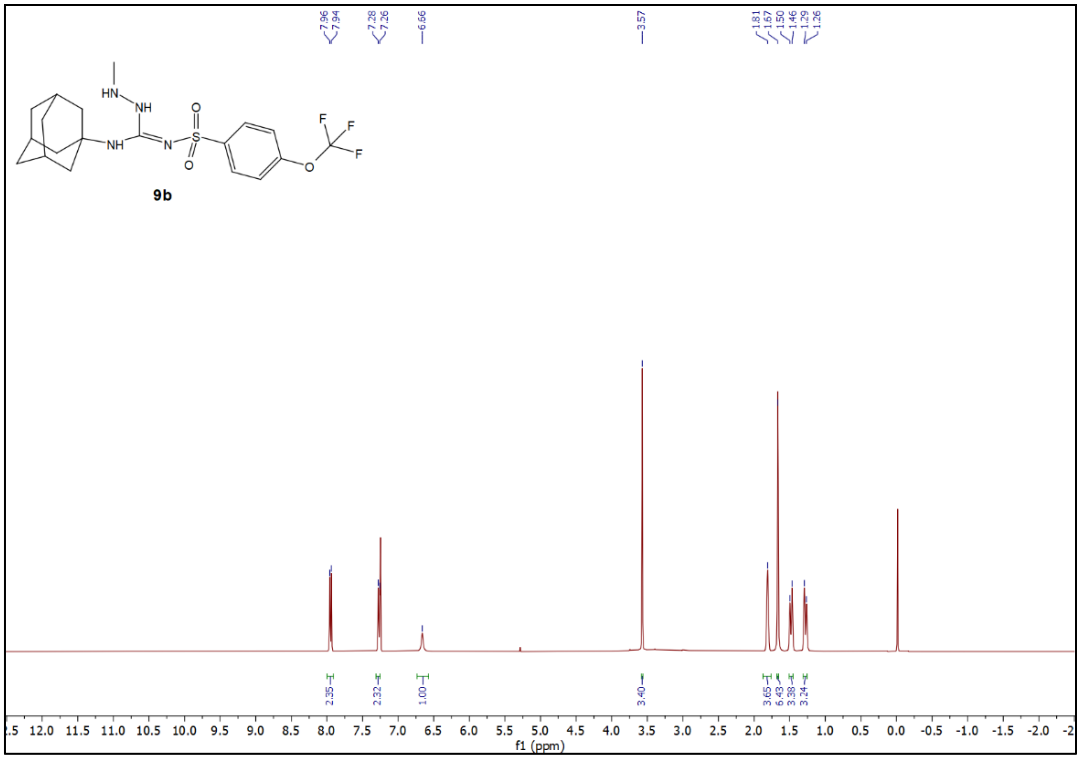

^{13}C NMR of **9b**

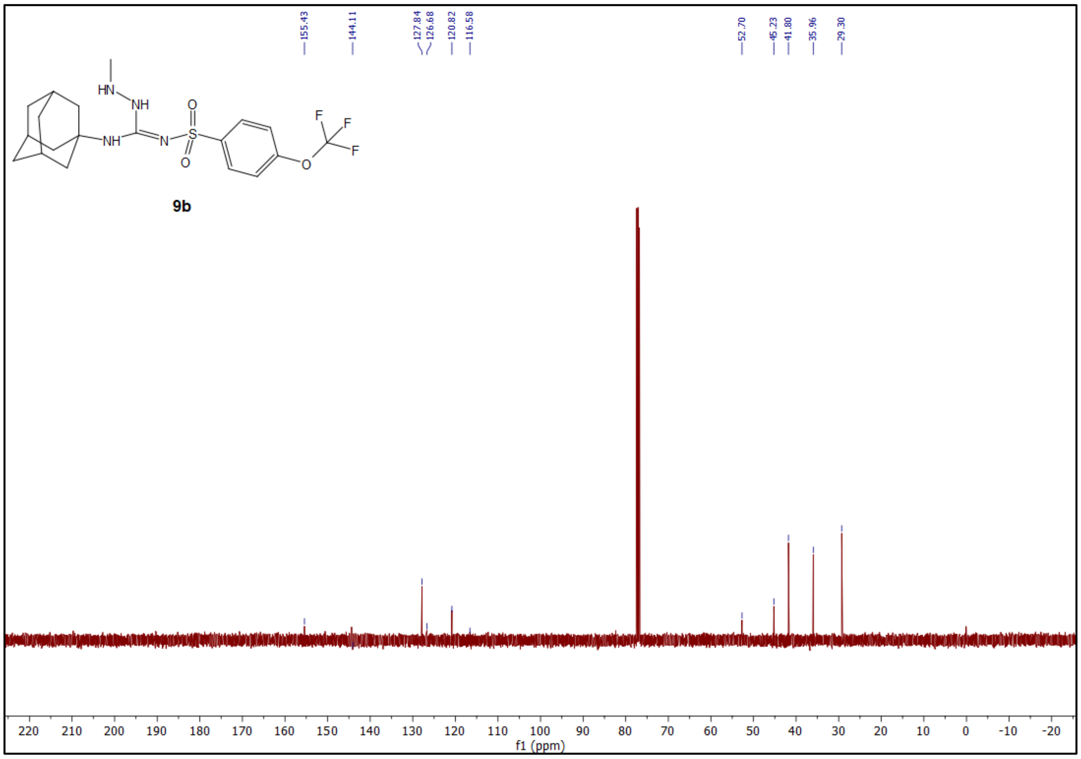

^{1}H NMR of **9c**

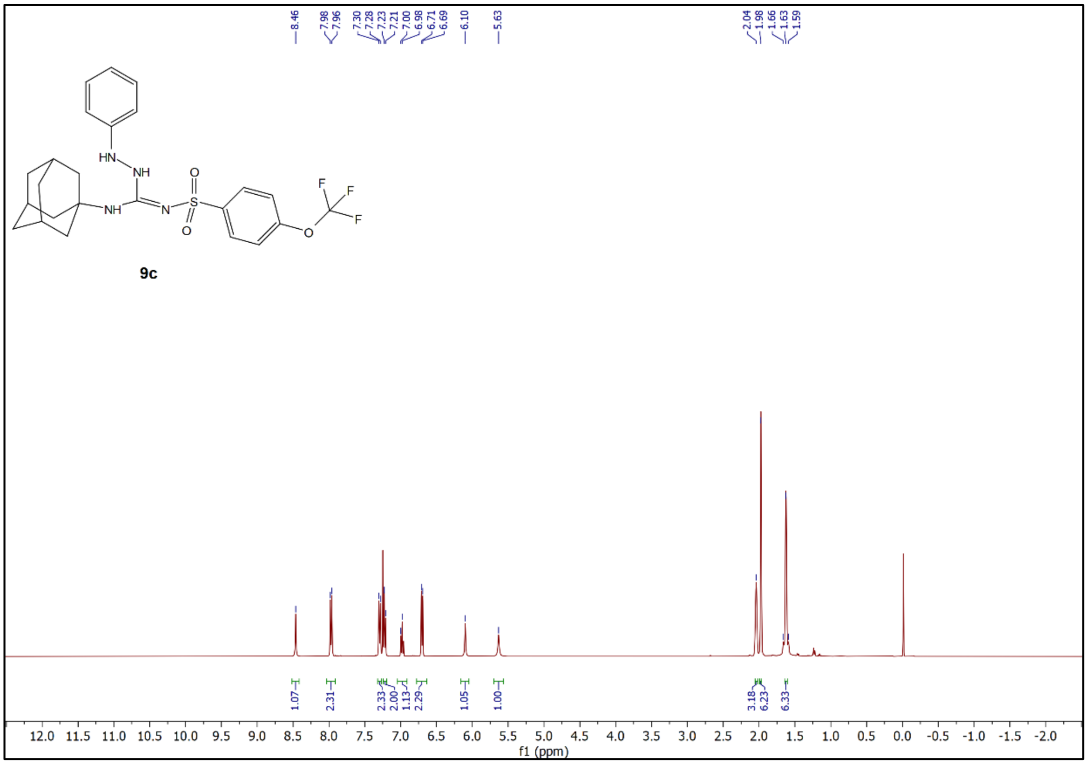

^{13}C NMR of **9c**

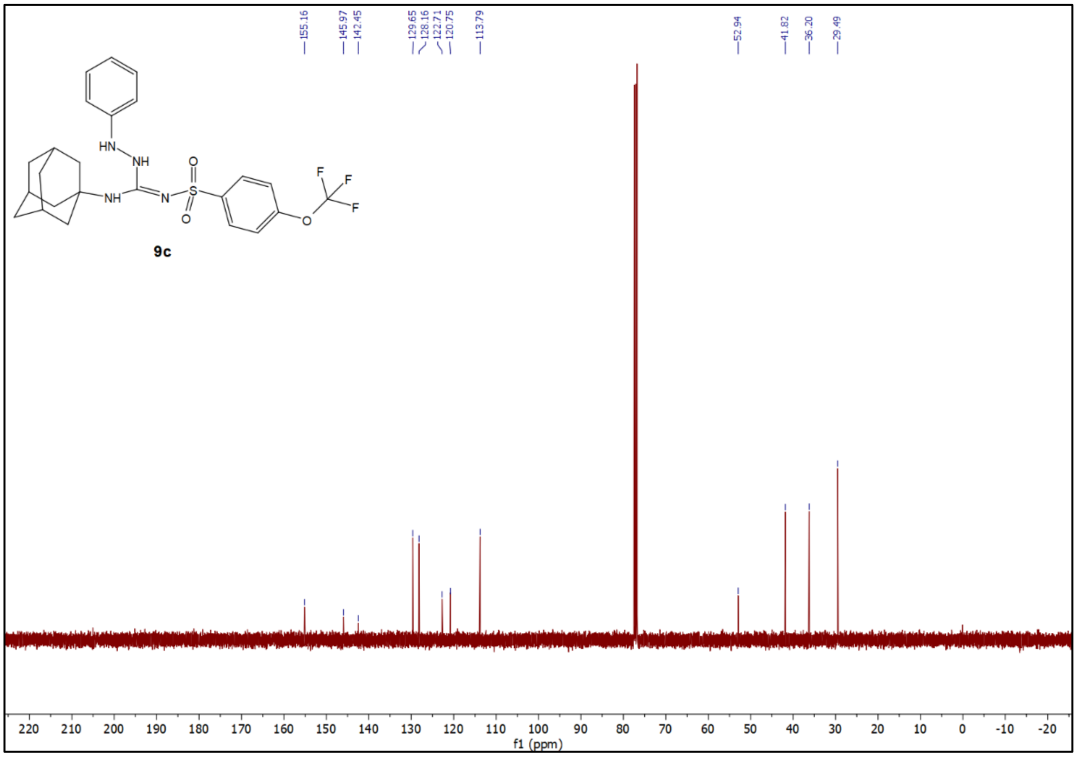

^{1}H NMR of **9d**

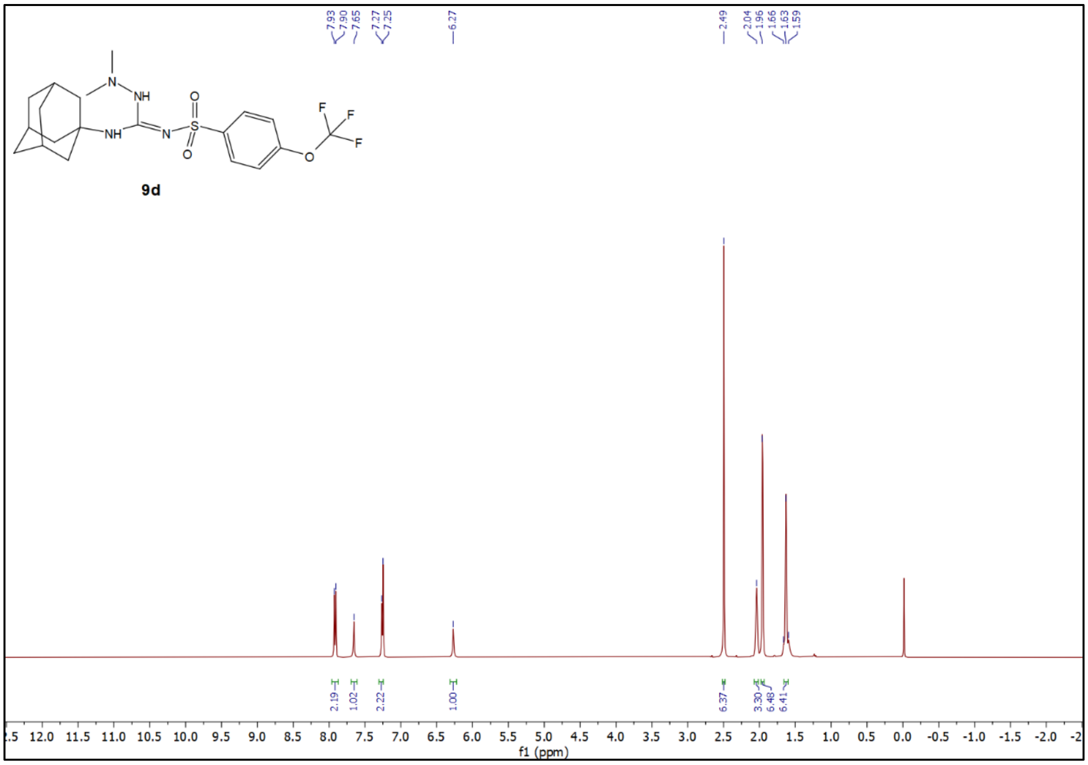

^{13}C NMR of **9d**

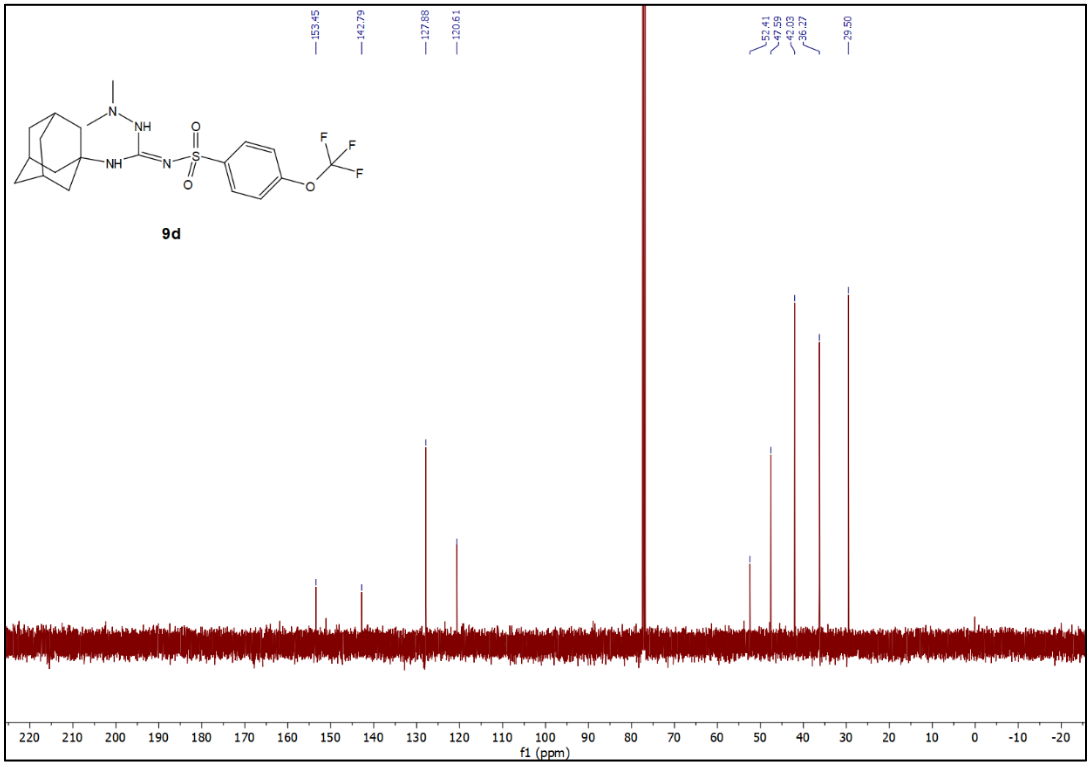

^{1}H NMR of **10a**

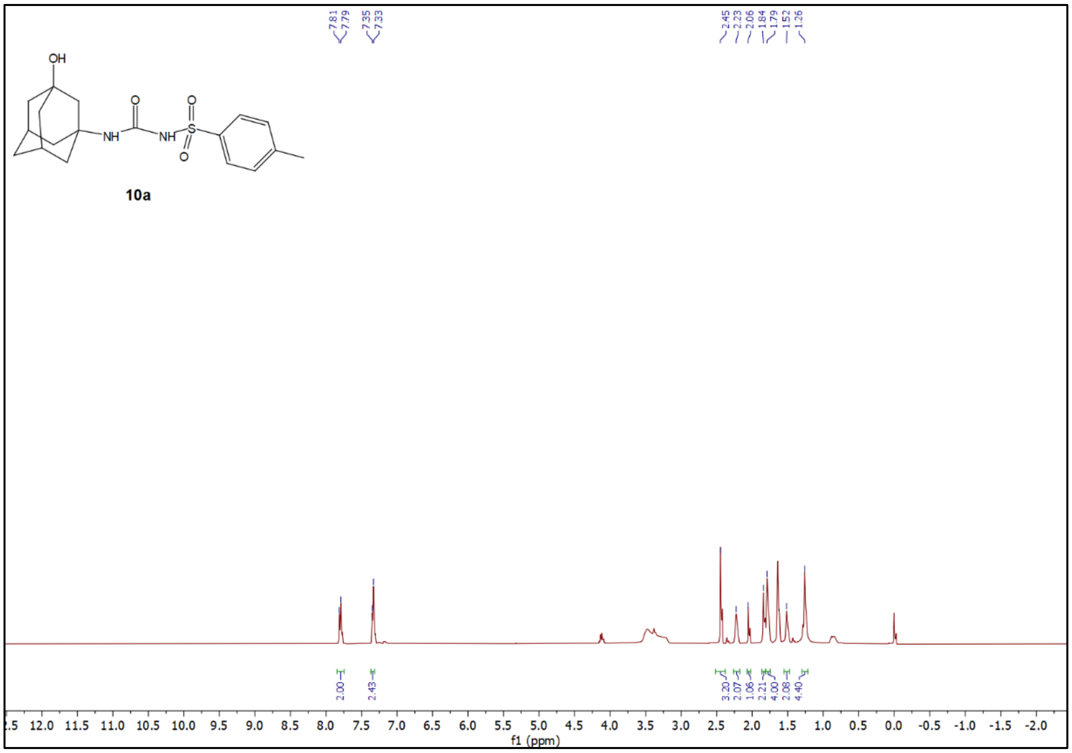

^{13}C NMR of **10a**

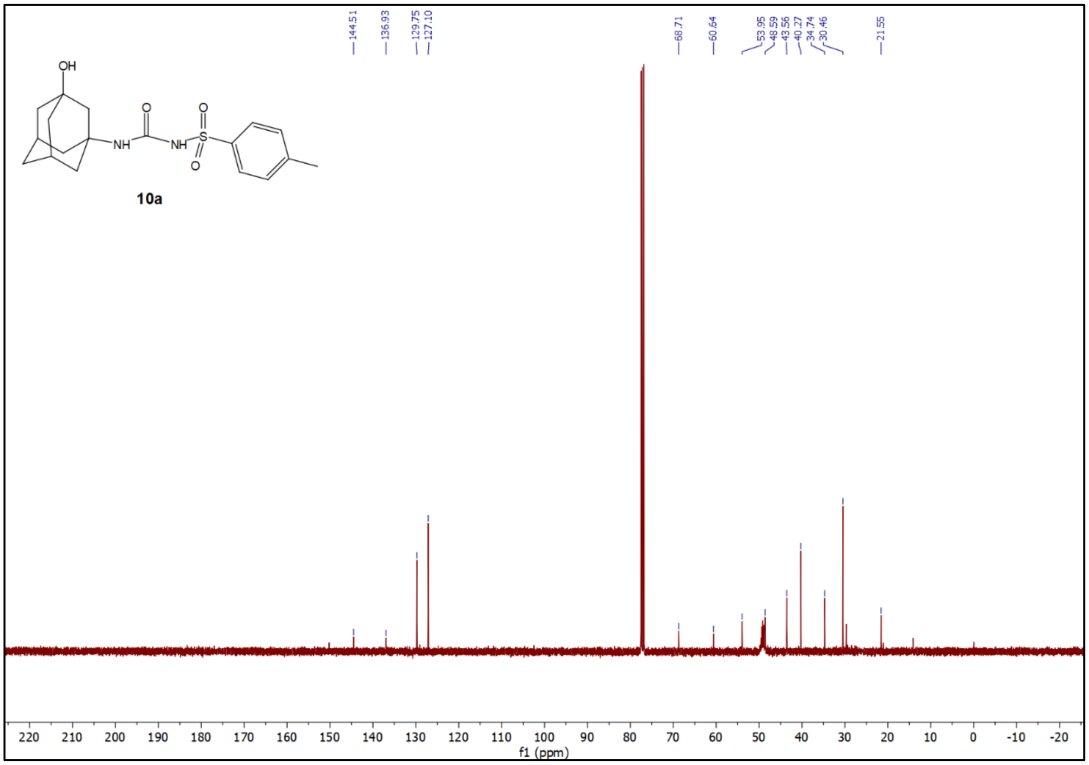

^{1}H NMR of **10b**

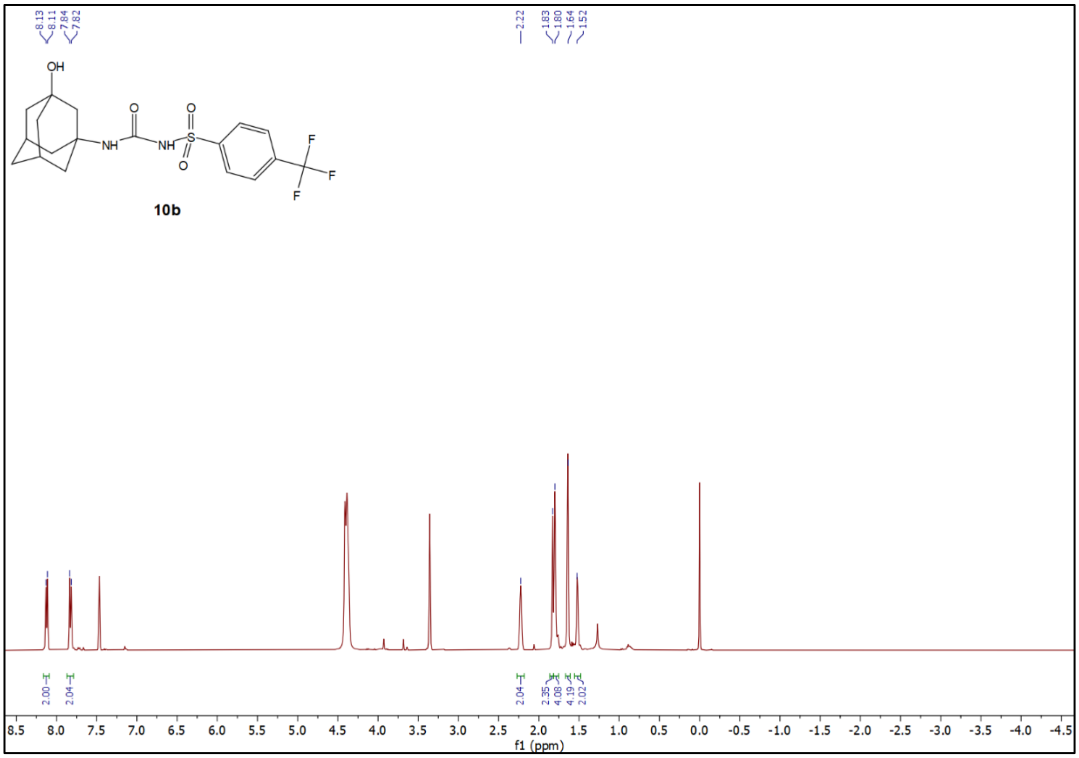

^{13}C NMR of **10b**

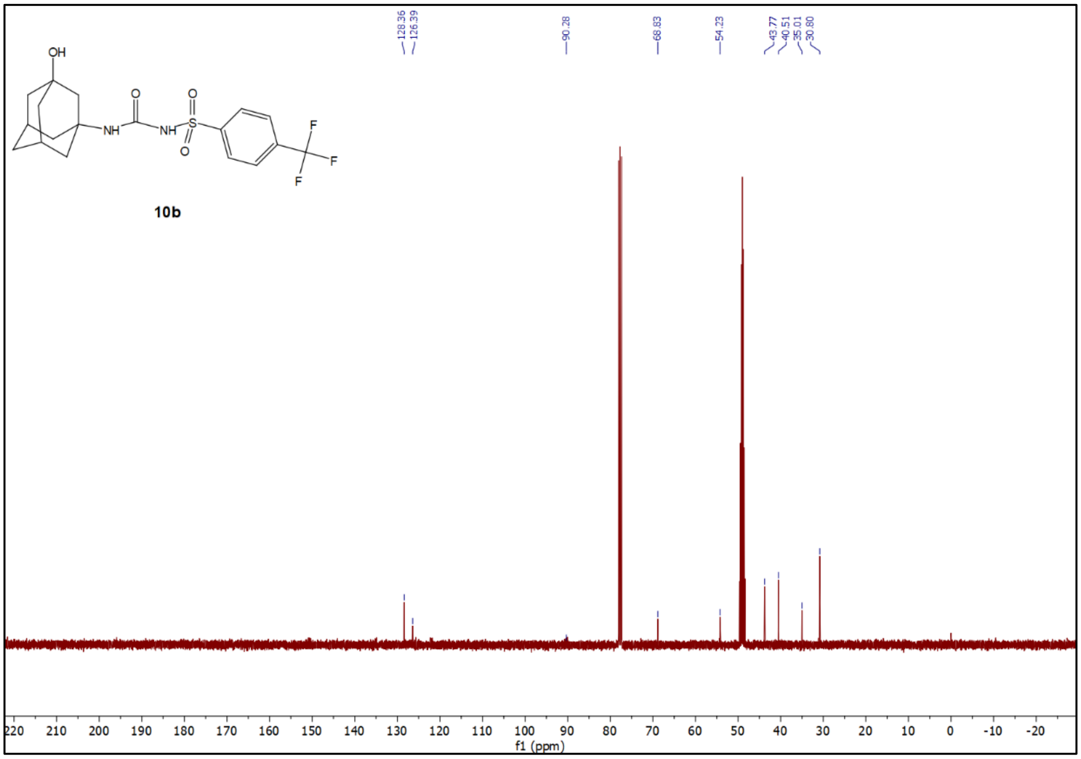

^{1}H NMR of **10c**

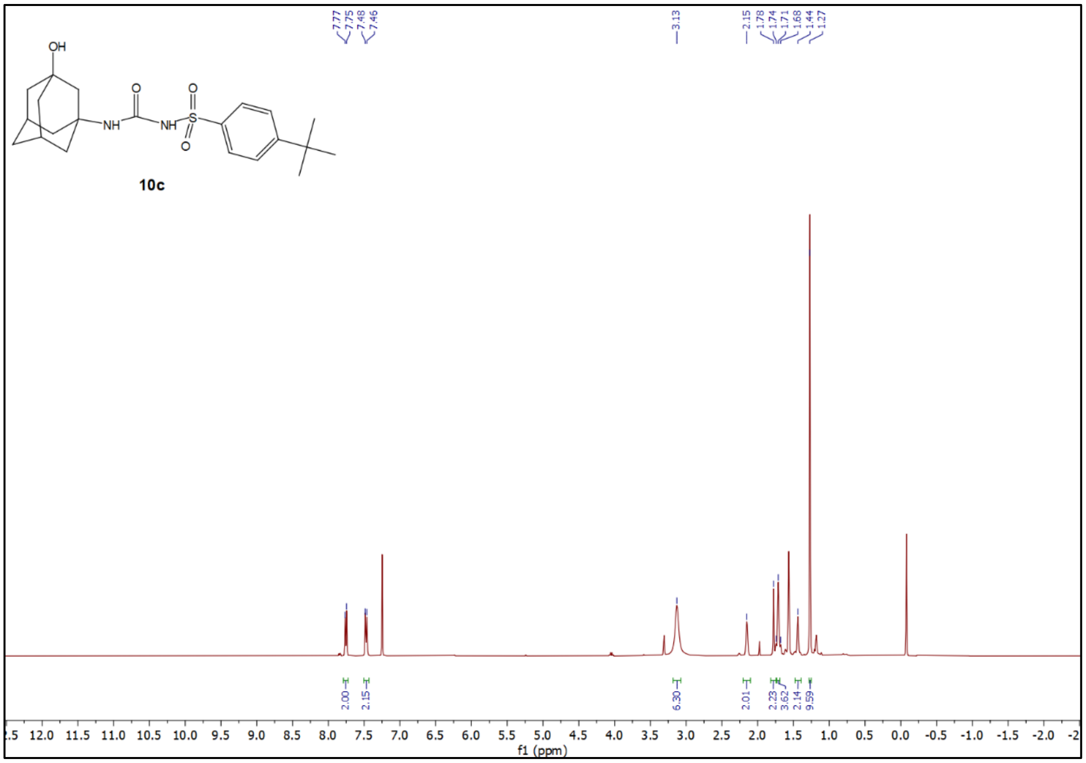

^{13}C NMR of **10c**

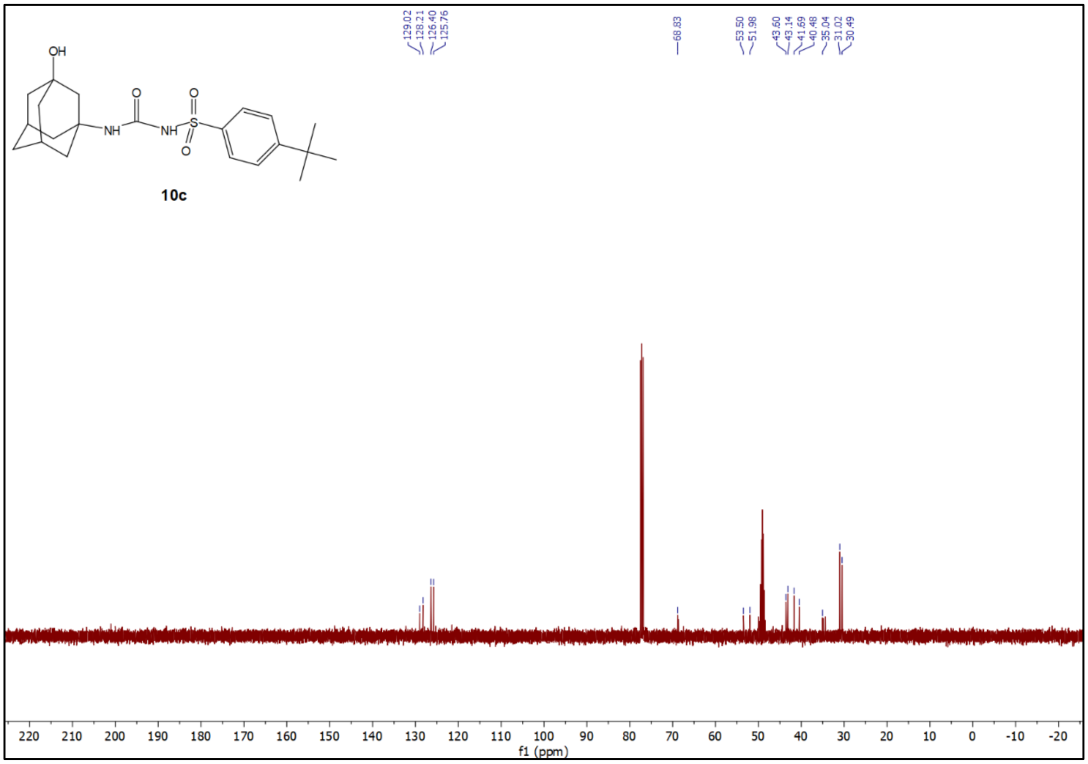

^{1}H NMR of **11a**

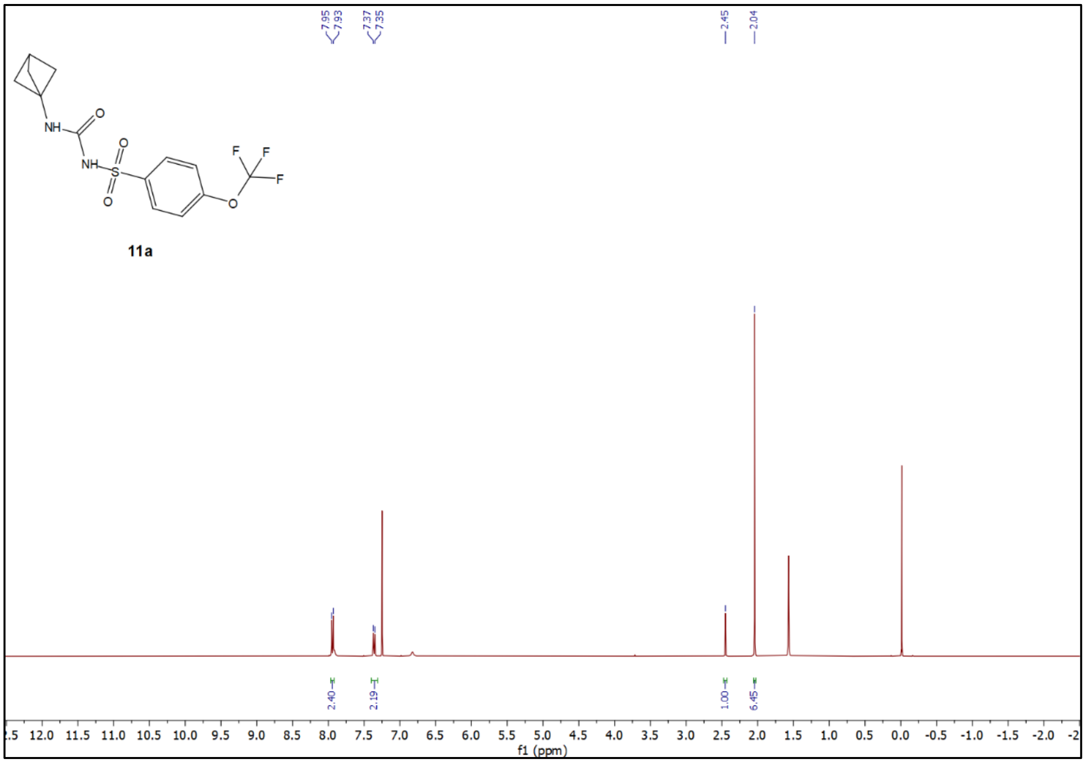

^{13}C NMR of **11a**

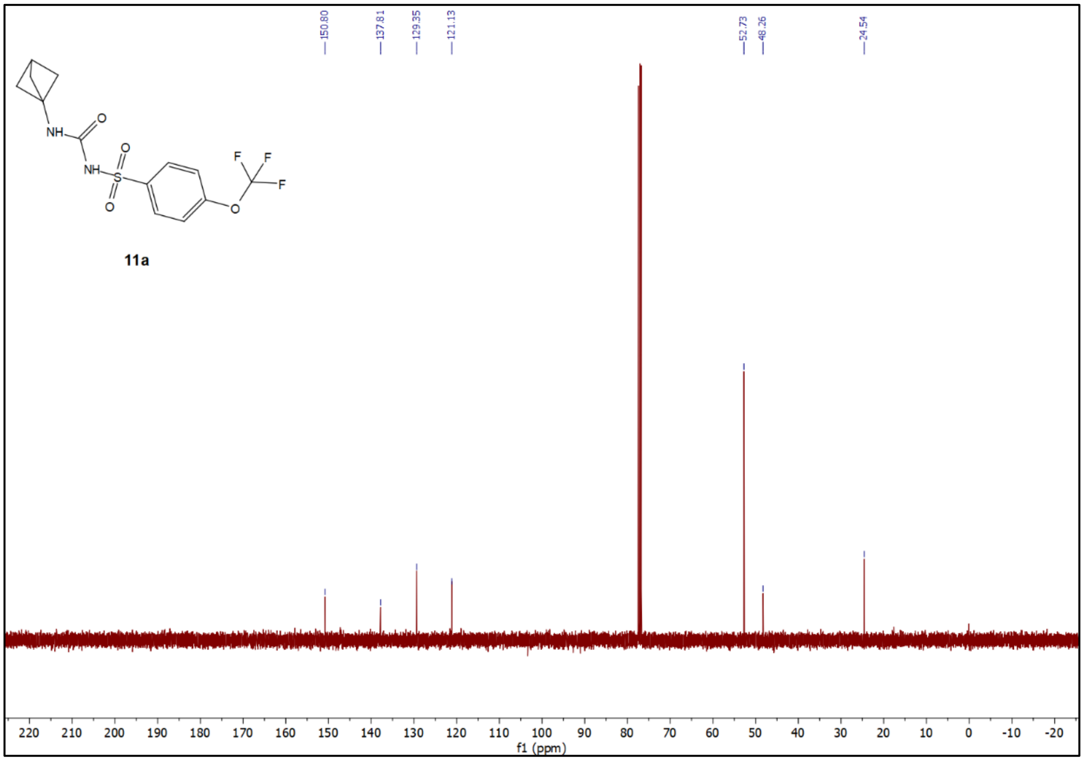

^{1}H NMR of **11b**

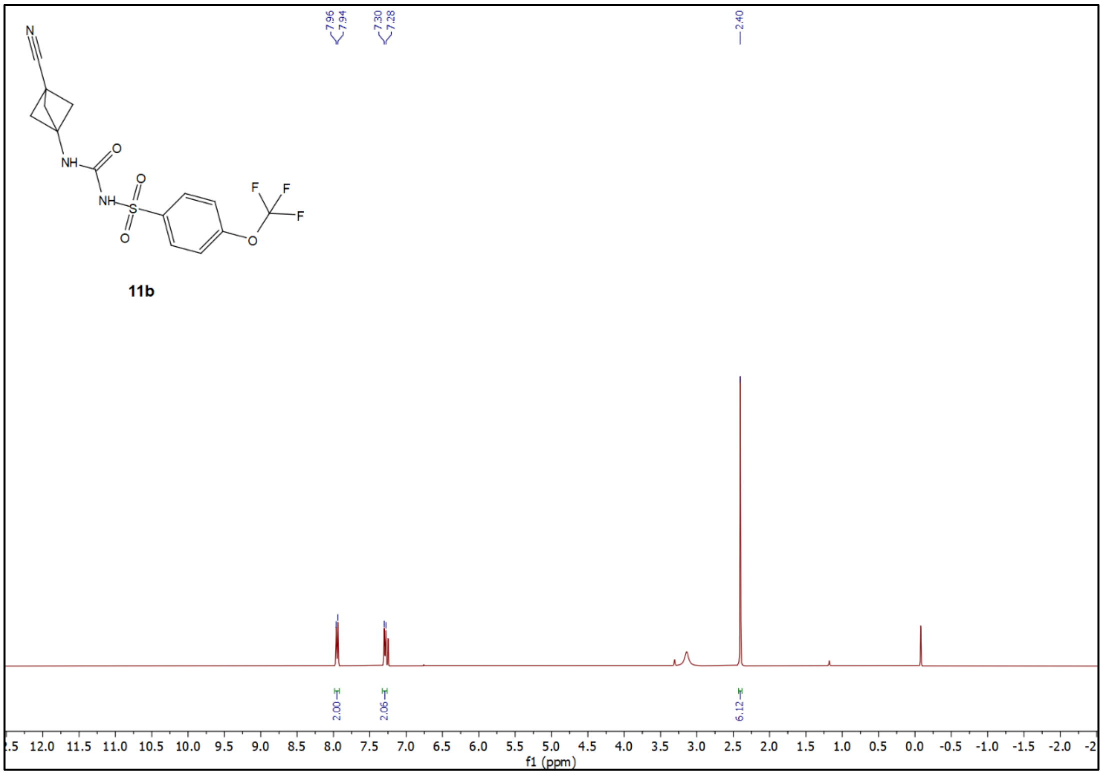

^{13}C NMR of **11b**

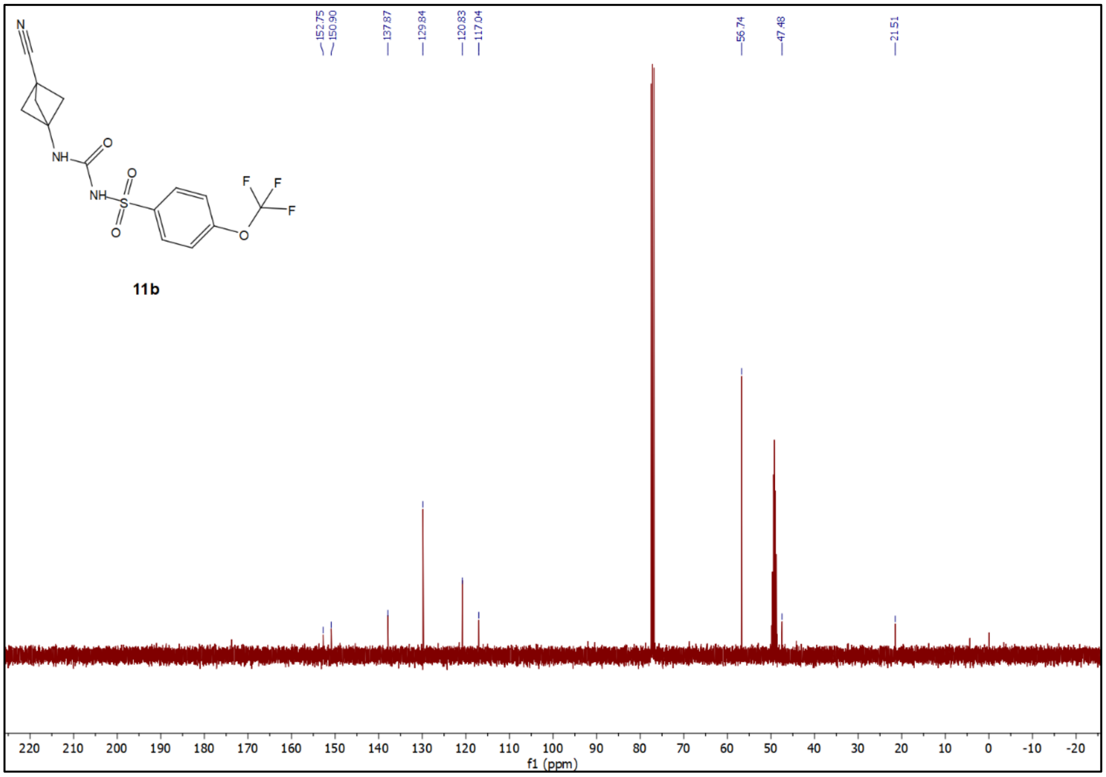

^{1}H NMR of **11c**

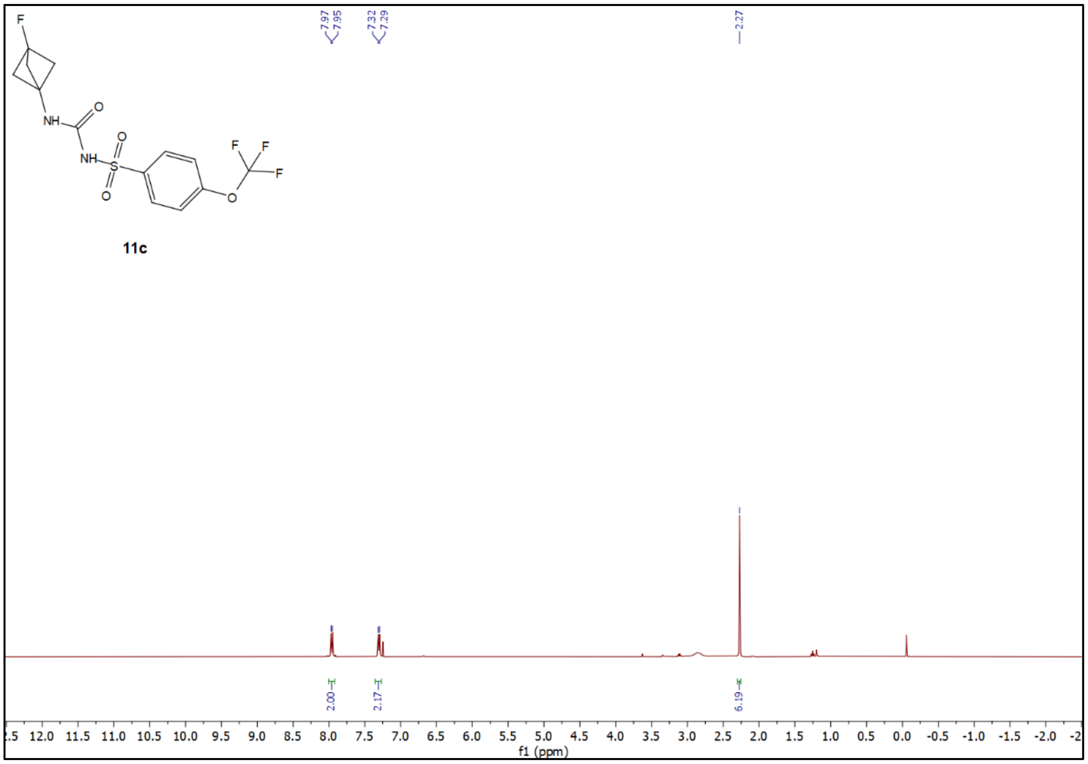

^{13}C NMR of **11c**

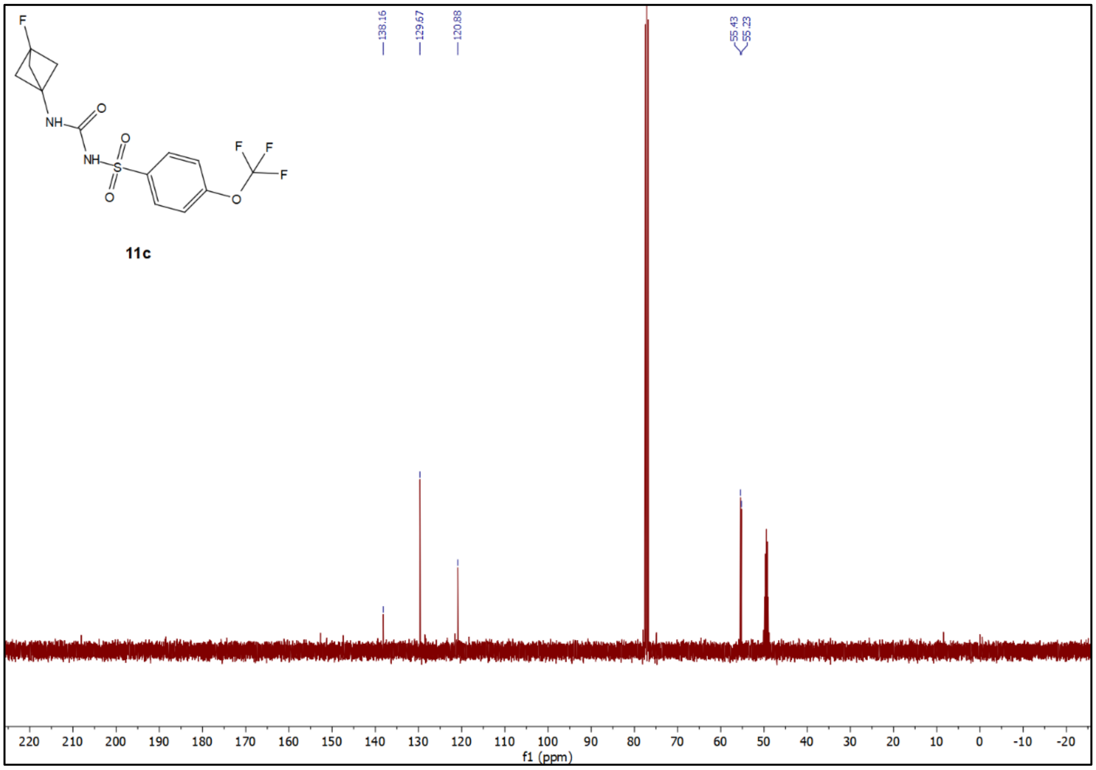

^{1}H NMR of **12**

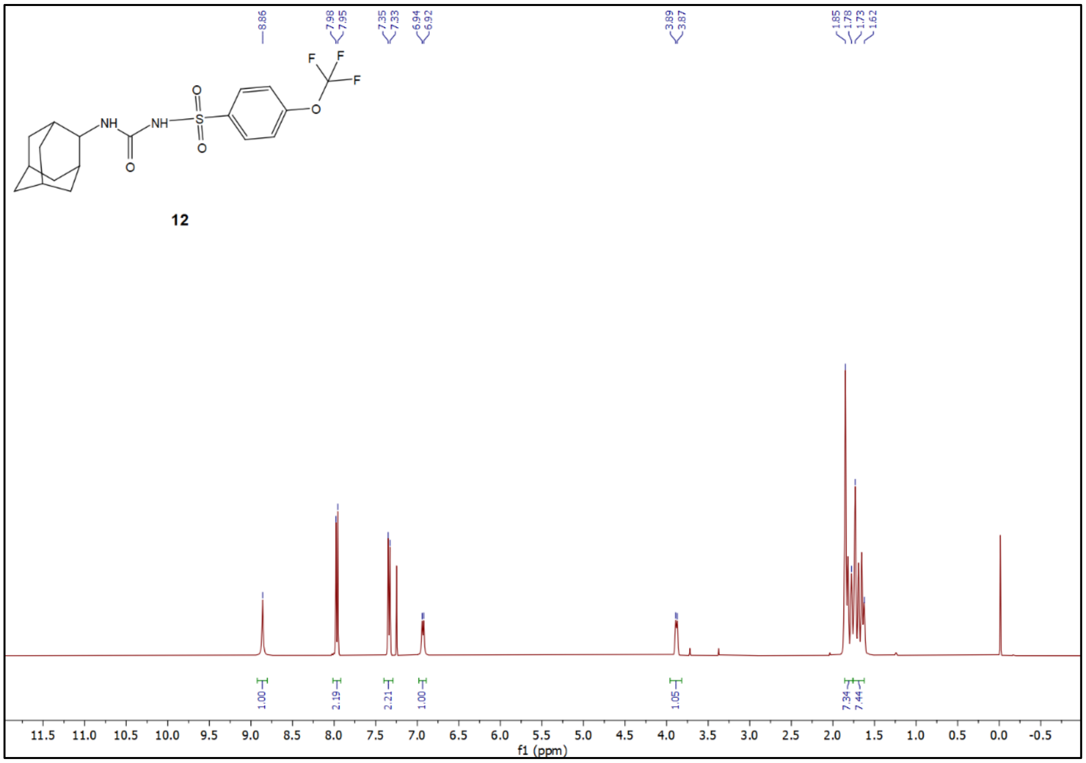

^{13}C NMR of **12**

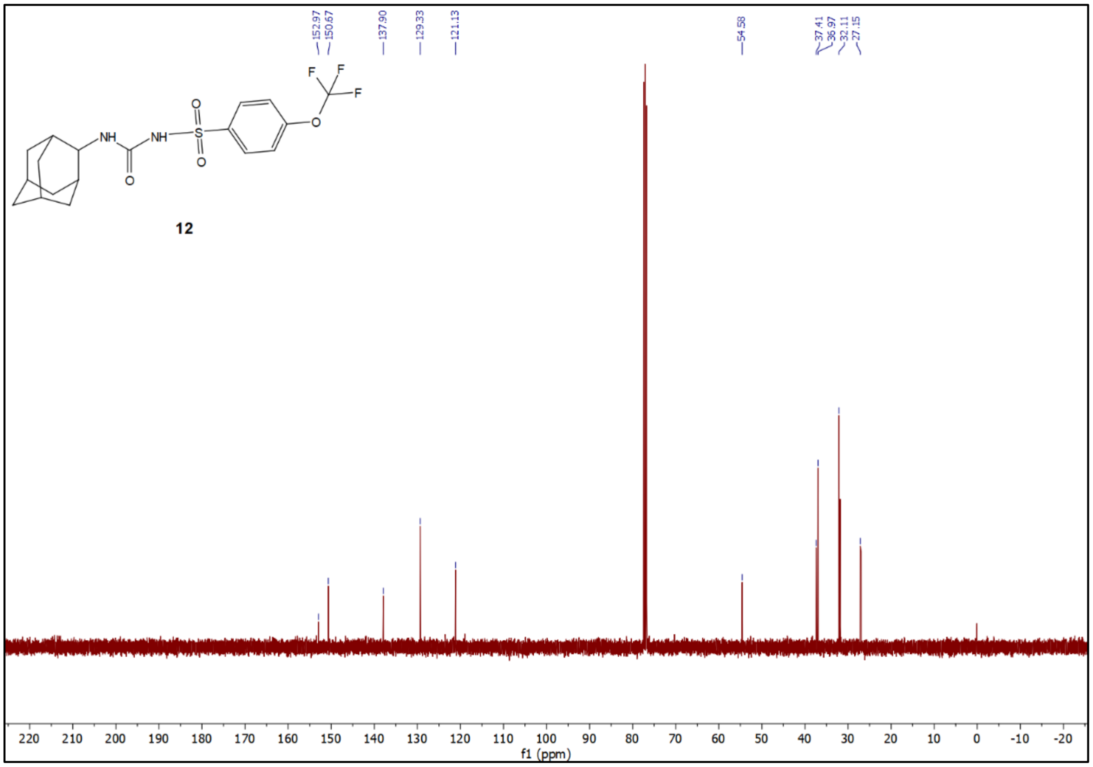

^{1}H NMR of **13**

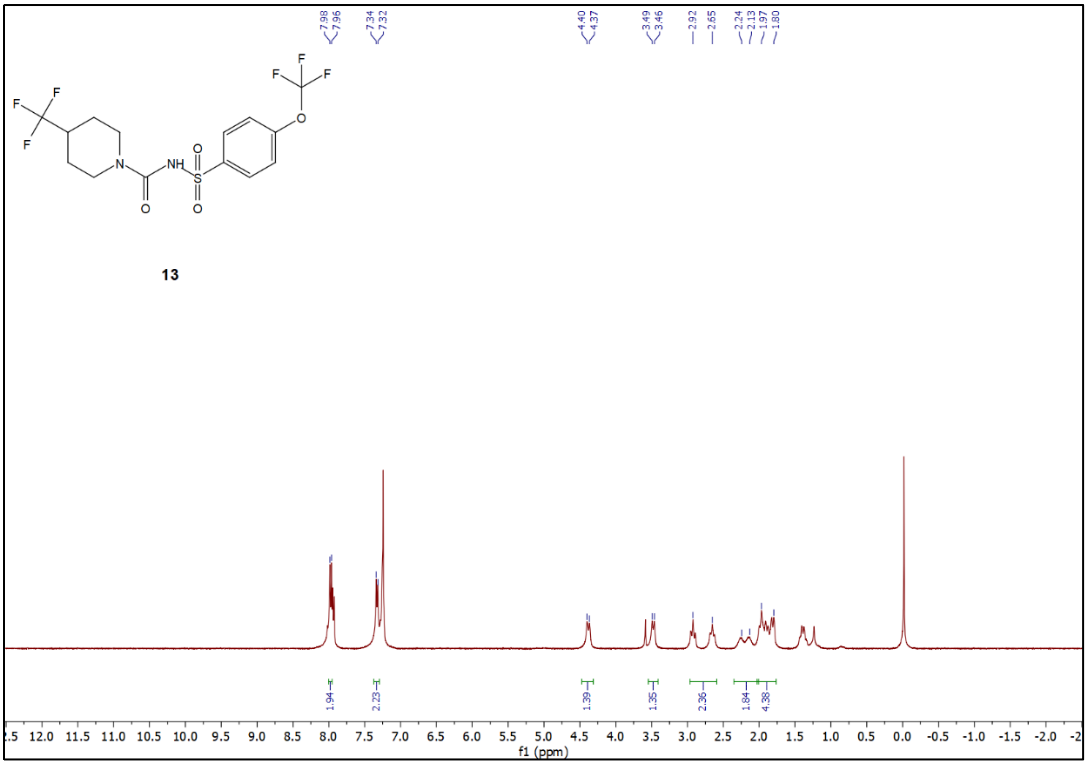

^{13}C NMR of **13**

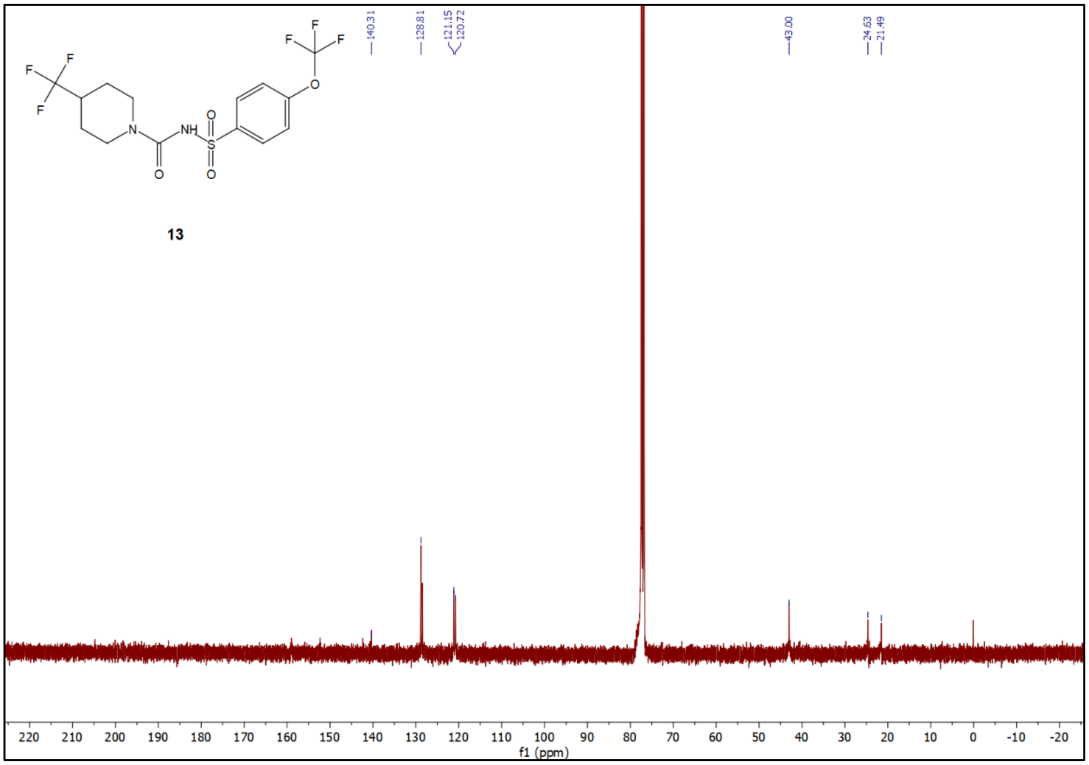

^{1}H NMR of **14**

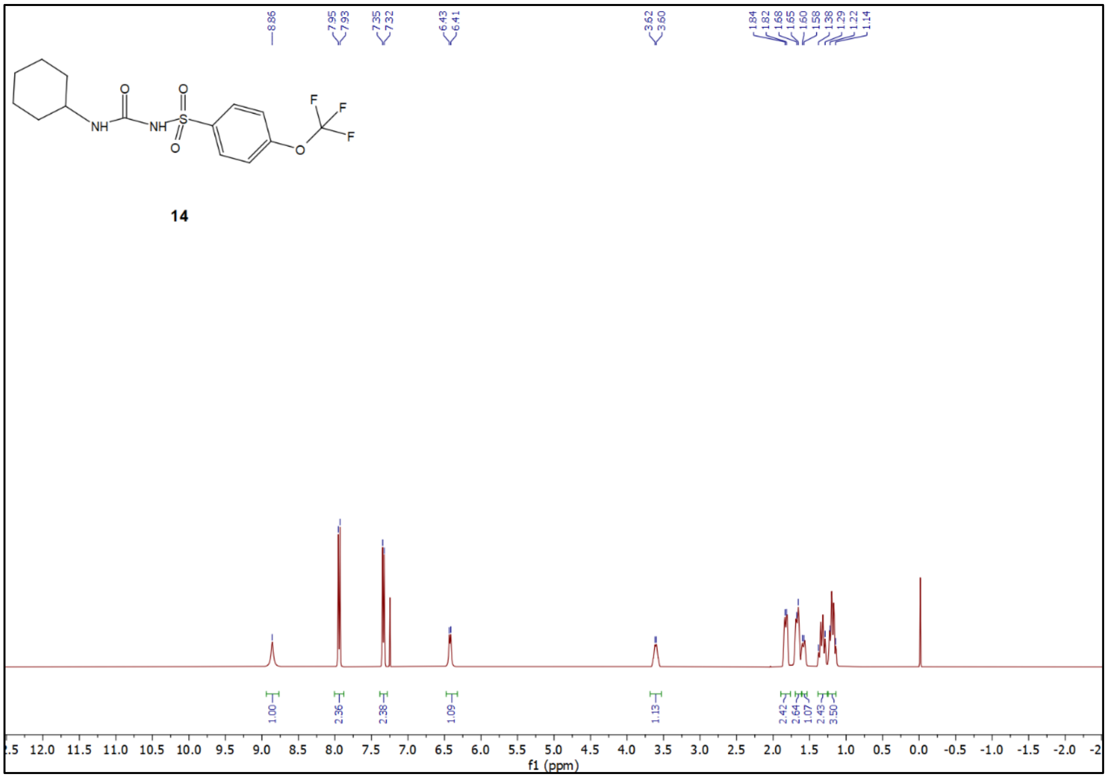

^{13}C NMR of **14**

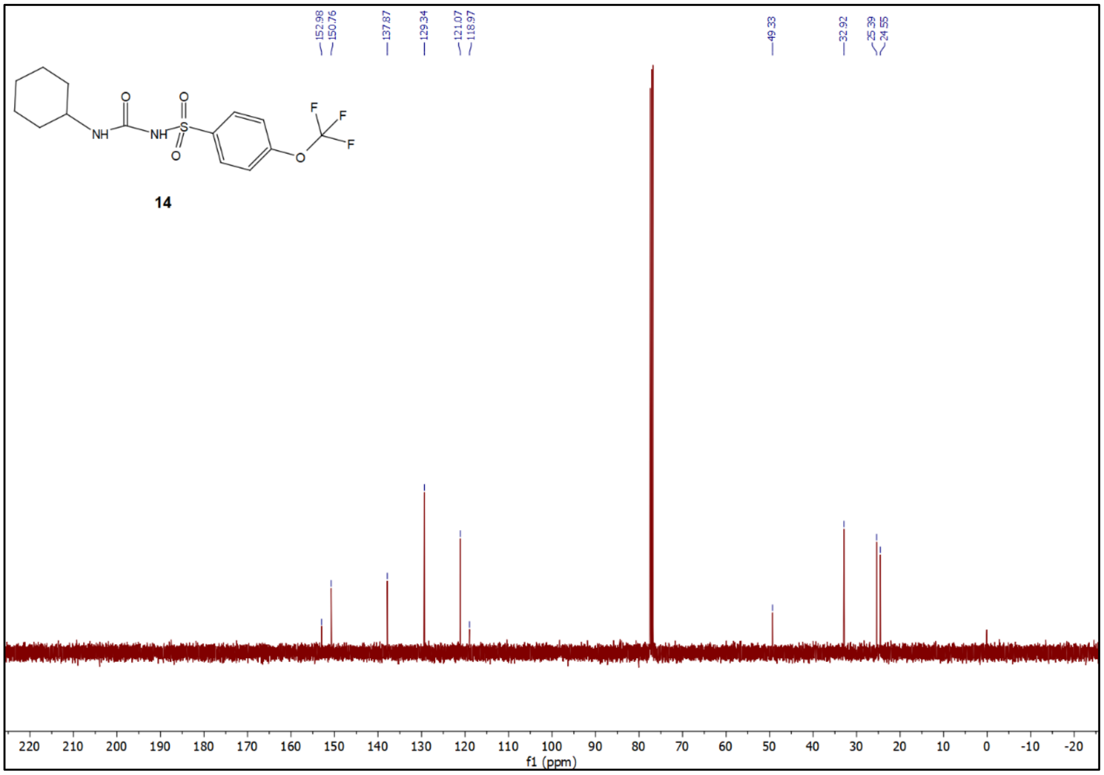

^{1}H NMR of **15a**

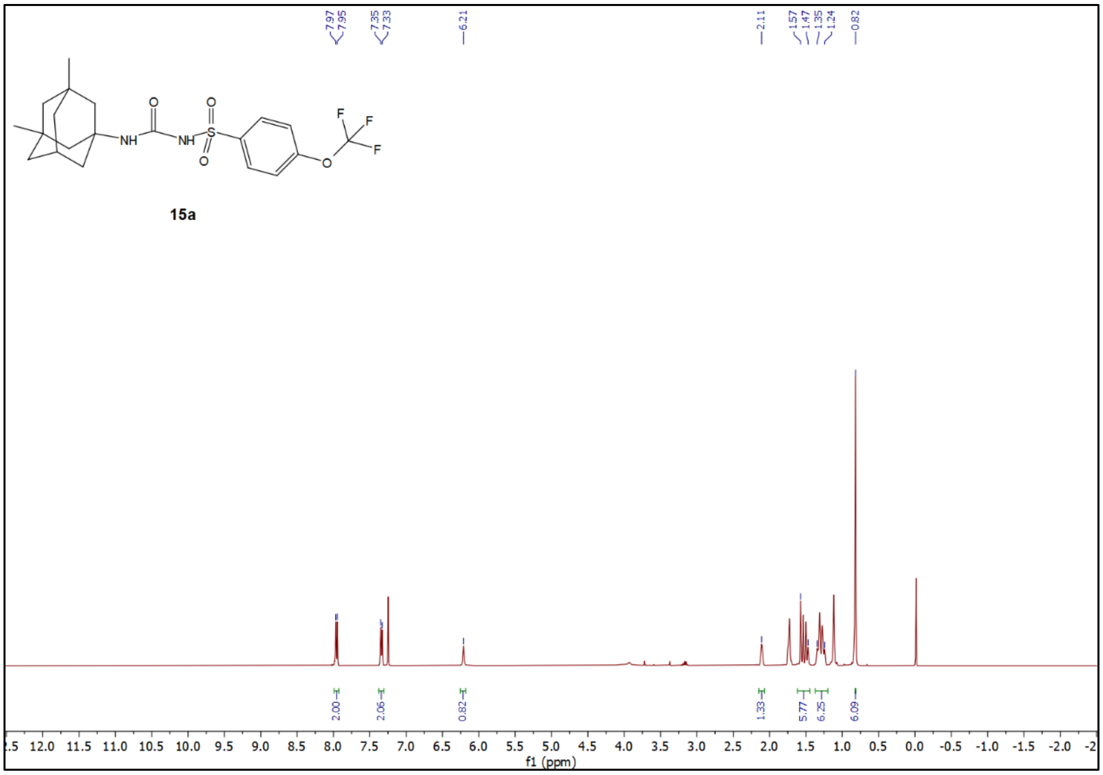

^{13}C NMR of **15a**

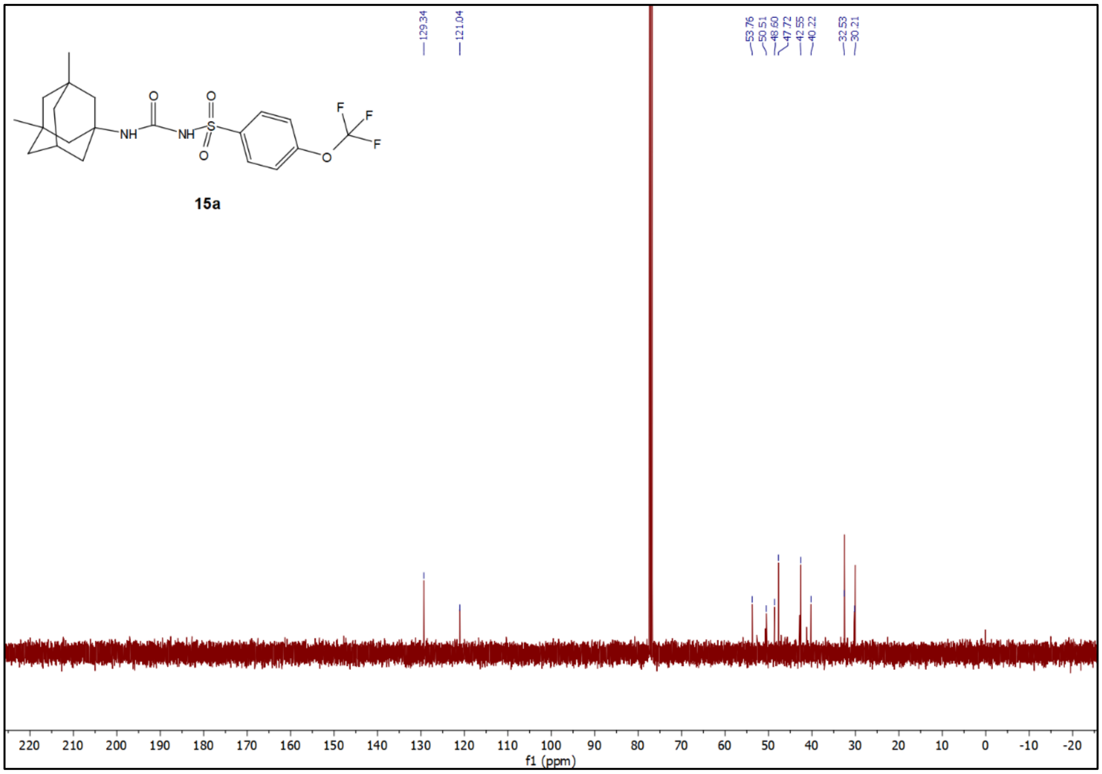

^{1}H NMR of **15b**

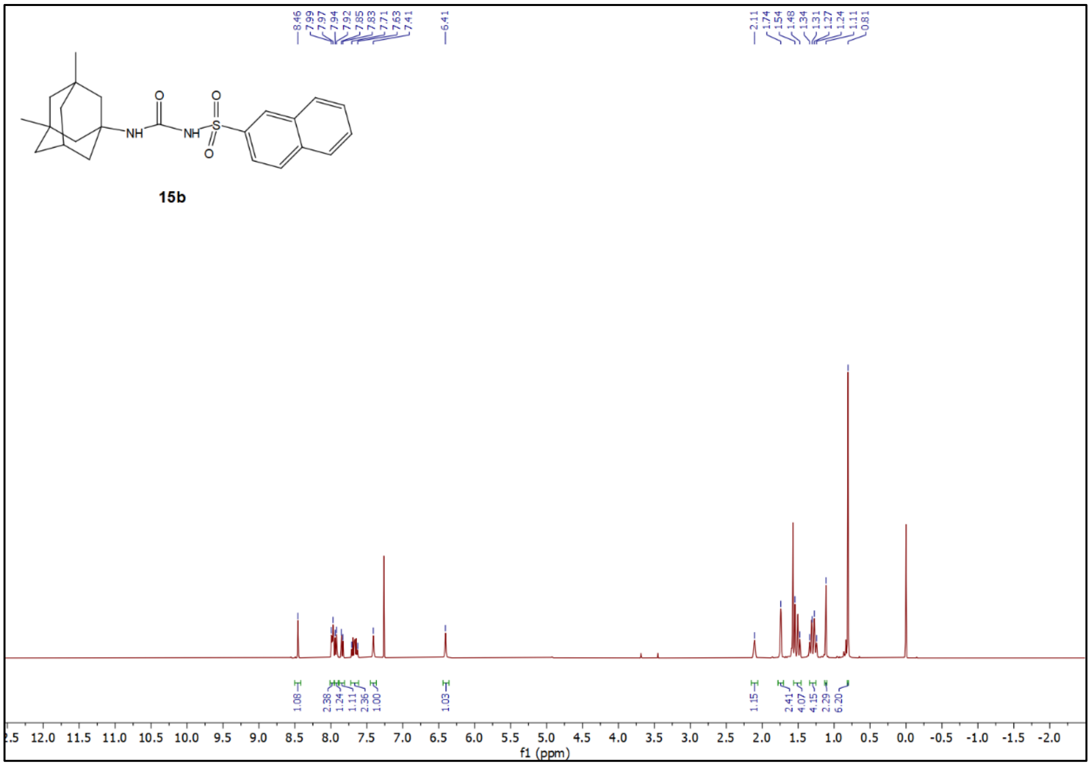

^{13}C NMR of **15b**

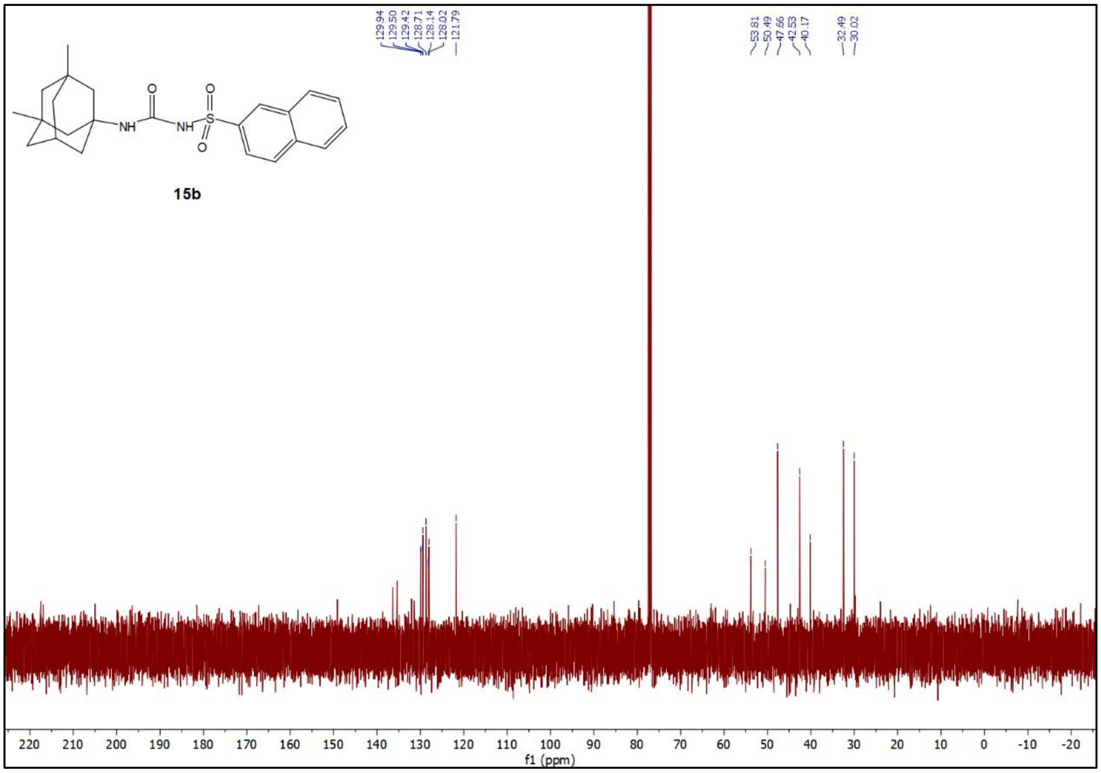

^{1}H NMR of **16**

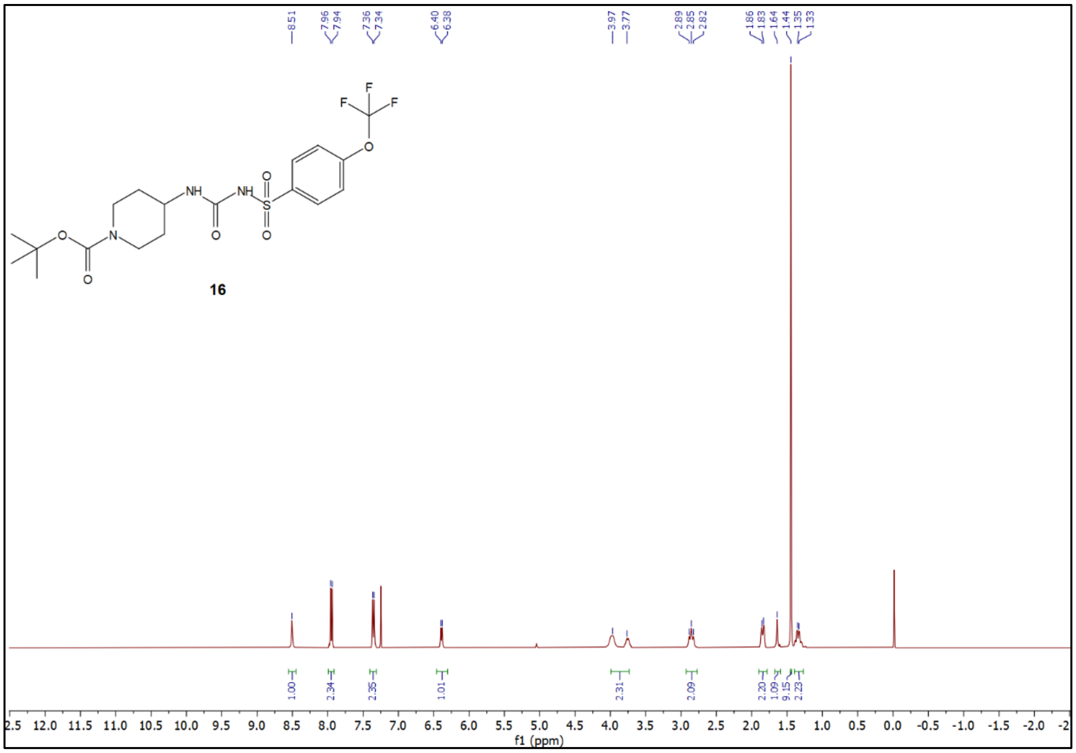

^{13}C NMR of **16**

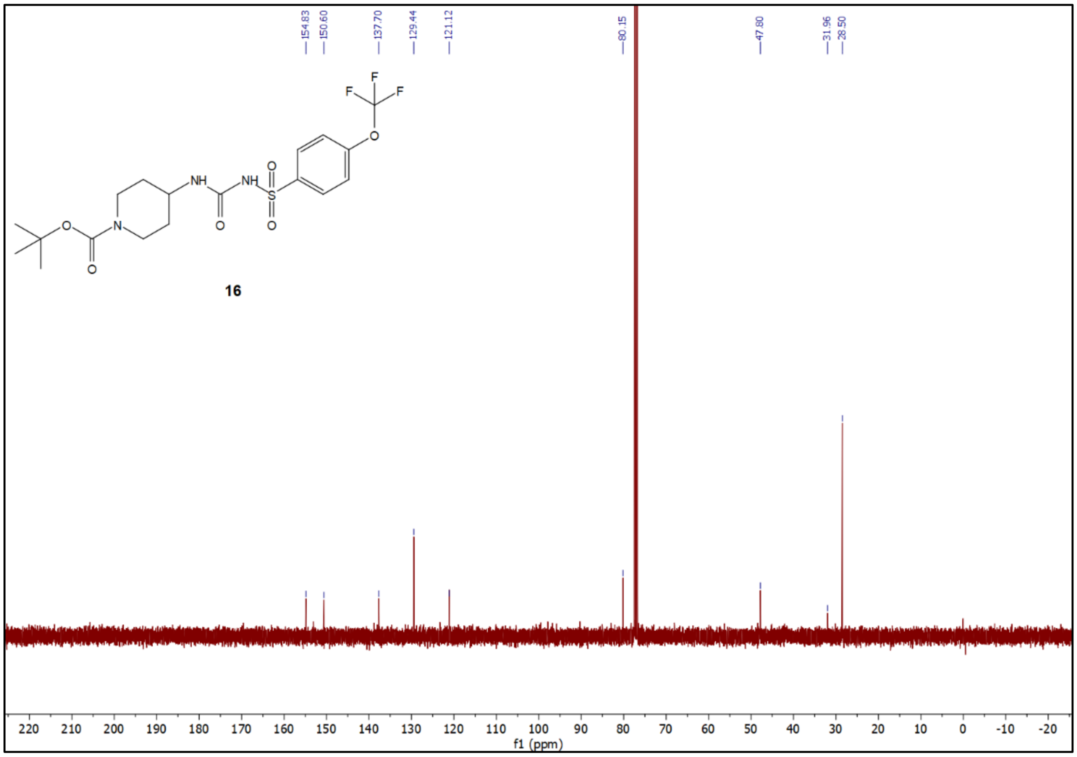

^{1}H NMR of **17**

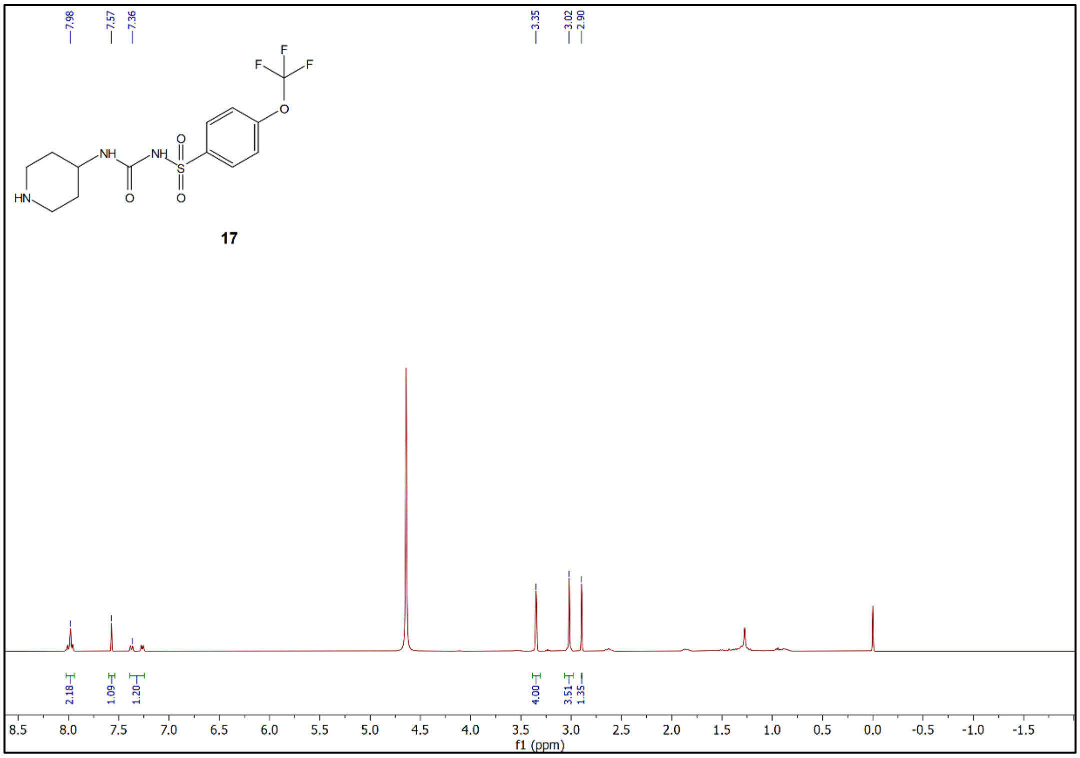

^{13}C NMR of **17**

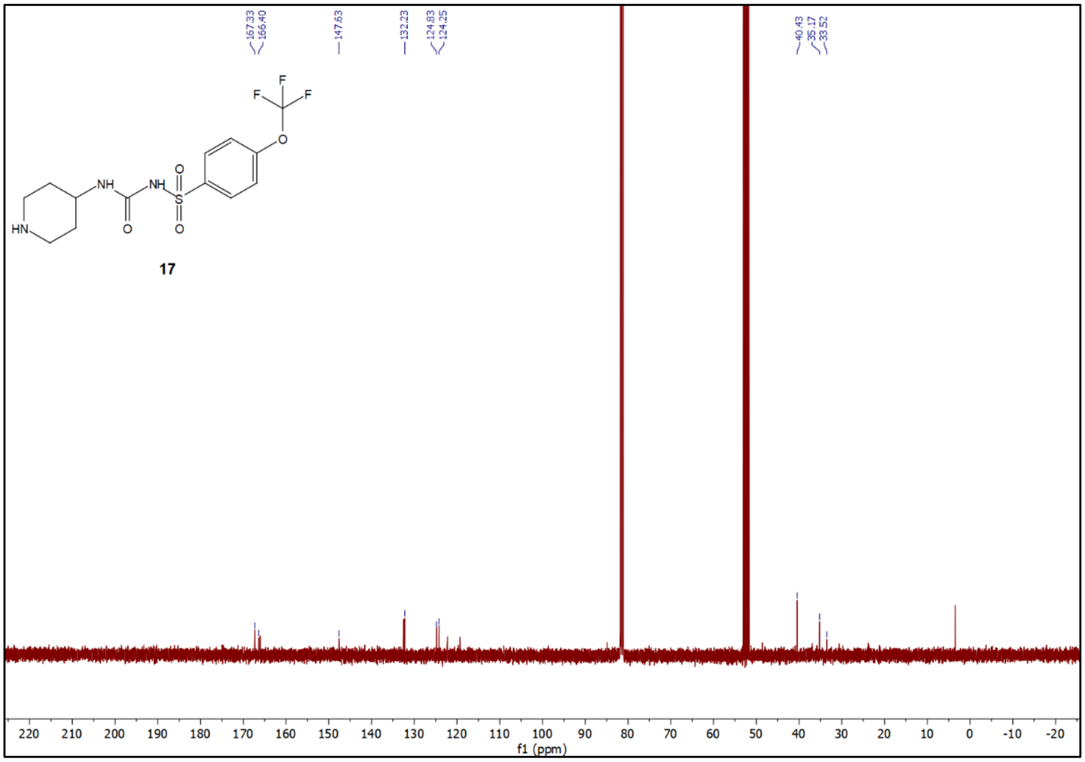

^{1}H NMR of **18**

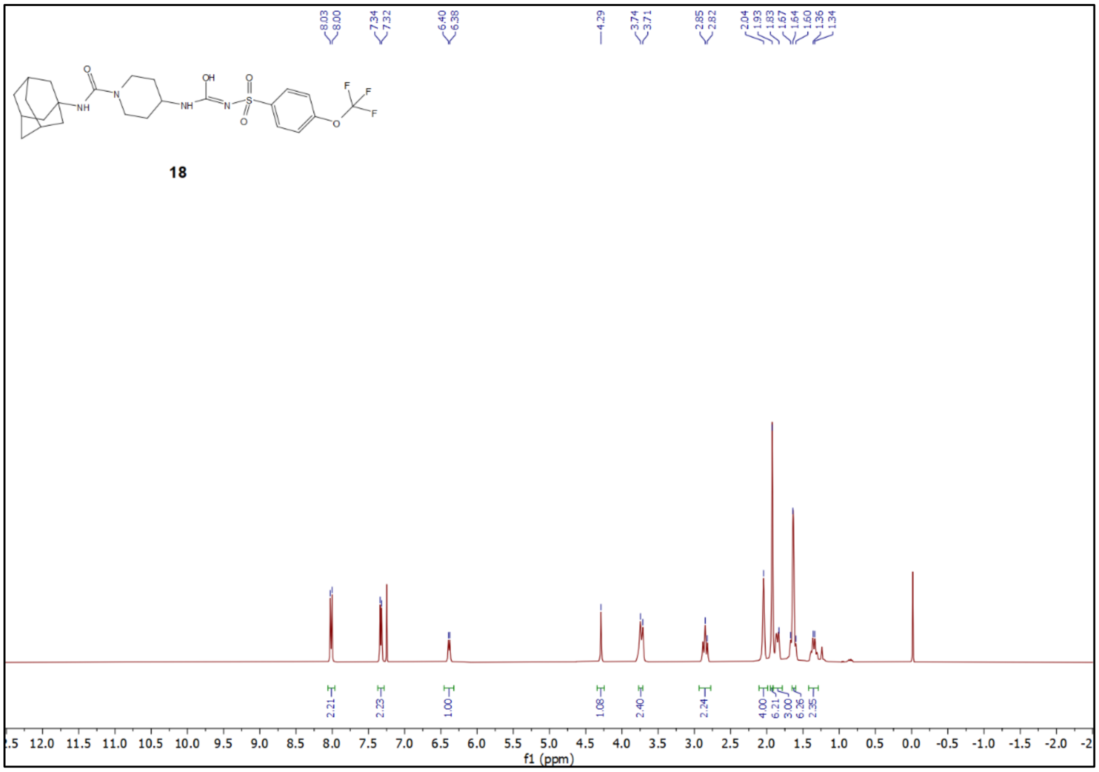
8.03
8.00
7.34
7.32
6.40
6.38
4.29
3.74
3.71
2.85
2.82
2.04
1.93
1.83
1.67
1.64
1.60
1.36
1.34
18
2.21
2.23
1.00
1.08
2.40
2.24
4.00
6.21
3.00
6.26
2.35
f1 (ppm)

^{13}C NMR of **18**

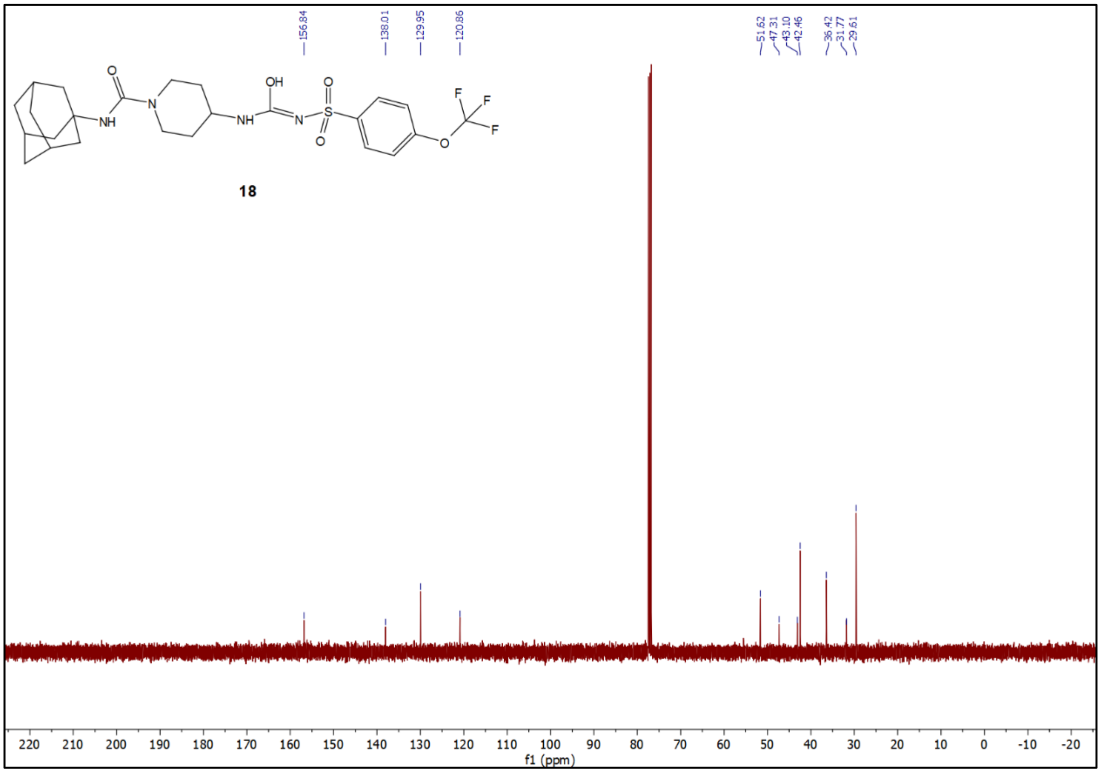
156.84
138.01
129.95
120.86
51.62
47.31
43.10
42.46
36.42
31.77
29.61
18
f1 (ppm)
